# PELP1 coordinates the modular assembly and enzymatic activity of the rixosome complex

**DOI:** 10.1126/sciadv.adw4603

**Published:** 2025-07-25

**Authors:** Jacob Gordon, Andrea M. Kaminski, Saisamhita R. Bommu, Aleksandra Skrajna, Robert M. Petrovich, Lars C. Pedersen, Robert K. McGinty, Alan J. Warren, Robin E. Stanley

**Affiliations:** ^1^ Molecular and Cellular Biology Laboratory, National Institute of Environmental Health Sciences, National Institutes of Health, Department of Health and Human Services, Research Triangle Park, NC 27709, USA.; ^2^ Cambridge Institute for Medical Research, Cambridge Biomedical Campus, Keith Peters Building, Cambridge, CB2 0XY, UK.; ^3^ Department of Haematology, University of Cambridge School of Clinical Medicine, Cambridge Biomedical Campus, Jeffrey Cheah Biomedical Centre, Cambridge, CB2 0AW, UK.; ^4^ Cambridge Stem Cell Institute, Cambridge Biomedical Campus, Jeffrey Cheah Biomedical Centre, Cambridge, CB2 0AW, UK.; ^5^ Genome Integrity and Structural Biology Laboratory, National Institute of Environmental Health Sciences, National Institutes of Health, Department of Health and Human Services, Research Triangle Park, NC 27709, USA.; ^6^ Department of Environmental Sciences and Engineering, Gillings School of Global Public Health, University of North Carolina at Chapel Hill, Chapel Hill, NC 27599, USA.; ^7^ Center for Integrative Chemical Biology and Drug Discovery, Division of Chemical Biology and Medicinal Chemistry, UNC Eshelman School of Pharmacy, University of North Carolina at Chapel Hill, Chapel Hill, NC 27599, USA.; ^8^ Department of Biochemistry and Biophysics, School of Medicine, University of North Carolina at Chapel Hill, Chapel Hill, NC 27599, USA.; ^9^ Lineberger Comprehensive Cancer Center, University of North Carolina at Chapel Hill, Chapel Hill, NC 27599 USA.

## Abstract

The rixosome is a large multisubunit complex that initiates RNA decay during critical nuclear transactions including ribosome assembly and heterochromatin maintenance. The overall architecture of the complex remains undefined because several subunits contain intrinsically disordered regions (IDRs). Here, we combined structural and functional approaches to establish PELP1 as the central scaffold of the rixosome upon which the enzymatic subunits modularly assemble. The C-terminal half of PELP1 is composed of a proline-rich IDR that mediates association with the AAA-ATPase MDN1, histones, and the SUMO-specific protease SENP3. The PELP1 IDR contains a glutamic acid–rich region that we establish can chaperone the histone octamer in vitro. Last, the x-ray structure of a small linear motif (SLiM) from the PELP IDR bound to SENP3 reveals how PELP1 allosterically activates SUMO protease activity. This work provides an integrated structural model for understanding the rixosome’s dynamic architecture and how it modularly coordinates several cellular functions.

## Teaser

The Swiss army knife–like architecture of the rixosome mediates ribosome assembly and polycomb-mediated gene silencing.

## INTRODUCTION

The rixosome has recently emerged as a fundamental RNA processing complex, which plays critical roles in supporting ribosome assembly and heterochromatin maintenance by initiating RNA decay (*1*–*3*). The rixosome is composed of at least six protein subunits including three structural subunits (PELP1, WDR18, and TEX10) and three enzymatic subunits (LAS1L, NOL9, and SENP3) but the overall architecture and stoichiometry of the full complex remains undefined (*4*). The central scaffold and namesake of the rixosome is PELP1 (Rix1 in *Saccharomyces cerevisiae*), a large 120-kDa protein that is enriched in proline, glutamic acid, and leucine residues and is a well characterized proto-oncogene whose expression is dysregulated in many human cancers (*5*, *6*).

The biogenesis of ribosomes is a complex process that relies on hundreds of trans-acting assembly factors to coordinate the processing of the precursor-ribosomal RNA (pre-rRNA) with the assembly of the ribosomal subunits (*7*–*9*). The human rixosome components were originally identified by co-immunoprecipitations (co-IPs) and shown to copurify with pre-60S ribosomes (*2*, *10*). Individual small interfering RNA knockdown of the subunits revealed that they are all important for processing the internal transcribed spacer 2 (ITS2) sequence from the pre-rRNA (*2*). LAS1L was subsequently identified as the endoribonuclease that cleaves the ITS2, generating the 12S pre-rRNA with a 2′-3′ cyclic phosphate and the 28.5S pre-rRNA with a 5′OH (*11*). The polynucleotide kinase (PNK) NOL9 phosphorylates the 5′OH product, which triggers exoribonuclease decay by XRN2 to generate the mature 28S rRNA (*11*). LAS1L and NOL9 form a tetrameric module (2LAS1L:2NOL9) within the rixosome, known as ribonuclease (RNase) PNK. The architecture of RNase PNK facilitates the enzymatic coordination of the LAS1L nuclease with the NOL9 kinase (*12*–*15*), but little is known about how this module is incorporated within the rixosome (*3*, *16*).

Recent work revealed that in addition to ITS2 processing, the rixosome triggers nascent mRNA decay at facultative heterochromatin, a compact form of chromatin characterized by an abundance of repressive histone marks (*1*, *17*). Repressive histone marks are made by chromatin modification enzymes including polycomb repressive complex 1 (PRC1) and PRC2, which control transcription and gene expression of developmental genes (*18*). Polycomb complexes have conserved catalytic cores that associate with a large number of accessory and regulatory modules (*19*). The rixosome was recently shown to colocalize with PRC1 and PRC2 at facultative heterochromatin and the catalytic activity of the RNase PNK module is required for initiating the decay of synthesized RNA from facultative heterochromatin (*1*). Work in *Schizosaccharomyces pombe* demonstrated that the rixosome promotes heterochromatin inheritance, suggesting that there is an evolutionarily conserved role for the rixosome in mediating RNA decay at heterochromatin (*20*, *21*). Collectively, this work supports that the RNA processing activity of the rixosome is critical for at least two nuclear RNA processing events, but how the complex is recruited to its different RNA targets is unknown.

In addition to RNase PNK, the other enzymatic module of the rixosome is SENP3, a SUMO-specific protease (*22*). SUMOylation is a dynamic post-translational modification that is known to regulate protein functions including protein-protein interactions and subcellular localization (*22*). Several members of the rixosome complex are modified by SUMO and inactivation of SENP3 disrupts ribosome assembly (*10*, *23*–*26*). Previous work established that SENP3-driven deconjugation of SUMO from PELP1 is critical for supporting pre-60S assembly steps involving the AAA [adenosine triphosphatases (ATPases) associated with diverse cellular activities]-ATPase MDN1 (*24*). SENP3 is also important for rixosome mediated gene silencing, as it was recently shown that SENP3 is required to deSUMOylate rixosome subunits for the complex to associate with PRC1 (*27*). Dysregulation of SENP3 has been linked to many human diseases including cancer, heart disease, and neurological disorders, yet very little is known about what regulates SENP3’s SUMO protease activity (*22*, *28*).

**Fig. 1.**
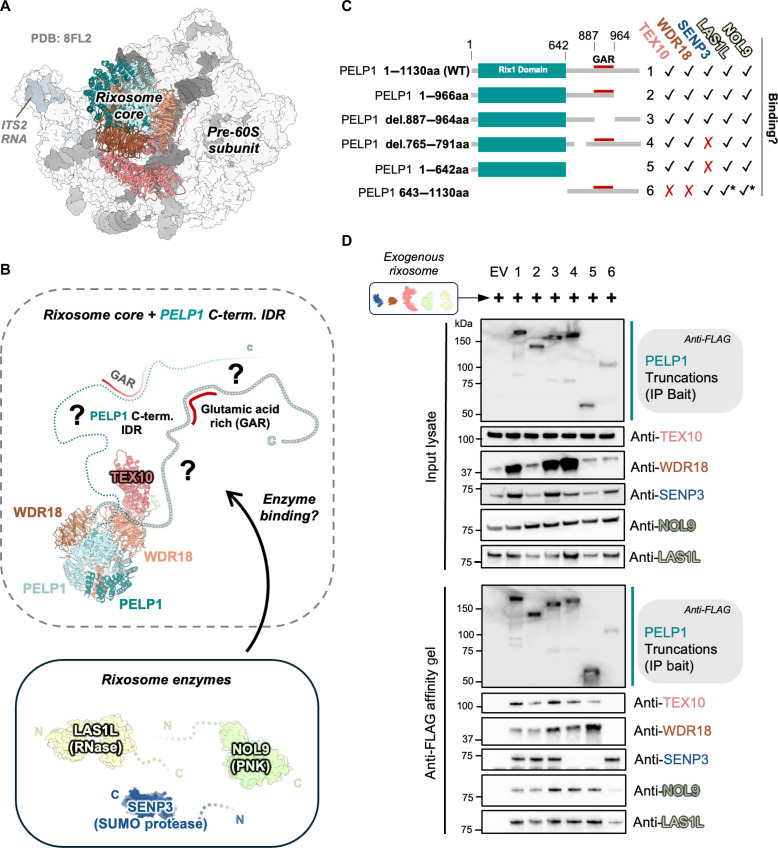
PELP1 is a critical scaffold for rixosome assembly. (**A**) Cryo-EM structure of the human pre-60S ribosome with the rixosome bound [PDB ID:8FL2 (*3*)]. Only the conserved scaffolding core of the rixosome (PELP1 Rix1 domain, WDR18, and TEX10) is visible in the structure. Also see fig. S1. The rixosome core is formed by two copies of the PELP1 Rix1 domain (shades of teal), two copies of WDR18 (shades of orange), and one copy of TEX10 (pink). (**B**) Structure of the rixosome core indicating the putative positions of the PELP1 C-terminal IDRs. It remains unclear how the three rixosome enzyme LAS1L (ribonuclease, RNase), NOL9 (poly-nucleotide kinase, PNK), and SENP3 (SUMO protease) associate with the rixosome core. (**C**) Schematic of C-terminal FLAG-tagged PELP1 variant/truncation constructs (1 to 6) used to affinity purify the rixosome in the co-immunoprecipitation (co-IP) experiment displayed in (D). Checkmarks and X marks denote qualitative binding results for specific rixosome components to the PELP1 variant used for reconstitution and co-IP. * denotes a notable decrease (but still detectable) in signal for specific rixosome components. (**D**) SDS-PAGE and Western blot using antibodies for the endogenous rixosome proteins qualitatively identified the presence or absence of rixosome proteins (endogenous and exogenous) upon co-IP from human cells of PELP1 variants in (C). EV denotes empty vector control.

Recent structures determined by cryogenic electron microscopy (cryo-EM) have revealed the architecture of the scaffolding core of the human rixosome complex both on and off pre-ribosomes (Fig. 1, A and B, and fig. S1, A and B) (*3*, *4*, *29*). The N-terminal leucine-rich Rix1 domain (residues 1 to 642) of PELP1 binds to WDR18, forming a stable tetrameric core with a stoichiometry of 2:2, to which one copy of TEX10 associates (fig. S1B). The highly conserved lysine-rich N-terminal tail of TEX10 mediates association of the rixosome with pre-ribosomes through interactions with the 28S rRNA and the ribosome assembly guanosine triphosphatase NOG2 (*3*). Compared to its yeast homolog (Ipi1), human TEX10 contains a large C-terminal extension (CTE) that further mediates association with NOG2, PELP1-WDR18, and binds to a small piece of the LAS1L nuclease (*3*). Structures of rixosome-bound pre-ribosomes from *S. cerevisiae* and *Chaetomium thermophilum* support that the rixosome scaffolding core has a conserved architecture and binds to pre-ribosomes near the ITS2 foot to mediate ITS2 processing (fig. S1, C to F) (*16*, *30*). Although present in the sample, the entire C-terminal half of PELP1 (residues 643 to 1130) and the enzymatic modules of the rixosome were not visible in the human pre-60S cryo-EM reconstructions presumably because the enzymatic modules are tethered to the rixosome core through flexible linkers (*3*). The dynamic nature of the rixosome has hindered our understanding of the overall assembly and architecture of this essential multienzyme complex.

Here, we reconstituted the human rixosome complex and determined how the enzymatic modules of the rixosome are modularly assembled into the complex. The C-terminal half of PELP1 is composed of an IDR enriched in proline and glutamic acid residues. Our structural and biochemical analyses show that this IDR supports binding to the regulatory subdomain of the AAA-ATPase MDN1, chaperones the histone octamer in vitro, and binds to and allosterically activates the SUMO protease SENP3.

## RESULTS

### Reconstitution of the human rixosome reveals its modular assembly

To understand the overall assembly and architecture of the rixosome, we defined the core rixosome interactome through reconstitution and affinity purification experiments. We reconstituted the exogenous rixosome (subunits PELP1, WDR18, TEX10, SENP3, LAS1L, and NOL9) through transient transfection in human embryonic kidney (HEK) 293FT suspension cells (Fig. 1, C and D). Using different truncations of PELP1 as affinity purification bait, we establish that the N-terminal Rix1 domain (residues 1 to 642) is necessary and sufficient for association with TEX10 and WDR18, in excellent agreement with previous experiments (*3*, *4*, *29*). The Rix1 domain also contributes to binding of LAS1L and NOL9 (Fig. 1, C and D). In our previous work we observed that in the absence of exogenous LAS1L and NOL9, the Rix1 domain was not sufficient for the stable association of TEX10 with PELP1-WDR18 under stringent purification conditions (*4*). Pre-60S structures support that LAS1L directly binds to TEX10 (*3*), suggesting that LAS1L provides additional structural support for TEX10 to be stably accommodated within the Rixosome in the absence of the PELP1 C-terminal region. Using a series of truncations of the C-terminal half of PELP1, we determined that a small region between residues 765 and 791 is required for association with SENP3. During our reconstitution experiments, we observed that the expression of several of the exogenous rixosome subunits was influenced by the varying expression levels of different PELP1 truncations, suggesting that rixosome members rely on one another for protein stability, in agreement with earlier studies (*31*–*33*).

**Fig. 2.**
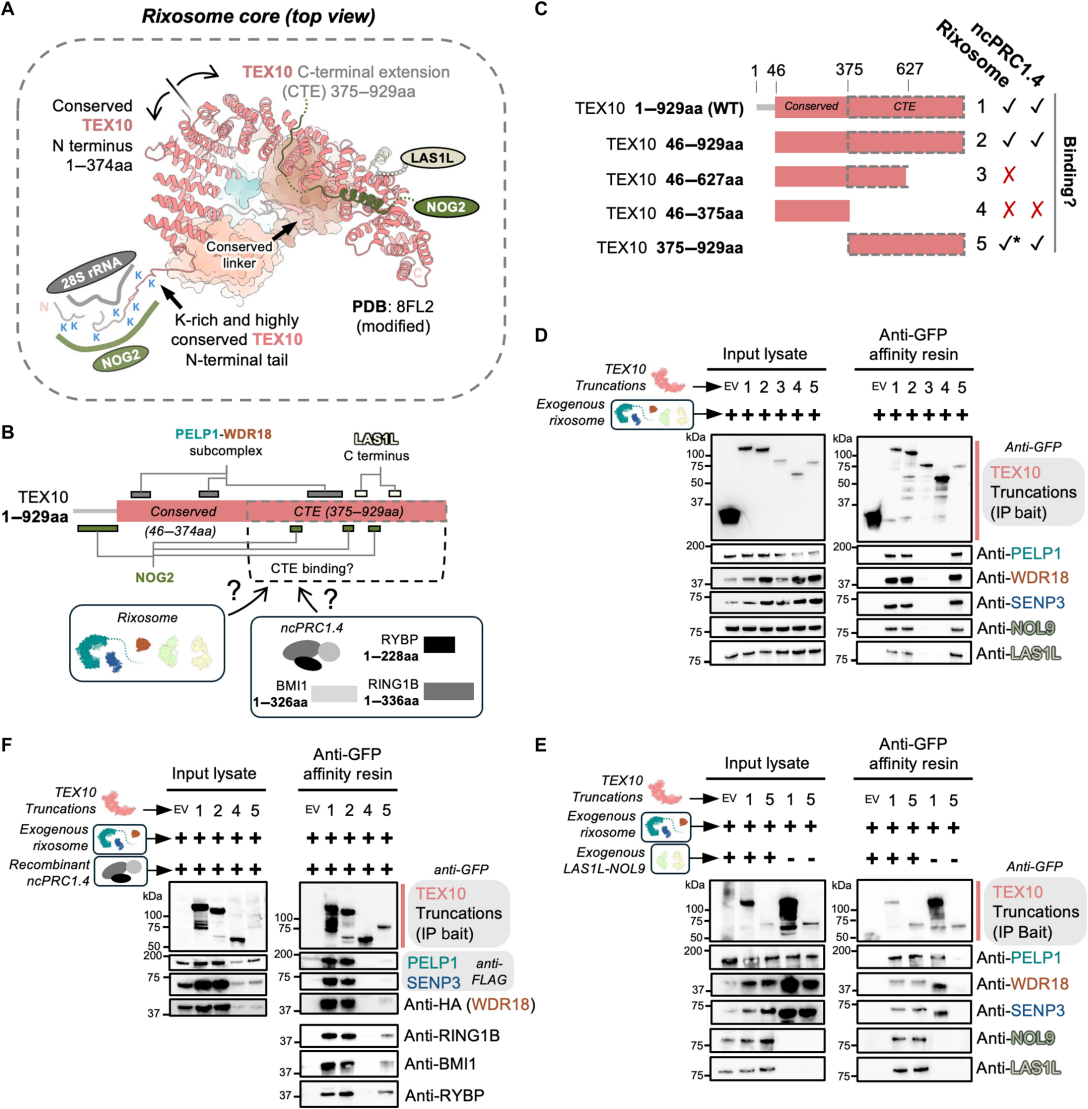
TEX10 supports the rixosome core. (**A**) View of the rixosome core focused on TEX10 (coordinates taken from [PDB ID:8FL2 (*3*)]. TEX10 contains a highly conserved N-terminal tail enriched in K residues that supports binding to the 28S rRNA and the assembly factor NOG2. The rest of TEX10 forms an α-solenoid structure that binds to the rixosome core as well as parts of NOG2 and LAS1L. (**B**) Cartoon schematic of TEX10 structural domains with known interfaces noted. Residues 1 to 374 are well conserved across eukaryotes, while the C-terminal extension (CTE) is only found in higher eukaryotes. (**C**) Schematic of N-terminal GFP tagged TEX10 variant/truncation constructs (1 to 5) used to affinity purify the rixosome in the co-IP experiments displayed in (D), (E), and (F). Checkmarks and X marks denote qualitative binding results for specific rixosome components in (D/E) or ncPRC1.4 complex in (F) to the TEX10 variant used for reconstitution and co-IP. * denotes a change in rixosome binding to the CTE when LAS1L-NOL9 are not exogenously coexpressed. (**D**) SDS-PAGE and Western blot using antibodies for the endogenous rixosome proteins qualitatively identified the presence or absence of rixosome proteins (endogenous and exogenous) upon co-IP from human cells of TEX10 variants in (C). (**E**) SDS-PAGE and Western blot using antibodies to qualitatively detect exogenous rixosome proteins upon co-IP of TEX10 WT versus CTE alone, and ± LAS1L-NOL9 coexpression. Data exhibited lack of rixosome binding to the TEX10 CTE alone when LAS1L-NOL9 is not coexpressed with the full rixosome. (**F**) SDS-PAGE and Western blot using antibodies to qualitatively detect exogenous rixosome proteins and recombinant full-length human ncPRC1.4 protein members upon TEX10 variant co-IP from human cells and incubation in vitro with recombinant ncPRC1.4.

In addition to PELP1, we also determined which regions of the TEX10 scaffold are critical for mediating the rixosome interactome. In contrast to PELP1, with the exception of a short lysine-rich N-terminal tail that is highly conserved, the majority of TEX10 folds into an ordered α-solenoid structure (Fig. 2, A and B) (*3*). Residues 1 to 374 of TEX10, which includes the loop linker segment that associates with the PELP1-WDR18 tetramer, are well conserved across eukaryotes, while the CTE (residues 375 to 929) is only found in higher organisms (Fig. 2, A and B). Using different truncations of TEX10 for co-IP, we observed that the structurally conserved N terminus of TEX10 does not stably bind the rixosome and that the N-terminal tail is dispensable for binding (Fig. 2, C and D). The TEX10 CTE alone is sufficient to bind the rixosome, underscoring the importance of this structural extension in higher organisms (Fig. 2, C and D). A cryo-EM structure of the rixosome bound pre-60S supports that LAS1L associates with the TEX10 CTE (*3*). To confirm this interaction, we repeated a subset of the TEX10 co-IP experiments with and without LAS1L-NOL9 (Fig. 2E). In the absence of LAS1L-NOL9, we do not observe stable binding of PELP1, WDR18, and SENP3 to the TEX10 CTE, supporting that this LAS1L-TEX10 interface further promotes stable rixosome assembly (Fig. 2E). Despite experimental structure-informed truncation of the TEX10 solenoid, protein truncations of TEX10 severely affect expression levels (Fig. 2, D and E), suggesting that the full TEX10 solenoid structure is needed for protein stability and full rixosome assembly.

Beyond the core rixosome components, TEX10 supports binding to NOG2 on the pre-60S ribosome (*3*) (largely via the TEX10 CTE) and PRC1 complexes via the RING1B subunit (*1*) (Fig. 2F). To determine which regions of TEX10 mediate binding to PRC1, we affinity purified the exogenous rixosome (PELP1, TEX10, WDR18, and SENP3) harboring different TEX10 truncations as bait (Fig. 2F), and then incubated these rixosome complexes with recombinantly purified human noncanonical (nc) PRC1.4 complex (RING1B-BMI1-RYBP). We observed that the TEX10 CTE is required for recombinant ncPRC1.4 binding (Fig. 2F). Together, these results support the TEX10 CTE being required for rixosome formation and suggest that the CTE plays a pivotal role in recruiting the rixosome to the pre-60S ribosome via NOG2 or PRC1 via RING1B.

### PELP1’s IDR mediates a modular interaction network with MDN1, histones, and SENP3

**Fig. 3.**
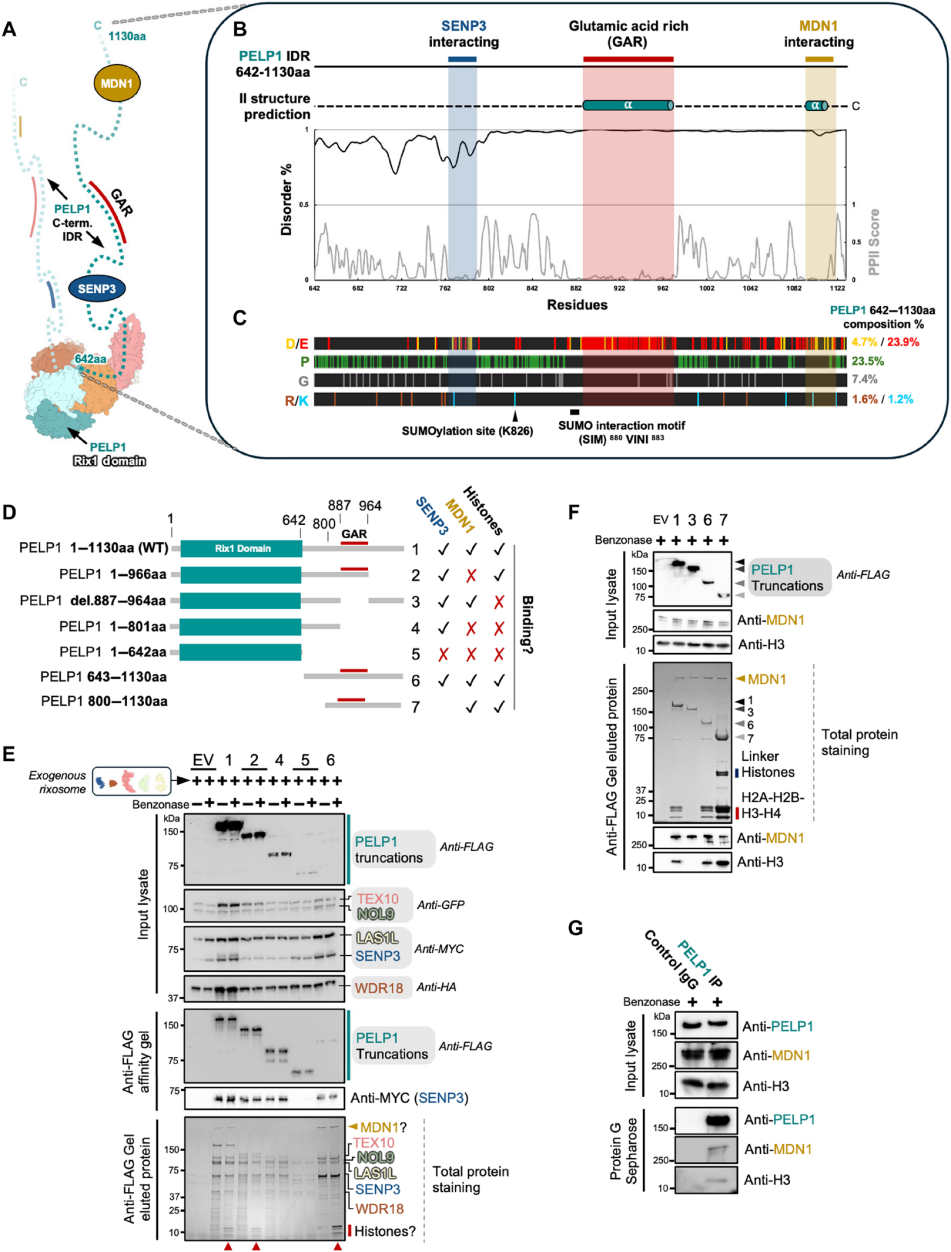
PELP1’s C-terminal IDR mediates a modular interaction network. (**A**) Human rixosome cartoon indicating both copies of PELP1’s C-terminal IDR, with specific areas of interest labeled. (**B**) Sequence and structural characteristics of the PELP1 C-terminal IDR (residues 642 to 1130). AlphaFold3 secondary (II) structure predictions, disorder propensity (%), and polyproline secondary structure propensity (PPII score) are displayed per residue of the PELP1 IDR. (**C**) Amino acid distribution plots for negative [D (red) and E (yellow)], positive [R (brown) and K (blue)], glycine (G, gray), and proline (P, green) residues. (**D**) Schematic of C-terminal FLAG-tagged PELP1 variant/truncation constructs (1 to 7) used in the co-IP experiment displayed in (E) and (F). Checkmarks and X marks denote qualitative binding results for SENP3, MDN1, or histones to the PELP1 variant used for reconstitution and co-IP. (**E**) SDS-PAGE and Western blot using antibodies for the exogenous rixosome proteins upon co-IP from human cells of PELP1 variants in (D) + or − a nonspecific nuclease in the lysis buffer. These isolated rixosome complexes were natively eluted off the anti-FLAG affinity gel and analyzed by SDS-PAGE and total protein staining to qualitatively identify endogenous interactors copurifying with the human rixosome. Red arrows at bottom denote sample lanes with histone copurification. (**F**) SDS-PAGE and Western blot using specific antibodies for the endogenous protein targets copurifying with PELP1/rixosome complexes upon co-IP from human cells in (E) + a nonspecific nuclease in the lysis buffer. MDN1 qualitative binding was confirmed by Western blot. Nucleosome core histones (H2A-H2B-H3-H4) qualitative binding was determined to be specific to the PELP1 GAR and confirmed by Western blot to H3. (**G**) SDS-PAGE and Western blot from an endogenous PELP1 co-IP from HEK293FT cells + a nonspecific nuclease in the lysis buffer using PELP1-specific antibody. Endogenous MDN1 and H3 were detected by Western blot.

To gain insight into the structure and function of the C-terminal half of PELP1, we used modeling tools and performed rixosome interactome assays, revealing that beyond SENP3, the PELP1 C terminus also binds to MDN1 and histones. Residues 642 to 1130 of PELP1 lack a defined structure and are predicted to be an IDR (Fig. 3, A to C). IDRs are a very common feature in proteins that are involved in the regulation of ribosome assembly, chromatin, and transcription and are known to facilitate the organization of dynamic interaction networks (*34*, *35*). IDRs are difficult to study by conventional approaches, but many IDRs include critical regions which help regulate various biological activities. IDRs have a high degree of conformational flexibility and often share common sequence characteristics such as low sequence complexity, high content of charged residues, repeats of polar residues, and low hydrophobicity (*36*). Moreover, many IDRs have a modular organization with local sequence bias (*37*). These unique sequence features of IDRs enable them to support dynamic multivalent interactions through a variety of mechanisms (*36*). Some of these interactions are formed through specific sequences that can become ordered upon binding an interaction partner while other interactions are transient non–sequence-specific associations that can drive processes like condensate formation.

We hypothesized that the IDR of PELP1 contains distinct modules important for mediating multivalent protein interaction networks involved in ribosome assembly and heterochromatin maintenance. The PELP1 IDR includes several regions that are enriched in prolines, a common feature of IDRs (*38*). Interspersed within the proline-rich regions are three distinct segments including the SENP3 binding region (residues 765 to 791) and two weakly predicted α-helix segments (Fig. 3, B and C, and fig. S2). The first α-helix is heavily enriched in glutamic acid residues and is known as the glutamic acid–rich (GAR) region (*39*). The second predicted α-helix lies at the end of the C terminus (Fig. 3, B and C). The IDR of PELP1 also contains a known SUMOylation site at position K826 (*24*) and a SUMO-interacting motif (SIM) in residues 880 to 883 (Fig. 3C) just before the GAR (*10*).

To establish the interaction network of the PELP1 IDR, we transfected cells with plasmids encoding the exogenous rixosome containing various FLAG-tagged truncations of the PELP1 IDR (Fig. 3D). We lysed the cells in both the presence and absence of a nonspecific nuclease, carried out affinity purification using anti-FLAG resin, and then used both Western blotting and total protein staining to determine what copurifies with the different PELP1 IDR truncations (Fig. 3E). In addition to the bands corresponding to the exogenous rixosome components, we observed a prominent band with a high molecular weight in all the pull downs, which included the very C terminus of PELP1. We observed several prominent lower molecular weight bands in all lanes, which included the GAR region and were lysed in the presence of a nuclease (Fig. 3E). The identity of these bands was confirmed by mass spectrometry and Western blotting with specific antibodies (Fig. 3F). The upper molecular weight band was identified as MDN1, an AAA-ATPase critical for ribosome assembly and a known interaction partner of the rixosome (*24*). The lower molecular weight bands were identified as the four core histones of the nucleosome (H2A-H2B-H3-H4). We also observed linker histones by total protein staining on an SDS–polyacrylamide gel electrophoresis (PAGE) gel (Fig. 3F). To confirm that these interactions were not an artifact of overexpression of the exogenous rixosome, we performed co-IPs in nontransfected HEK293FT cells using a PELP1-specific antibody and detected binding of both MDN1 and histone H3 to endogenous PELP1 (Fig. 3G). This work supports that the C terminus of PELP1 mediates association with MDN1 while the GAR region mediates association with histones. In the following sections, we further explore the structure and function of the PELP1 IDR and its specific interaction networks with MDN1, histones, and SENP3.

### The PELP1 IDR associates with the regulatory D2H2α insert of the AAA-ATPase MDN1

**Fig. 4.**
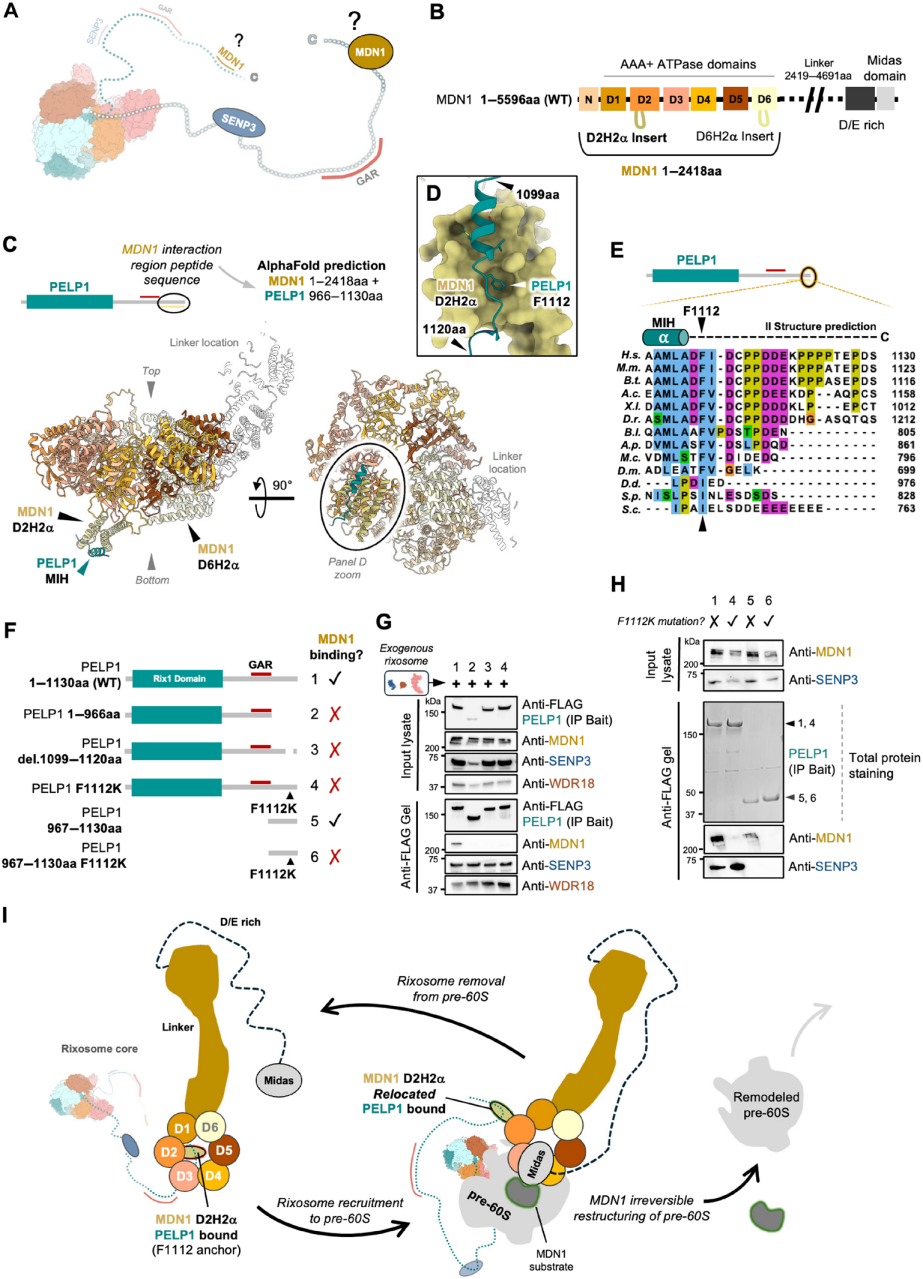
The PELP1 IDR associates with the regulatory D2H2α insert of the AAA-ATPase MDN1. (**A**) Cartoon of the human rixosome highlighting the specific PELP1 IDR-MDN1 interaction region of interest. (**B**) Schematic of human AAA-ATPase MDN1 noting major protein domains. The six N-terminal AAA-ATPase domains are specifically noted as D1-D6. Also see fig. S3. (**C**) AlphaFold3 structural prediction of the entire human MDN1 AAA-ATPase domain bound to the experimentally determined region of the PELP1 IDR. Exact protein residues used for the prediction are noted in the panel. AlphaFold3 predicts a small helix of PELP1 (MDN1 interacting helix, MIH) binds to the D2H2α insert of MDN1. Also see fig. S4. (**D**) Zoom from (C) of predicted MDN1-PELP1 binding interface with PELP1 F1112 forming an aromatic anchor into a pocket of the MDN1 D2H2a. Also see fig. S3. (**E**) Multiple sequence alignment of PELP1 MIH region responsible for binding MDN1 D2H2a. Species abbreviations: *Homo sapiens* (H.s.), *Mus musculus* (M.m.), *Bos taurus* (B.t.), *Aquila chrysaetos* (A.c.), *Xenopus laevis* (X.l.), *Danio rerio* (D.r.), *Branchiostoma lanceolatum* (B.l.), *Acanthaster planci* (A.p.), *Mytilus coruscus* (M.c.), *Drosophila melanogaster* (D.m.), *Dictyostelium discoideum* (D.d.), *Schizosaccharomyces pombe* (S.p.), *Saccharomyces cerevisiae* (S.c.). (**F**) Schematic of C-terminal FLAG tagged PELP1 truncations, deletions, and point mutants (1 to 6) used for co-IPs to assay endogenous MDN1 binding from human cells in (G) and (H). (**G**) SDS-PAGE and Western blot using antibodies for the endogenous MDN1 protein and exogenous rixosome components upon co-IP of PELP1 variants in (F). All PELP1 MIH mutants failed to bind qualitatively to MDN1, supporting the predicted structural model of PELP1 MIH binding to MDN1 D2H2a in (C). (**H**) SDS-PAGE, Western blot, and total protein stain of FLAG PELP1 C terminus (residues 967 to 1130) alone copurifying MDN1 from cells. (**I**) A proposed mechanism for MDN1 regulation by PELP1/the rixosome in large ribosomal subunit maturation.

To probe the PELP1 and MDN1 interface, we used experimentally validated structure predictions to establish that a conserved motif within the PELP1 C terminus is required for MDN1 binding. MDN1 is a very large protein composed of over 5000 residues that contains six concatenated AAA domains (known as D1-D6), followed by a linker region, a D/E-rich region, and a C-terminal metal ion–dependent adhesion site (MIDAS) domain that mediates the release of assembly factors from pre-ribosomes (Fig. 4, A and B, and fig. S3) (*40*). In addition to its role in ribosome assembly, MDN1 has also been linked to rixosome function at heterochromatin, as MDN1 ChIP-Seq experiments revealed that MDN1 localizes to transcription start sites where there is a high occupancy of polycomb (*1*). We used AlphaFold3 to generate a model of the PELP1-MDN1 interface (Fig. 4, C and D, and fig. S4) (*41*). AlphaFold3 predicts a high-confidence interaction between the predicted C-terminal α-helix (herein referred to as MIH for MDN1-interacting helix) from PELP1 and an insertion subdomain from MDN1 (Fig. 4, C and D, and fig. S4). This insertion subdomain is known as D2H2α because it is found following α-helix2 within the second AAA domain (D2) of MDN1.

Previous in vitro binding assays with the *S. cerevisiae* homologs established that the C terminus of PELP1 (Rix1) mediates binding of the rixosome to the D2H2α insert of MDN1 (Rea1) and deletion of either of these regions is lethal (*42*). Low-resolution cryo-EM structures of pre-60S ribosomes from *S. cerevisiae* revealed density attributed to the D2H2α insert of Rea1 in close proximity to the yeast rixosome core (fig. S3A) (*42*). This was followed by higher resolution cryo-EM reconstructions that visualized the rixosome core components and Rea1, better establishing their structural location on the pre-60S, but failing to visualize a direct Rea1-rixosome interaction (fig. S3B) (*30*). AlphaFold3 models predict an interaction between the end of the Rix1 C terminus and the D2H2α insert from Rea1 in both *S. cerevisiae* and *S. pombe* (fig. S4, H to I). Collectively, this supports that the PELP1-MDN1 interaction is conserved; however, there is poor sequence conservation within the PELP1 C terminus between yeast and humans (Fig. 4E). Multiple sequence alignments of the PELP1 C terminus revealed a conserved polar motif at the end of MIH that is well conserved in metazoans (Fig. 4E). The AlphaFold3 model places F1112 from the MIH into a hydrophobic pocket from the MDN1 D2H2α insert (Fig. 4D, fig. S4, B, C, and F), while this residue is replaced by an isoleucine in lower organisms (fig. S4, H to I). To establish the importance of F1112 in mediating the PELP1-MDN1 interaction in humans, we mutated this PELP1 residue to a lysine. Both the F1112K mutation and truncation of the PELP1 MIH (residues 1099 to 1120) abolish binding of endogenous MDN1 to the exogenous rixosome (Fig. 4, F and G). The F1112K mutation does not disrupt recruitment of the other rixosome core members or affect the stability of the rixosome core members. Further, the PELP1 C terminus alone with an intact MIH is sufficient to copurify endogenous MDN1 from cells supporting this interaction is independent from other members of the rixosome and regions of the PELP1 IDR (Fig. 4, F and H). This work supports the hypothesis that the PELP1 MIH fosters an interaction network that anchors MDN1 to the rixosome complex.

The specific interaction of PELP1 with the D2H2α insert of MDN1 suggests that PELP1 could play an important role in mediating the ATPase activity of MDN1. Cryo-EM structures of isolated MDN1 homologs from *S. pombe* and *S. cerevisiae* position the D2H2α insert at the base of the hexameric AAA-ATPase ring, where it appears to plug the AAA ring and inhibit ATPase activity (*43*, *44*) (figs. S3 and S4, D and E). Truncation of the D2H2α insert leads to a 15-fold increase in ATP hydrolysis activity in vitro (*43*). While not well ordered in any ribosome bound structure obtained thus far, D2H2α appears to move out of the AAA-ATPase ring when bound to the ribosome (fig. S3) (*30*, *42*). Recent cryo-EM structures of Rea1^ΔAAAH2α^ on its own support that the structure of this truncation resembles the active ribosome bound state of Rea1 (*45*). We speculate that PELP1 may help regulate the ATPase activity of MDN1 by facilitating movement of the D2H2α insert once the rixosome associates with pre-ribosomes (Fig. 4I). During ribosome assembly, ATP hydrolysis by MDN1 is critical for generating the force needed to trigger assembly factor removal from pre-ribosomes. It remains unclear if MDN1 plays a similar role to facilitate remodeling of complexes at heterochromatin.

### The conserved GAR region of PELP1 chaperones the histone octamer in vitro

**Fig. 5.**
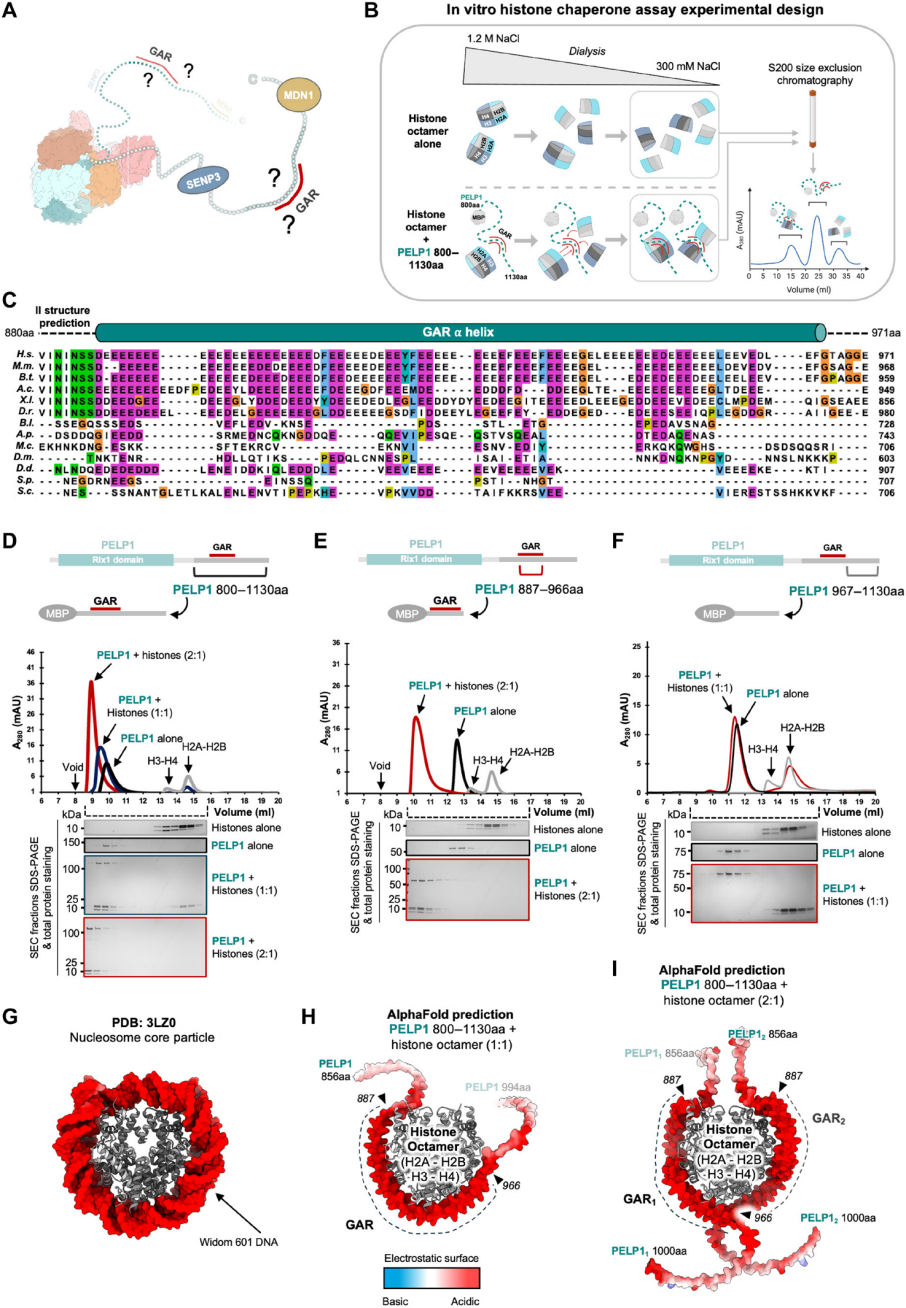
The GAR region of PELP1 chaperones the histone octamer in vitro. (**A**) Cartoon of the human rixosome highlighting the GAR region. (**B**) Cartoon of the in vitro histone chaperoning assay illustrating potential IDR histone interactions that could form upon incubating PELP1 with H2A-H2B dimers and H3-H4 tetramers. Created in BioRender. Gordon, J. (2025) https://BioRender.com/x62f662. (**C**) Multiple sequence alignment of PELP1 GAR. Highly conserved stretches of glutamic acid residues are highlighted in magenta. Species abbreviations are the same as Fig. 4E. (**D** to **F**) Histone chaperone assay with PELP1 IDR region 800 to 1130aa (D), PELP1 IDR region 887 to 966aa (E), and PELP1 IDR region 967 to 1140aa (negative control) (F). Postdialysis histone octamer and MBP-PELP1 variant mixed samples were subject to S200 gel filtration chromatography to assess histone binding to PELP1 GAR. Mixed samples at 1:1 (blue curve) and 2:1 (red curve) ratios exhibited migration shifts compared to unmixed controls, indicating stable binding. SDS-PAGE of peak fractions also indicate the presence of bound complexes. Histone octamer alone control sample exhibits two peaks corresponding to dissociated histone H2A-H2B dimers and H3-H4 tetramers. (**G**) Structure of the nucleosome core particle [PDB ID: 3LZ0 (*99*)]. DNA is colored on the basis of electrostatic surface. (**H**) AlphaFold3 structural prediction of the human histone octamer bound to one copy of the PELP1 GAR, 1:1 stoichiometry. Exact protein residues used for the prediction are noted in the panel (full-length human H2A_2_-H2B_2_-H3_2_-H4_2_ used for octamer prediction). PELP1 GAR is predicted to be helical when bound to histones and is depicted as electrostatic surface representation. Also see fig. S5. (**I**) AlphaFold3 structural prediction of the human histone octamer bound to two copies of the PELP1 GAR, 2:1 stoichiometry. Exact protein residues used for the prediction are noted in the panel and same as (H). Also see fig. S6.

Our PELP1 IDR truncations revealed that the GAR region supports binding to the nucleosome core histones (Figs. 3, E to G, and 5A) but the importance of this interaction was unknown. This led us to perform in vitro assays that established PELP1 can function as a chaperone for the histone octamer (Fig. 5B). The GAR region of PELP1 is heavily enriched in glutamic acid residues and to a lesser extent, aspartic acid residues. AlphaFold3 structural predictions suggest that the GAR region has the propensity to form a long α-helix (Fig. 5C), in agreement with earlier work demonstrating that poly-glutamic acid forms α-helical conformations under certain conditions (*46*). While the acidic nature of the PELP1 IDR is conserved across eukaryotes, the large clusters of D/E tracts are only found in higher organisms (Fig. 5C and fig. S2). Clusters of D/E tracts are a characteristic feature of many IDRs found among nucleolar proteins and recent work suggests that these D/E tracts facilitate phase separation in the nucleolus (*47*). In addition to supporting phase separation, D/E tracts have also been associated with histone binding due to their acidic nature, which can mimic DNA (*48*, *49*). Several other members of the rixosome also contain D/E-rich regions including LAS1L, MDN1, and SENP3 (fig. S5). However, the association of the rixosome with histones is mediated specifically by the PELP1 GAR as we do not observe histone binding when the GAR is missing, but the D/E tracts within LAS1L, SENP3, and MDN1 are present (Fig. 3, D to F, and fig. S5, C and D). Moreover, previous studies have identified histones as interaction partners of PELP1 in several mammalian cell lines (*39*, *50*–*54*).

We hypothesized that the GAR region of PELP1 may function as a histone chaperone because it shares characteristic features with histone chaperones (*48*, *49*) and poly-glutamic acid can assemble histones in vitro under physiological conditions (*55*). We observed what appeared to be stoichiometric levels of the nucleosome core histones copurifying with the PELP1 GAR by SDS-PAGE (Fig. 3F). This suggests that PELP1 is not specifically associating with the H3-H4 tetramer or the H2A-H2B dimer and could be chaperoning the intact histone octamer. To test this hypothesis, we used an established in vitro histone chaperone assay (Fig. 5B) (*56*). We assembled human histone octamers using recombinant purified H2A/H2B dimers and H3/H4 tetramers in 2:1 stoichiometry. We then incubated the histone octamers in high salt in the presence or absence of recombinantly purified maltose binding protein (MBP)–PELP1 GAR constructs. The salt was lowered by dialysis overnight and then size exclusion chromatography (SEC) was used to detect the presence of histone-chaperone complexes. If PELP1 can chaperone the octamer, we expected to observe a higher molecular weight peak corresponding to a complex formed between PELP1 and the histone octamer. Alternatively, if PELP1 cannot chaperone the octamer, we should observe separate peaks for the H2A-H2B dimer and the H3-H4 tetramer as they do not stably associate in low-salt conditions in the absence of DNA or a chaperone. We used different MBP fusion constructs of recombinant PELP1 in the histone chaperone assays including MBP-PELP1^800–1130^ and MBP-PELP1^887–966^, which include the entire GAR region (Fig. 5, D and E). The MBP fusion was needed because the very acidic nature of the GAR makes it difficult to stably purify on its own. We observed the formation of a histone octamer-chaperone complex with GAR-containing PELP1 constructs but not with MBP-PELP1^967–1130^, the PELP1 region immediately following the GAR (Fig. 5F). All four histones in the chaperone complexes were present in equal stoichiometries, further supporting association of the PELP1 GAR with intact histone octamers. When we used a ratio of 1:1 PELP1 to histone octamer, not all the histones were incorporated into the octamer-chaperone complex, hinting that the PELP1-octamer stoichiometry may be higher. When we increased the ratio to 2:1 PELP1 to histone octamer, we observed a single peak and no free histones. Collectively, these data establish that the GAR region of PELP1 can function as a chaperone of the histone octamer in vitro under physiological conditions and that two copies of the PELP1 GAR region stably associate with the histone octamer.

Most histone chaperones have specificity for either the H2A-H2B dimer or the H3-H4 tetramer (*48*, *49*), making PELP1 somewhat unique in being able to chaperone the histone octamer. Recent work has identified APLF (aprataxin and PNK-like factor) as another protein that can chaperone the histone octamer with the same stoichiometry as PELP1 (2 APLF: 1 histone octamer) (*56*). Like PELP1, the chaperone function of APLF is driven by a flexible acidic domain enriched in D/E residues. A distinguishing feature of the APLF acidic domain from other histone chaperones is the presence of aromatic anchor residues disbursed throughout the domain (*56*). PELP1 also contains several aromatic residues within its D/E tracts further, suggesting that these aromatic residues may be a unique feature of chaperones of the histone octamer. Solution nuclear magnetic resonance studies revealed that the APLF acidic domain envelopes the histone octamer and promotes a new mode of nucleosome assembly in vitro (*56*). To investigate if PELP1 might associate with the histone octamer in a similar fashion, we used AlphaFold3 to predict structural models of the PELP1 GAR-histone octamer chaperoning complex (Fig. 5, H and I, and fig. S6). These predicted structures depict the GAR helix wrapped around the histone octamer following a similar path to the DNA in the nucleosome core particle (Fig. 5G). This association is likely mediated by the negative surface charge of the GAR helix. Additional work will be needed to determine the relevance of this chaperone function in vivo. Given the presence of the rixosome at heterochromatin, it seems highly plausible that this chaperone function may influence various roles of the rixosome in heterochromatic gene silencing.

### Crystal structure of PELP1 bound to the SUMO protease domain of SENP3

**Fig. 6.**
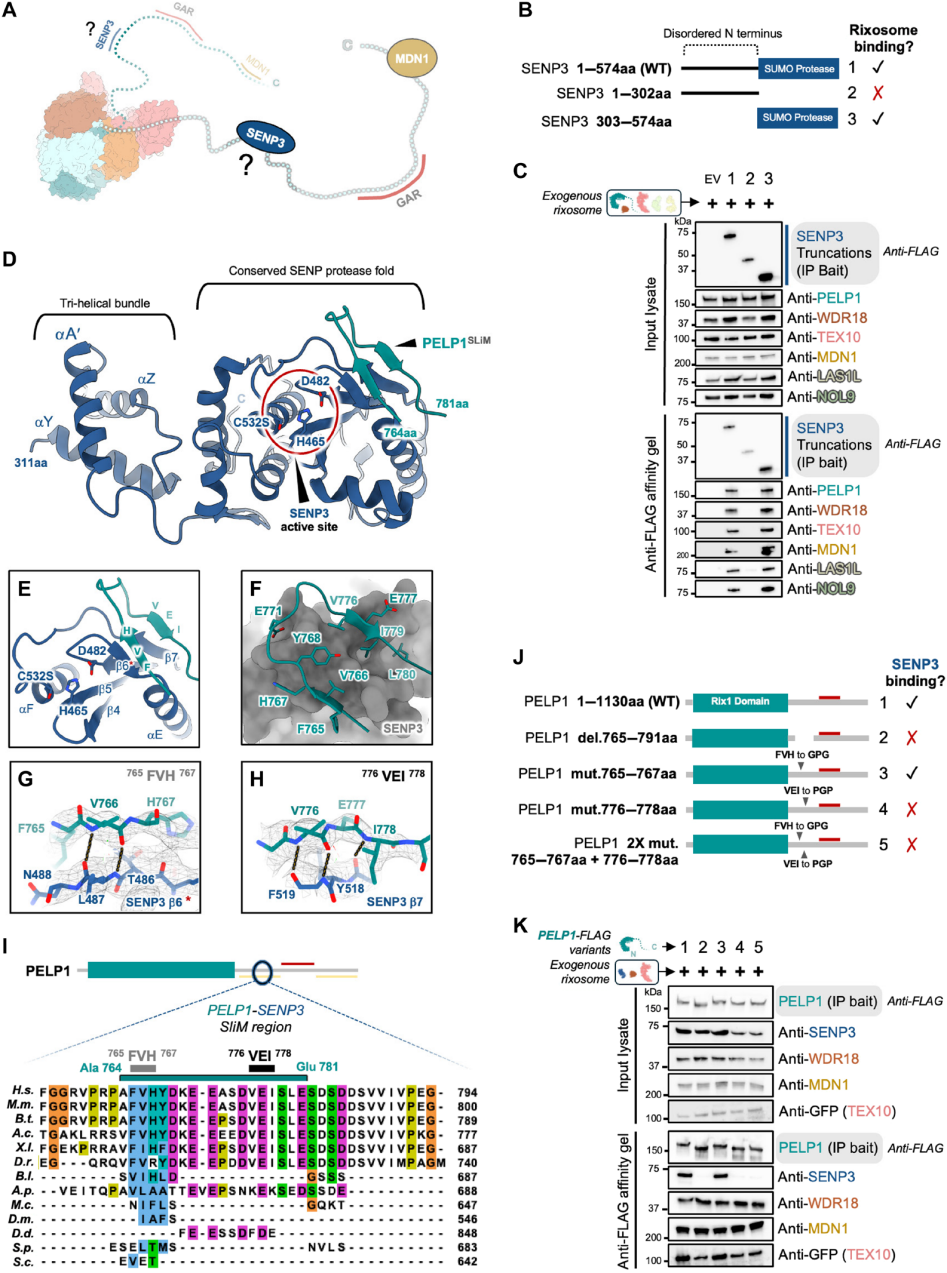
Structural basis for SENP3 SUMO protease incorporation within the rixosome. (**A**) Cartoon of the human rixosome highlighting the SENP3-interacting region. (**B**) N-terminal FLAG-tagged SENP3 truncations (1 to 3) used in the co-IP experiment displayed in (C). Checkmarks and X marks denote qualitative binding results for specific rixosome components in (C) to the SENP3 truncation used for co-IP. (**C**) SDS-PAGE and Western blot using antibodies for the endogenous rixosome proteins identified the presence or absence of rixosome proteins (endogenous and exogenous) upon co-IP with SENP3 truncations. (**D**) X-ray crystal structure of the SENP3 protease domain (311 to 574aa, dark blue) C532S mutant bound to the PELP1^SLiM^ peptide (764 to 781aa, teal) at 2.93 Å resolution. SENP3 active site with catalytic triad residues is circled in red. (**E**) PELP1^SLiM^ residues 765 to 767 (FVH motif) and 776 to 778 (VEI motif) form antiparallel β-strand interactions with SENP3 at β6* and β7, respectively. (**F**) The PELP1^SLiM^ binding interface with the SENP3 protease domain surface. (**G**) The PELP1 FVH motif secondary structure backbone interactions with SENP3 β6*. Putative backbone hydrogen bonding interactions are illustrated by dashed lines. Experimental electron density is displayed as a composite omit map contoured at σ = 0.152 e/Å^**3**^. (**H**) The PELP1 VEI motif secondary structure backbone interactions with SENP3 β7. Putative backbone hydrogen bonding interactions are illustrated by dashed lines with the experimental density shown as in (G). (**I**) Multiple sequence alignment of PELP1^SLiM^ and flanking regions. Species abbreviations are the same as Fig. 4E. (**J**) Schematic of C-terminal FLAG-tagged PELP1 mutant constructs (1 to 5) used in the co-IP experiment displayed in (K). Checkmarks and X marks denote qualitative binding results for SENP3 (K) to the PELP1 mutant. (**K**) SDS-PAGE and Western blot identified the presence or absence of SENP3 and other rixosome proteins upon co-IP from human cells of PELP1 mutant constructs in (J).

Last, we investigated the interaction between the PELP1 IDR and SENP3 through structural and functional approaches (Fig. 6A). SENP3 belongs to the Ulp/sentrin-specific protease family, whose members are characterized by a conserved C-terminal SUMO-specific protease domain and distinguished by their unique N-terminal regions, which are important for localization and substrate binding (*22*, *57*). To determine which regions of SENP3 are required for rixosome association, we created SENP3 truncations and performed affinity purifications with the exogenous rixosome using FLAG-tagged SENP3 as bait (Fig. 6, B and C). Constructs that included the SUMO protease domain were able to bind the rixosome, while the N-terminal domain was not sufficient for rixosome binding. Even though SENP3 only associates with a small region of the PELP1 IDR (Fig. 1, C and D), SENP3’s SUMO protease domain (residues 303 to 574) is sufficient to stably isolate the rixosome complex (Fig. 6, B and C).

To understand the structural basis for SENP3 and PELP1 association, we determined the x-ray crystal structure of the SENP3 SUMO protease domain bound to a fragment of the PELP1 IDR (Table 1). We used multiple sequence alignments and structure predictions to create a series of SENP3 catalytic domain constructs for protein expression in *Escherichia coli* and crystallization trials with two different SENP3-interacting peptides from PELP1 [short (s) PELP1, residues 764 to 781; or long (l) PELP1, residues 764 to 792]. Because we could not obtain suitable crystals with SENP3 on its own, we created a fixed-arm MBP-SENP3 catalytic domain fusion construct (MBP-SENP3^311–574^) containing a mutation at the active site cysteine (C532S). We obtained crystals of MBP-SENP3^311–574 C532S^ grown in the presence of excess sPELP1 peptide. The structure was solved by molecular replacement using an available structure of MBP (*58*) and truncated AlphaFold3 models of SENP3 (Fig. 6, D to F, and fig. S7). Clear, unbiased electron density was visible for the entire sPELP1 peptide, which was not included in the molecular replacement search models (Fig. 6, G and H, and fig. S7, A to C).

**Table 1.** X-ray crystallography data collection and atomic model refinement statistics.

	MBP(wt)-SENP3 + sPELP1 peptide*
PDB ID code	9ME8
**Data collection**	
Space group	*P*3_2_21
Unit cell dimensions	
*a*, *b*, *c* (Å)	140.97, 140.97, 119.63
α, β, γ (°)	90, 90, 120
Resolution range† (Å)	34.71–2.93 (2.98–2.93)
*R*_merge_†	0.31 (3.67)
*R*_pim_†	0.09 (1.05)
CC_1/2_†	0.99 (0.35)
Mean I/σI†	8.6 (1.1)
Completeness† (%)	96.6 (100)
Redundancy†	11.4 (12.1)
**Refinement**	
Resolution† (Å)	2.93 (3.02–2.93)
No. of reflections	28,978
*R*_work_/*R*_free_ (%)	20.19/23.22
No. of atoms	
Protein	4951
sPELP1 peptide	147
Water	5
*B* factors (Å^2^)	
Protein	72.69
sPELP1 peptide	70.73
Water	60.25
RMS deviations	
Bond lengths (Å)	0.006
Bond angles (°)	0.752

*A single crystal was used for data collection.

†Values in parenthesis are for the last resolution shell.

The overall structure of the SENP3^C532S^ catalytic domain closely resembles the structures of other ULP/SENP family members, with one notable exception at the N terminus of the protease domain (Fig. 6D and figs. S7E and S8). SENP3 and SENP5 share a similar predicted tri-helical bundle directly preceding the catalytic domain that is not found in the other human SENPs (fig. S7E). This tri-helical bundle lies adjacent to the first α-helix from the protease domain where it appears to play a structurally supportive role, protecting a hydrophobic patch within the protease domain. The tri-helical bundle also adopts a unique fold as a search of the Protein Data Bank (PDB) or human AlphaFold database with the DALI server (*59*) did not return any notable hits aside from the predicted structures of SENP3 and SENP5. The rest of the SENP3 catalytic domain adopts the characteristic fold of cysteine proteases, including a central mixed β-sheet surrounded by five α-helices (Fig. 6, D and E; and figs. S7, D and E, and S8). The active site pocket of SENP family members is formed by a conserved H-D-C catalytic triad (H465, D482, and C532 of SENP3), which is critical for mediating peptide hydrolysis. While a SUMO substrate is not present in our structure, the conserved SUMO binding interface can be readily identified by superimposing prior SENP-SUMO structures and modeling with AlphFold3 (fig. S9). AlphaFold3 yields high-confidence predictions of a SENP3-PELP1-SUMO2 complex positioning SUMO2 in the SENP3 active site in excellent agreement with experimental SENP-SUMO structures. These predictions also accurately place the c-α backbone of the sPELP1 IDR peptide bound to SENP3 that we determined in our x-ray structure (fig. S9).

The PELP1 peptide binds across a positively charged surface of SENP3 adjacent to the mixed β-sheet near the active site (fig. S7A). While predicted to be largely disordered, two small segments of the PELP1 IDR form β-strands upon binding to SENP3. Residues 776 to 778 containing a well-conserved VEI motif from PELP1 form an antiparallel β-strand interface with the last β-strand (β7) from SENP3’s mixed β-sheet (Fig. 6, E to I). Residues 765 to 767 containing a conserved FVH motif also form an antiparallel β-strand with another β-strand from SENP3 (Fig. 6, E, F, G, and I). To probe the importance of these interfaces, we created a series of mutations within the PELP1 IDR in which we either deleted or mutated the VEI and FVH regions (Fig. 6J). Loss of the VEI motif prevents the association of PELP1 and SENP3, while loss of the FVH motif did not prevent binding (Fig. 6, J and K). These results are supported by the x-ray structure where the PELP1 VEI motif backbone can putatively form more H-bonds with SENP3 than the PELP1 FVH motif (Fig. 6, G and H). While the structure supports peptide electrostatics and other residues within the PELP1 IDR interacting with SENP3, the secondary structure interactions of PELP1 ^776^VEI^778^ with SENP3 β7 are essential for SENP3 incorporation into the rixosome.

Multiple sequence alignments of the PELP1 IDR and SENP3, along with AlphaFold3 predictions of the PELP1-SENP3 complex, support that this interface is evolutionarily well conserved. The PELP1 sequence that binds to SENP3 fits the classic description of a short linear motif (SLiM). SLiMs are commonly found in IDRs and are typically composed of 3 to 15 amino acids (aa) that become structured upon interaction with a partner protein (*35*, *60*). Our crystal structure and independent AlphaFold3 predictions confidently support that the PELP1^SLiM^ adopts a β-strand architecture upon binding SENP3 (fig. S10). Intriguingly, the PELP1^SLiM^ coappears evolutionarily with the appearance of SENP3 in vertebrates, and AlphaFold3 structure predictions support that the PELP1-SENP3 interface is well conserved across vertebrates (fig. S11).

### PELP1^SLiM^ allosterically activates SENP3

**Fig. 7.**
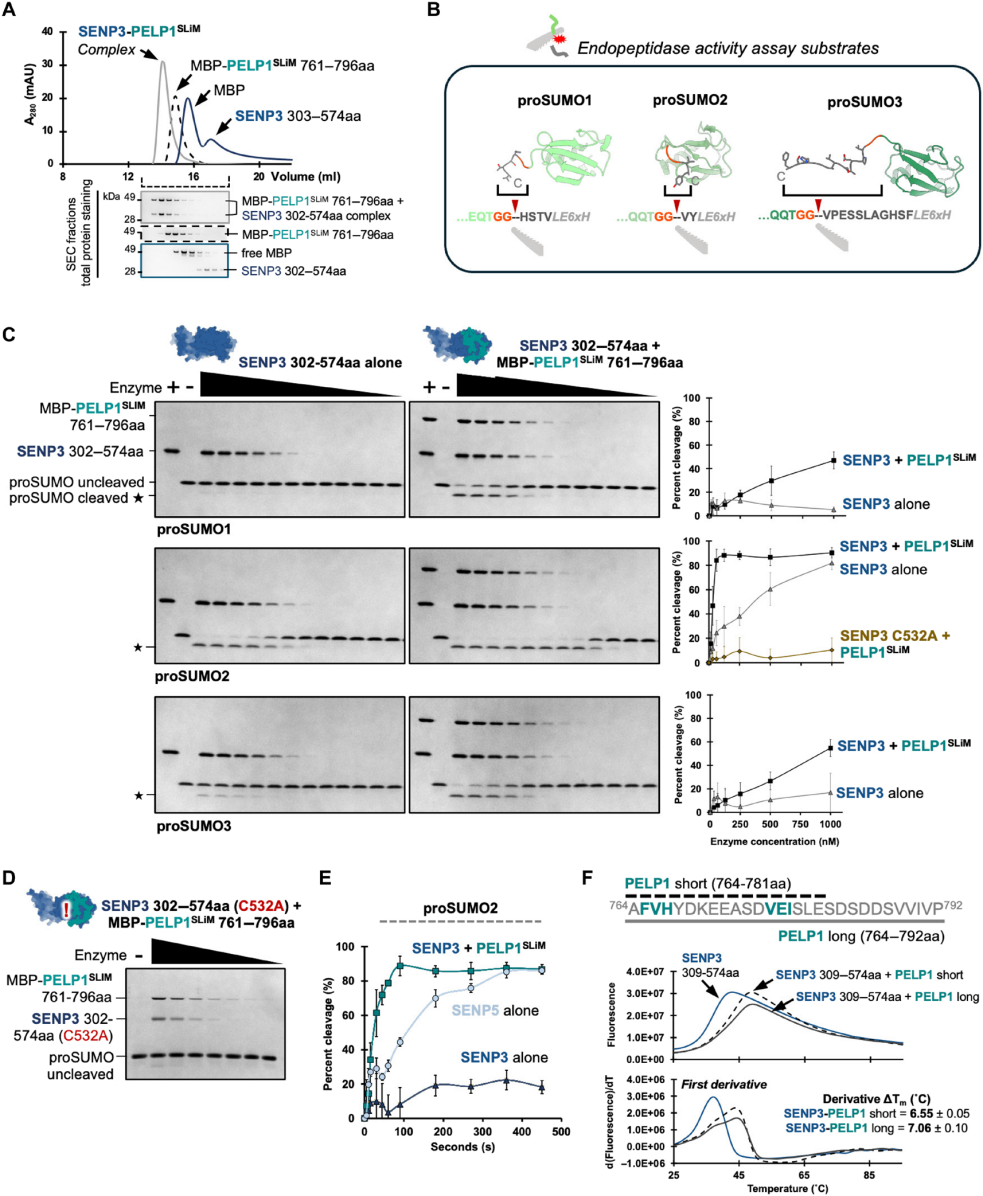
PELP1^SLiM^ allosterically activates SENP3. (**A**) Size exclusion chromatography (SEC) curves exhibiting the formation of a complex between SENP3 protease domain 302 to 574aa and PELP1^SLiM^-containing peptide 761 to 796aa. SDS-PAGE and total protein staining of SEC fractions are displayed below the *x* axis. (**B**) AlphaFold3 structural models of proSUMO substrates used in endopeptidase cleavage assays. Sequences of the proSUMO C-terminal tails (cleavage sites labeled with arrows) are shown below the structural models. (**C**) SDS-PAGE and total protein stain of in vitro SENP3 ± MBP-PELP1^SLiM^ endopeptidase activity assays against proSUMO1, −2, and −3 substrates. Decreasing concentrations (2500 to 0.12 nM) of SENP3 enzyme ± MBP-PELP1 761 to 796aa was incubated for 1 hour at 37°C with 5 μM proSUMO substrate. Cleaved proSUMO product is labeled with asterisks. Quantification curves representing percent (%) proSUMO endopeptidase cleavage correspond to the gel images and the enzyme concentration range was 1000 to 15.63 nM. SD was calculated from three independent experiments (*n* = 3). (**D**) SDS-PAGE and total protein stain of in vitro SENP3 (C532A) ± MBP-PELP1^SLiM^ endopeptidase activity assay, illustrating no activity. (**E**) Quantification curves representing percent (%) proSUMO2 endopeptidase cleavage by SENP protease domain + PELP1^SLiM^, SENP3 protease domain alone, or SENP5 protease domain alone during a time course (0 to 450 s). Enzyme concentration was kept constant at 1000 nM along with substrate concentration at 5 μM. Percent cleavage was calculated as in (C). SD was calculated from three independent assay samples (*n* = 3). Raw gel images are displayed in fig. S13A. (**F**) Differential scanning fluorimetry curves exhibiting the thermal stabilization of the SENP3 protease domain upon addition of short (“s,” amino acids 764 to 781) and long (“l,” amino acids 764 to 792) PELP1^SLIM^ peptides. Boltzmann and first derivative curves are shown on the top and bottom of the panel, respectively. Values for change in *T*_m_ is only shown for the first derivative.

Given that the PELP1^SLiM^ binds near the SENP3 active site, we hypothesized that PELP1 may play a role in regulating SENP3 activity. Previous attempts at measuring SENP3 activity in vitro have been challenging because of difficulties producing recombinant SENP3 (*61*). Compared to all the other SENPs, in vitro translated SENP3 shows the weakest activity against a variety of SUMO substrates, hinting that SENP3 may require an activating factor (*62*). To determine how the PELP1^SLiM^ influences SENP3 activity, we fused the PELP1^SLiM^ to MBP so that we could stably produce the recombinant SLiM in *E. coli.* To confirm that the MBP-PELP1^SLiM^ associates with the SENP3 protease domain, we used size exclusion chromatography (SEC). We observed a large shift, indicative of complex formation between the MBP-PELP1^SLiM^ and SENP3 protease domain that was not observed with mixed MBP and SENP3 protease domain (Fig. 7A).

To measure SENP3 activity, we used an established gel-based proSUMO cleavage assay (*61*), which revealed that PELP1 positively influences SENP3 activity. Many SENP/Ulp family members have dual functions and are involved in both SUMO maturation (endopeptidase function) and SUMO deconjugation (isopeptidase function) (*22*, *57*, *63*). There are three common proSUMO isoforms in humans, which have variable C-terminal tails adjacent to a di-glycine motif (Fig. 7B). SENP1, SENP2, SENP3, and SENP5 have all been shown to be important for cleaving these C-terminal tails and generating the mature forms of SUMO1, SUMO2, and SUMO3. We purified recombinant proSUMO isoforms and carried out in vitro endopeptidase assays. We titrated amounts of SENP3 protease domain alone or SENP3 in complex with MBP-PELP1^SLiM^ with a fixed amount of the proSUMO to assay for cleavage of the C-terminal tails. For SENP3 alone, we observed cleavage activity for proSUMO2, but very little activity towards proSUMO1 and proSUMO3 (Fig. 7C), somewhat consistent with previous studies demonstrating SENP3 has a preference for SUMO2 and SUMO3 (*10*, *23*, *26*, *62*, *64*). In contrast to SENP3 alone, when MBP-PELP1^SLiM^ was bound to SENP3 and included in the reaction, we observed activity against all three proSUMO isoforms and a striking increase in the activity for proSUMO2 (Fig. 7C). The catalytic mutant of SENP3 (C532A) displays no activity against proSUMO2 in the presence of MBP-PELP1^SLiM^ (Fig. 7D). To further investigate PELP1’s influence on SENP3 activity, we carried out time course experiments and compared SENP3 protease domain activity to that of SENP5 protease domain. SENP3 and SENP5 are the most closely related of the human SENPs in terms of sequence similarity and both play important roles in ribosome assembly (*23*). SENP5 is more active than SENP3 alone in our in vitro proSUMO2 endopeptidase assay, where we used a fixed amount of enzyme (1000 nM) incubated with 5 μM proSUMO2 substrate (Fig. 7E). However, the SENP3-PELP1^SLiM^ complex reaches completion significantly faster than SENP5 alone. These experiments demonstrate that PELP1 has a strong influence on the SENP3’s SUMO protease activity in vitro.

The PELP1^SLiM^ does not bind directly to the SENP3 active site; therefore, we propose that PELP1 is an allosteric regulator of SENP3. While the SUMO protease domain is well conserved across the six human SENPs both in terms of sequence and structure, one region that is poorly conserved is the final β-strand in the mixed β-sheet (β7 in all SENPs) (figs. S7E and S8). This is the β-strand upon which the PELP1^SLiM^ forms a critical interaction via the VEI motif. Compared to the other human SENPs, β7 from SENP3 is composed of residues with low propensity for β-strand formation. This weakened β7 likely explains the need for the PELP1^SLiM^ to stabilize the entire mixed β-sheet. To test this hypothesis, we used differential scanning fluorimetry to measure the stability of SENP3 protease domain in the presence and absence of the PELP1^SLiM^ (Fig. 7F). Compared to SENP3 alone, we observed an increase in the thermal stability with either of the PELP1^SLiM^ peptides that were used for crystallization. These data support that binding of PELP1 provides stability to the SENP3 protease domain, which likely contributes to the observed increase in catalytic activity.

**Fig. 8.**
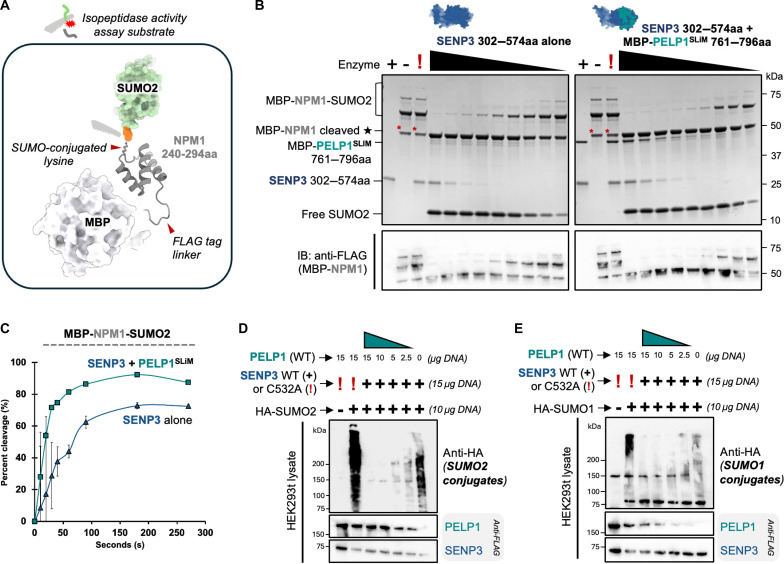
PELP1^SLiM^ stimulates SENP3 isopeptidase activity. (**A**) AlphaFold3 structural model of MBP-Flag-NPM1 (240 to 294aa) SUMO2 conjugate, with the cleavage site indicated with a red arrow. NPM1 SUMOylation site K263 depicted. (**B**) SDS-PAGE and total protein stain of in vitro SENP3 ± MBP-PELP1^SLiM^ isopeptidase activity assays against MBP-Flag-NPM1-SUMO2 conjugates. Decreasing concentrations (1000 to 3.9 nM) of SENP3 enzyme ± MBP-PELP1 761 to 796aa was incubated for 1 hour at 37°C with 15 μl of on-resin NPM1-SUMO2 conjugates. Cleaved free SUMO product is labeled. Red * on bands in control lanes denote MBP-Flag-NPM1 that copurifies without SUMO2 conjugation. (**C**) Quantification curves representing percent (%) cleavage of substrate over time correspond to the gel images in fig. S13B and the enzyme concentration used was 500 nM. SD was calculated from three independent experiments (*n* = 3). (**D**) SDS-PAGE and Western blot of HA-tagged SUMO2 conjugated proteins detected in HEK293FT cell lysate. Changes in amount of HA-SUMO2 conjugated proteins in vivo was assayed after transfecting cells with wild-type or C532A catalytic mutant SENP3 in combination with titrated transfection of wild-type PELP1. Decreasing amounts of transfected DNA expressing exogenous PELP1 with wild-type SENP3 resulted in an overall dose-dependent increase of HA-SUMO2–conjugated proteins similar to that observed with SENP3 C532A (catalytic-dead mutant). (**E**) Same as (D) except HA-tagged SUMO1 was used.

In addition to measuring SENP3 endopeptidase activity against proSUMO isoforms, we also used an in vitro assay to measure SENP3 isopeptidase activity against recombinant human NPM1 (nucleophosmin)-SUMO2 conjugates. NPM1 is a multifunctional nucleolar protein that plays an important role in mediating ribosome assembly and previous work established that SENP3 catalyzes the deSUMOlyation of NPM1-SUMO2 (*26*). To generate these NPM1 conjugates, we used an established in-cell *E. coli* SUMOylation system (*65*). We fused C-terminal NPM1 residues 240 to 294 to MBP for stability/purification and a FLAG tag linker for detection purposes (Fig. 8A). This C-terminal NPM1 region contains known in vivo SUMOylation sites in humans and has a known ordered structure (*66*, *67*). This NPM1 construct was coexpressed with SUMO conjugation machinery and His-tagged SUMO2 to produce NPM1-SUMO2 conjugates, which were subsequently purified by tandem affinity purification. We titrated increasing amounts of the SENP3 protease domain alone or SENP3 in complex with MBP-PELP1^SLiM^ with a fixed amount of NPM1-SUMO2 and resolved the products by SDS-PAGE (Fig. 8B). We further analyzed the products by Western blot using a FLAG antibody to confirm detection of NPM1-SUMO2 conjugates and deSUMOylated NPM1 (Fig. 8B). We quantified NPM1-SUMO2 substrate cleavage and observed a stimulation in SENP3 cleavage activity in the presence of the PELP1^SLiM^ (Fig. 8C). The observed level of SENP3 activation by PELP1 is higher for endopeptidase activity, suggesting that SENP3 is more robust at isopeptidase activity.

Beyond measuring SENP3 processing of proSUMO isoforms in vitro, we used a cell-based approach to investigate if SENP3 in the absence or presence of exogenous PELP1 influences global levels of SUMO1 and SUMO2 conjugates in cultured cells. We transfected cells with a plasmid harboring either HA-SUMO1 or HA-SUMO2, so that we could use an anti-HA antibody for detection of SUMO1/2 conjugate levels. First, we transfected cells with equal amounts of plasmids harboring wt-PELP1 and either wt-SENP3 or the SENP3-C532A catalytic mutant. Cells that overexpress the SENP3 C532A mutant show a substantial accumulation of SUMO2-conjugated proteins and to a lesser extent SUMO1-conjugated proteins in contrast to cells that overexpress wt-SENP3 (Fig. 8, D and E). In addition, we carried out a titration where we decreased the amount of wt-PELP1 but kept the amount of wt-SENP3 fixed. As we titrated down the amounts of PELP1, we observed a gradual increase in SUMO2 conjugates (Fig. 8, D and E). These data further support that PELP1 plays a role in stimulating SENP3 enzyme activity for both endo- and isopeptidase functions.

### Integrated model of the rixosome

**Fig. 9.**
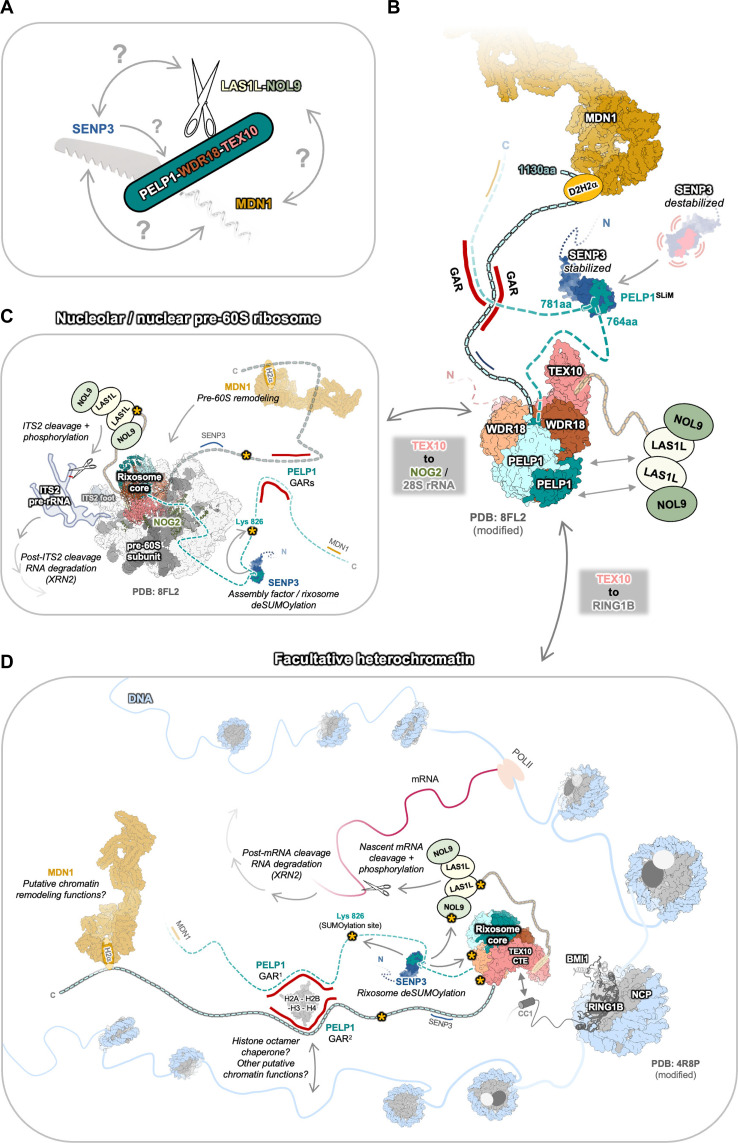
Integrated model of the human rixosome. (**A**) Swiss army knife model of the rixosome. The PELP1-WDR18-TEX10 scaffolding core is analogous to the housing unit of the knife from which the different diverse tools extend outward, including scissors (RNase PNK RNA cutting), saw blade (SENP3 protease), and corkscrew (MDN1 ATPase motor remodeling). (**B**) Cartoon structural model of the full human rixosome. The scaffolding core (PELP1-WDR18-TEX10) represents the stable core of the rixosome complex from which enzymatic components are connected. The RNA processing complex RNase PNK (LAS1L-NOL9) is physically and flexibly tethered to the stable scaffolding core. The deSUMOylation enzyme SENP3 and AAA-ATPase MDN1 are distinctly integrated with the rixosome through PELP1’s C-terminal IDRs. (**C**) Proposed architectural model of the human rixosome bound to the pre-60S subunit [PDB ID: 8FL2 (*3*)]. PELP1’s IDRs organize the enzymatic functions of SENP3 and MDN1. (**D**) Proposed architectural model of the human rixosome bound to polycomb and functioning in gene silencing at facultative heterochromatin. PELP1’s IDRs likely organize multiple enzymatic and nonenzymatic functions at heterochromatin, including SENP3 and MDN1 activity, and putative histone chaperoning by the GAR.

Our crystal structure of SENP3-PELP1^SLiM^ provides the structural basis for the integration of SENP3 within the rixosome complex, while our biochemical and structural modeling approaches provide additional molecular details on the dynamic PELP1 IDR. Our work here, in combination with recently published structures, allows us to generate an integrated model of the human rixosome (Fig. 9). The rixosome can be thought of as a molecular Swiss army knife, as it has a stable core to which flexible enzymatic modules are attached (Fig. 9A). The stable core of the rixosome is formed by two copies of the N-terminal Rix1 domain of PELP1 and two copies of WDR18, which form an integrated tetrameric assembly. One copy of TEX10 caps the core and provides further rixosome core stability and directionality by targeting the rixosome to either the pre-60S ribosome or PRC1 complexes at heterochromatin (Fig. 9, B to D). RNase PNK, the RNA processing enzymatic module, is tethered to the rixosome core in part by the flexible CTE of LAS1L, which binds to TEX10 (*3*) and a central helical domain that binds to WDR18 (*29*). The other two enzymatic modules of the rixosome, SENP3 and MDN1, are flexibly tethered to the core via the IDR of PELP1. The flexible enzyme tethers are likely a critical and signature feature of rixosome function to allow the enzymes to reach their distinct and often dynamic targets. For example, RNase PNK must be able to engage the ITS2 pre-rRNA cleavage site as well as a variety of nascent mRNAs at heterochromatin. MDN1 must be able to engage at least two distinct assembly factors at different stages of pre-60S assembly. SENP3 must engage a variety of SUMO deconjugation targets including almost all the members of the rixosome. One critical question that remains is the stoichiometry of factors that associate with the IDR of PELP1. There are two copies of PELP1 within the rixosome; thus, there are two binding sites for MDN1 and SENP3. Having the ability to engage two copies of MDN1 and SENP3 at the same time may provide enhanced flexibility to the rixosome and allow it to engage more than one substrate at a time. Future work will be needed to establish if both binding sites for these enzymatic modules can be occupied and functional simultaneously.

## DISCUSSION

Here, we used biochemical and structural approaches to build an integrated model of the human rixosome featuring PELP1 as the central scaffold (Fig. 9). By focusing on the PELP1 C-terminal IDR, we identified a series of interaction modules within this dynamic region that mediate association of the rixosome with SENP3, histones, and MDN1. The work presented here (along with many previous studies) supports several similarities and differences in the architecture and composition of the rixosome between yeast and higher eukaryotes (*3*, *4*, *16*, *29*). Previous cryo-EM structures revealed that the core of the rixosome is structurally well conserved. However, the C-terminal regions of human TEX10 and PELP1 are notably distinct from their yeast homologs. The ordered CTE of TEX10 extends the α-solenoid structure formed by the conserved N terminus. Our reconstitution experiments support that this entire alpha-solenoid structure is important for TEX10 and rixosome core stability. The extended α-solenoid forms a larger platform upon which interaction partners including NOG2, LAS1L, WDR18, and RING1B associate. Could the TEX10 CTE support rixosome recruitment to undiscovered targets in the nucleus? The TEX10 CTE is structurally large enough to facilitate multiple binding interactions. It will be interesting to investigate if the binding mechanism of external factors (i.e., RING1B/polycomb) to TEX10 is structurally similar or unique to that observed with NOG2 on the pre-60S ribosome. In contrast to TEX10, the entire C-terminal half of PELP1 is disordered, with the human IDR being several hundred amino acids longer than its yeast homolog Rix1. We establish that the MIH located at the very C terminus of the IDR facilitates an evolutionarily conserved interaction with the AAA-ATPase MDN1 through the specific D2H2α insert, where it may play a role in mediating MDN1 ATPase activity. Our work distinguishes the human PELP1 IDR from Rix1 through its unique SLiM and GAR regions, which mediate interactions with SENP3 and histones, respectively. In this way, the human rixosome Swiss army knife has acquired several additional tools compared to its yeast counterpart.

The first observation that PELP1 binds to histones was made over 20 years ago, but the importance of this association has remained unknown (*39*). Here, we establish that the PELP1 GAR region can function as a chaperone for the histone octamer in vitro. The acidic nature of the GAR helix likely mimics the phosphate backbone of DNA, providing stability to the octamer in its absence. The PELP1 GAR could function similarly to the chaperoning mechanism of APLF, the only other known chaperone for the histone octamer (*56*). Intriguingly, there is evidence that suggests that the GAR region of PELP1 can become even more acidic in vivo following glutamylation by TTLL4 (tubulin tyrosine ligase like 4), which catalyzes post-translational glutamic acid glutamylation (*52*). Previous work in pancreatic ductal adenocarcinoma cells identified TTLL4 as an interaction partner of PELP1, and knockdown of TTLL4 was shown to reduce glutamylation levels of the PELP1 GAR and subsequently decrease PELP1 association with histone H3 (*52*). Recent work demonstrated that glutamylation of histone chaperones Npm2 and Nap1 was shown to enhance histone stability and facilitate nucleosome assembly (*68*). Thus, it will be interesting to see how glutamylation influences PELP1’s putative histone chaperone function in future experiments.

The D/E tracts within the PELP1 IDR are prevalent in vertebrates, which corresponds with the emergence of the TEX10 CTE that specifically associates with PRC1. The coemergence of the putative histone chaperone GAR with the TEX10-PRC1 interaction (via RING1B) suggests that this chaperone function may be specifically linked to PRC1’s role in facultative heterochromatin maintenance. In *S. pombe*, the rixosome and Amo1 (nuclear rim protein) have been shown to collaborate with the histone chaperone FACT to promote the stability of heterochromatin by preventing histone turnover (*21*). In humans, in addition to promoting decay of nascent RNA, the rixosome also promotes the release of PolII (*1*). The GAR region of PELP1 may play roles similar to FACT by protecting against histone loss and/or promoting histone reassembly following release of PolII (*69*). Last, the GAR of PELP1 could also play a role in supporting histone masking by blocking access to modification enzymes to prevent additional histone modifications from occurring. Earlier studies demonstrated that overexpression of WT-PELP1 but not ΔGAR-PELP1 leads to a reduction in histone acetylation (*39*). Given that histone acetylation is a characteristic marker for active chromatin, histone masking by PELP1 may further contribute to PRC-mediated gene silencing.

Here, we identify a vertebrate-specific SLiM within the PELP1 IDR that mediates the protein-protein interaction between PELP1 and SENP3. SLiMs are commonly found in IDRs and considered one of the smallest structural and functional parts of modular eukaryotic proteins (*60*). SLiMs have been implicated in many biological processes including RNA processing (*70*). The PELP1 IDR contains another SLiM-like motif known as a SIM, just before the start of the GAR region (*10*). Most SIMs are composed of ~4 amino acids that adopt a β-strand conformation upon binding to SUMO (*71*). Our AlphaFold3 predictions model a high-confidence interaction between the PELP1^SIM^ and SUMO2 when engaged with SENP3’s protease active site (fig. S9, A and E to G). Within a 120aa segment, the PELP1 IDR contains a SENP3-specific SLiM (764 to 781aa), a known SUMO conjugation site (K826), and a SIM (880 to 883aa) (Fig. 3, B and C, and fig. S9). How these three areas coordinate with one another to promote rixosome function is still unclear but suggests that this region of the PELP1 IDR may facilitate several multivalent interactions. In our exogenous rixosome expression system, mutation of the known PELP1 SUMOylation site lysine 826 to arginine does not disrupt full rixosome assembly and purification (fig. S12). It is well established that almost all members of the rixosome can be modified by SUMO and have that SUMO removed in a SENP3-specific manner. The SUMO modification machinery has been proposed to regulate large protein-protein complexes like the rixosome through SUMO-SIM interfaces which might form “glue-like” interactions (*23*).

Our work provides a rationale for understanding why previous attempts at characterizing the activity of human SENP3 were unsuccessful. On its own, the SUMO protease domain of SENP3 is unstable and it relies on engagement of the PELP1^SLiM^ for allosteric activation. While the reliance on an activating factor distinguishes SENP3 from the other SENP family members, we identified a putative SLiM-like binding pocket in several other SENPs (fig. S8), hinting that this may be a common interface for facilitating protein-protein interactions within the SENP family. Beyond the members of the rixosome, SENP3 has a wide network of interaction partners and deconjugation targets (*23*, *72*). Given that the SLiM is a very small motif, it is tempting to speculate that there may be other SENP3 activating factors that await discovery as well as different modes of regulation of the SLiM-SENP3 interaction. For example, several studies have reported that SENP3 can function as a sensor of oxidative stress, but it remains unknown if this is a PELP1-dependent or independent activity of SENP3 (*73*–*75*). Last, the discovery that PELP1 is an allosteric activator of SENP3 opens the door for the rational design of SENP3-specific inhibitors/regulators. For example, a small SLiM-like peptide could have potential as an activator of SENP3 under hypoxic conditions. Last, our crystal structure could aid in structural based design of SENP3-specific protease inhibitors, which may have therapeutic potential for cancer therapy.

In summary, we establish that the human rixosome is a critical cellular Swiss army knife. This large multifunctional complex houses many important molecular tools including the endoribonuclease LAS1L, the PNK NOL9, the SUMO-specific protease SENP3, the AAA-ATPase MDN1, the targeting module TEX10, and at the center of it all the scaffold/chaperone PELP1. Together, these diverse tools coordinate their activities to help the cell drive two incredibly complex pathways including the production of ribosomes and the maintenance of facultative heterochromatin.

## MATERIALS AND METHODS

### Mammalian cell expression, affinity purification, and detection of rixosome complexes

HEK293FT cells (Thermo Fisher Scientific) were cultured in FreeStyle 293 Expression Media without supplementation at 37°C, 8% CO2, 80% humidity, 130 rpm shaking, and were used for exogenous human rixosome protein expression experiments/purifications. For cultures expressing rixosome subcomplexes (PELP1-TEX10-WDR18-SENP3), 40-ml suspension cultures of HEK293FT cells were transiently transfected with plasmid vector DNA using 293fectin reagent (Thermo Fisher Scientific) or Lipofectamine 3000 (Invitrogen), using the manufacturer’s protocol (1:2 DNA–to–transfection reagent ratio). All plasmids used in this paper are listed in table S1. Forty microliters of cultures were split in half upon harvest for a 20-ml cell pellet. One microgram of total DNA was used per 1 ml of cell culture except for titration experiments where microgram of DNA used is stated in respective figures. For cultures expressing the full rixosome complex, 100-ml suspension cultures were used for transient transfections in a similar manner with plasmids indicated in the figures, and cultures were split into 50 ml for harvesting and purifications. Amounts of DNA for individual plasmids cotransfected were equal. Cells were incubated with transfected DNA for at least 48 hours to allow for protein expression and were then harvested by centrifugation as cell pellets. Cell pellets were washed in 1× phosphate-buffered saline (PBS), flash-frozen in liquid nitrogen, and stored at −80°C until use.

Cell pellets containing transiently expressed rixosome protein complexes were lysed in 10 ml (50-ml cell pellet) or 5 ml (20-ml cell pellet) of lysis buffer [50 mM Hepes (pH 7.5), 200 mM NaCl, 5 mM Mg_2_Cl, 10% glycerol, 0.5% NP-40, 1 mM dithiothreitol (DTT), EDTA-free protease inhibitor (Roche), benzonase (±1.75 × 10^−4^ U/ml; Sigma-Aldrich)] for 30 min at 4°C with gentle agitation on a nutator. Whole-cell lysate was then clarified by centrifugation at 17,000*g* for 45 min at 4°C. The anti-FLAG M2 Affinity Gel (Sigma-Aldrich) was used for affinity isolation of FLAG-tagged PELP1 and SENP3 protein constructs used as bait in reconstituted rixosome complexes. An anti–green fluorescent protein (GFP) nanobody conjugated to Sepharose matrix was produced in house as described previously (*76*) and used for affinity isolation of GFP-tagged TEX10 protein constructs used as bait in reconstituted rixosome complexes. For larger-scale purifications, approximately 50 μl of equilibrated anti-FLAG affinity gel or anti-GFP affinity Sepharose was incubated with 14 ml of clarified and diluted input lysate per sample for 1 hour (4°C on a nutator). For smaller-scale purifications, approximately 30 μl of affinity matrix was used with 5-ml clarified lysate. Affinity matrices with bound rixosome protein complex were washed once with 10 ml of lysis buffer and thrice with 10 ml of low-salt wash buffer (50 mM Hepes pH 7.5, 200 mM NaCl, 5 mM Mg_2_Cl, and 10% glycerol). Protein-bound matrices were mixed with SDS loading dye and used for SDS-PAGE and Western blotting (described below). If native rixosome complexes were eluted from anti-FLAG affinity gel, 10 μl of washed and protein-bound anti-FLAG gel was taken first for SDS-PAGE and Western blotting. Remaining affinity gel was transferred to a SigmaPrep spin column (Sigma-Aldrich) and used for native elution of protein using 3× FLAG peptide. About 15 μl of 3× FLAG peptide (300 μg/ml; Pierce) in low-salt wash buffer was incubated with protein-bound anti-FLAG gel for 30 min at 4°C and on a nutator. Eluted protein complexes were collected by centrifugation and subject to SDS-PAGE and total protein staining overnight (SimplySafe Stain).

Denaturing SDS-PAGE tris-glycine gradient gels (4 to 15%) and 7.5% gels were used for Western blotting. Rixosome protein samples in SDS loading dye were boiled at a 70°C for 10 min. SDS tris-glycine buffer was used for electrophoresis at 200 V constant for 30 min (gradient gel) or 45 min (7.5% gel). Antibodies used for Western blotting in this study include the following: Polyclonal anti-FLAG produced in rabbit (Sigma-Aldrich, Cat# F7425, RRID:AB_439687), Monoclonal anti-FLAG M2-peroxidase HRP conjugate produced in mouse (Sigma-Aldrich, Cat# A8592, RRID:AB_439702), Monoclonal anti-HA produced in mouse (Thermo Fisher Scientific, Cat# 26183, RRID:AB_10978021), Monoclonal anti-GFP produced in mouse (Sigma-Aldrich, Cat# 11814460001, RRID:AB_390913), Monoclonal anti-MYC (clone 4A6) produced in mouse (Sigma-Aldrich, Cat# 05-724, RRID:AB_11211891), anti-PELP1 polyclonal antibody produced in rabbit (Bethyl, Cat# A300-180A, RRID:AB_242526), anti-WDR18 polyclonal antibody produced in rabbit (Sigma-Aldrich, Cat# HPA050200, RRID:AB_2681049), anti-TEX10 polyclonal antibody produced in rabbit (Thermo Fisher Scientific, Cat# 720257, RRID:AB_2633219), anti-SENP3 polyclonal antibody produced in rabbit (Bethyl, Cat# A303-139A, RRID:AB_10895725), anti-LAS1L polyclonal antibody produced in rabbit (Proteintech, Cat# 16010-1-AP, RRID:AB_2132810), anti-NOL9 polyclonal antibody produced in rabbit (Abcam, Cat# ab103207, RRID:AB_10712240), anti-MDN1 polyclonal antibody produced in rabbit (Sigma-Aldrich, Cat# HPA029666, RRID:AB_10600888), anti-Histone H3 polyclonal antibody produced in rabbit (Sigma-Aldrich, Cat# H0164, RRID:AB_532248), anti-RING1B monoclonal antibody produced in rabbit (Cell Signaling Technology, Cat# 5694, RRID:AB_10705604), anti-BMI1 polyclonal antibody produced in rabbit (Proteintech, Cat# 10832-1-AP, RRID:AB_2065392), anti-RYBP polyclonal antibody produced in rabbit (Proteintech, Cat# 11365-1-AP, RRID:AB_2285461), anti-mouse IgG (whole molecule)-Peroxidase antibody produced in rabbit (Sigma-Aldrich, Cat# A9044, RRID:AB_258431), anti-rabbit IgG, HRP conjugate antibody produced in goat (Sigma-Aldrich, Cat# 12-348, RRID:AB_390191), Normal Rabbit IgG (Millipore, Cat# 12-370, RRID:AB_145841). The manufacture’s recommended dilution ranges were used. After SDS-PAGE, separated proteins were transferred onto a polyvinylidene difluoride membrane (BioRad) using a Hoefer semi-wet transfer unit at 100 mA for 1 hour at room temperature. Membranes were blocked for 1.5 hour at room temperature in 5% milk in TBST (tris-buffered saline with 0.1% Tween-20) and then incubated with primary antibody overnight at 4°C. Primary antibody mixtures were in 5% milk and 1% bovine serum albumin (BSA) in TBST. The following morning, membranes were washed three times with TBST and incubated for 1 hour at room temperature with secondary antibody [anti-mouse horseradish peroxidase (HRP) or anti-rabbit HRP, 1:10,000 dilution] in 5% milk and 1% BSA in TBST. Membranes were then washed three more times in TBST, covered with ECL HRP substrate (Advansta), and exposed using a BioRad chemiluminescent imager. We performed at least three independent biological replicates of all co-IPs and Western blots included in the manuscript.

### In vitro TEX10-ncPRC1.4 binding assay

Recombinant human ncPRC1.4 protein complex (RING1B-BMI1-RYBP) was purified as previously described (*77*). In brief, full-length human SUMO-10×His-TEV-RING1B, BMI1, and RYBP were assembled into the pST44 polycistronic expression plasmid (*78*) in cassettes 2, 3, and 4, respectively. The complex was coexpressed in BL21(DE3) pLysS *E. coli* for 17 hours at 18°C. Cleared lysates were purified using metal-affinity chromatography using TALON Superflow resin (Takara). The SUMO-10×His tag on RING1B was cleaved with TEV protease and the RING1B-BMI1-RYBP complex was purified by cation exchange chromatography using Source S resin (GE Healthcare). Pooled fractions were dialyzed into 10 mM Hepes (pH 7.5), 200 mM NaCl, 10 mM 2-mercaptoethanol, and concentrated to 10 to 15 mg/ml and stored at −80°C following addition of 20% glycerol.

Rixosome subcomplexes with GFP-tagged TEX10 truncations were affinity purified from cells as described above with additional wash steps with ATP wash buffer [50 mM Hepes (pH 7.3), 150 mM NaCl, 20 mM Mg_2_Cl, 5% glycerol, and 5 mM ATP] three times each for 5-min incubations at 4°C. Three final low-salt buffer washes ensured removal of excess MgCl_2_ and ATP before incubation of immobilized TEX10 rixosome subcomplexes with ncPRC1.4 complex. In 0.2 ml volume of low-salt buffer, ncPRC1.4 protein (0.177 mg/ml) was incubated with immobilized TEX10 subcomplexes for 1 hour at 4°C with gentle mixing by pipette every 15 min. ncPRC1.4 binding to TEX10 subcomplexes was analyzed by SDS-PAGE and Western blotting as described above with endogenous ncPRC1.4 antibodies.

### Endogenous PELP1 protein co-IP

For co-IP of endogenous PELP1 protein, 20 ml of HEK293FT cell cultures with no exogenous rixosome complex were grown and harvested at a cell density of 1.0 × 10^6^ cells/ml. Cell protein lysates were prepared as explained above. Two microliters of clarified lysate was incubated for 1 hour at 4°C on a nutator with 1 μg of anti-PELP1 antibody (Bethyl labs) or 1 μg of normal rabbit immunoglobulin G (Sigma-Aldrich) for negative control. Forty microliters of protein G Sepharose resin slurry (Invitrogen) was added for another 1-hour incubation in the same conditions. Protein-bound resin with endogenous protein interaction targets were washed and analyzed as described above for exogenous rixosome complexes.

### Purification of recombinant PELP1 C-terminal IDR peptides from *E. coli*

6×-HIS-MBP tagged PELP1 C-terminal IDR peptides (761 to 796aa, 800 to 1130aa, 887 to 966aa, and 967 to 1130aa) were used in this study for in vitro binding experiments. *E. coli* expressing these PELP1 IDR fragments were grown to an optical density at 600 nm (OD_600_) of 1.20 and allowed to express protein overnight at 18°C. For 6×-HIS-MBP-PELP1 761 to 796aa (SENP3 binding motif peptide), harvested cell pellets were resuspended in PO_4_ lysis buffer [2× PBS without Ca/Mg (pH 8.0), 750 mM final NaCl, 10% glycerol, and 10 mM imidazole (pH 8.0)] with final total phosphate concentration of about 19 mM. Resuspended cells had two drops of Triton X-100 detergent and one EDTA-free protease inhibitor tablet (Roche) added per 50 ml volume (about 2-liter harvested cells). This resuspension was then sonicated 2-s on, 2-s off with 70% amplitude at 4°C for 3 min per 2-liter cell material (9 min for 6-liter cell harvest). Cell lysate was immediately clarified by centrifugation at 27,000*g* at 4°C. Clarified cell lysate was slowly gravity flowed over His60 Ni Superflow resin (Clontech Labs Inc) at 4°C. His60 resin with bound HIS-tagged PELP1 protein was washed by gravity flow with lysis buffer (200× resin bed volume) and then eluted with 5× resin bed volume lysis buffer containing 300 mM imidazole (pH 8.0). Eluted protein was concentrated by centrifugation (30 kDa cutoff, Millipore Sigma) to about 1 ml volume and then immediately injected onto a S200 gel filtration column (Superdex 10/300 Increase, GE Healthcare) equilibrated with S200 buffer [1× PBS without Ca/Mg (pH 8.0), 200 mM final NaCl, 10% glycerol, and 1 mM DTT] with final total phosphate concentration of about 9.5 mM. Protein elutions of HIS-MBP-PELP1 761 to 796aa from gel filtration were collected, combined, and concentrated to at least 100 μM before aliquoting and flash-freezing in liquid nitrogen for storage at −80°C. For purification of 6×-HIS-MBP-PELP1 800 to 1130aa and 887 to 966aa (GAR-containing peptides), harvested cells were resuspended in Hepes lysis buffer [50 mM Hepes (pH 7.5), 750 mM NaCl, 10% glycerol, and 10 mM imidazole (pH 8.0)]. Cell lysis by sonication, lysate clarification, and His60 resin binding was performed as described above. His60 resin bound protein was washed with lysis buffer (100× resin volume), followed by 100× resin volume high-salt buffer [10 mM Hepes (pH 7.5), 1.2 M NaCl, and 10 mM imidazole (pH 8.0)]. Protein was eluted from His60 resin using 5× resin bed volume high-salt wash buffer with 300 mM imidazole (pH 8.0). Eluted protein was concentrated by centrifugation (30 kDa cutoff, Millipore Sigma) to about 0.5 ml volume and then immediately injected onto a S200 gel filtration column as described above but with high- or low-salt equilibration buffer [10 mM Hepes (pH 7.5) and 1.2 or 0.2 M NaCl] depending on the experiment downstream. Protein fractions of recombinant MBP-fused PELP1 peptides from gel filtration were collected, combined, and concentrated to at least 50 μM before aliquoting and flash-freezing in liquid nitrogen for storage at −80°C.

### Expression and purification of recombinant human SENP3 and SENP5 proteins from *E. coli* for protease activity assays

N-terminal 6× His-tagged human SENP3 protease domain (302 to 574aa, *E. coli* codon-optimized gene synthesized by Genscript in pET11a vector) and SENP5 protease domain [567 to 755aa, plasmid no. 16358 (*61*) from Addgene in pET28a vector] were used for in vitro binding and deSUMOylation assays. *E. coli* expression SENP3 302 to 574aa were grown to OD_600_ of 1.00 at 37°C, moved to incubators shaking at 16°C for 30 min, then induced with 0.5 mM isopropyl-β-d-thiogalactopyranoside (IPTG) and allowed to express overnight at 16°C. For SENP5 567 to 755aa, growth and induction were the same as SENP3, but protein expression was performed at 30°C for 5 hours. His-tag purification of SENP3 and SENP5 protease domains was performed identically. Cell pellets were resuspended in PO_4_ lysis buffer [2× PBS without Ca/Mg (pH 8.0), 750 mM final NaCl, 10% glycerol, and 10 mM imidazole (pH 8.0)] with final total phosphate concentration of about 19 mM. Resuspended cells had two drops of Triton X-100 detergent and 1 EDTA-free protease inhibitor tablet (Roche) added per 50 ml volume. Sonication and lysate clarification was done as described previously above. Clarified cell lysate was batch bound to His60 resin in 50-ml tubes (max volume) for 1 hour at 4°C on nutator. Protein bound His60 resin was moved to gravity flow column and washed with lysis buffer (200× bed volume) followed by protein elution with lysis buffer with 200 mM imidazole (pH 8.0; 5× bed volume elution). His60 elution was concentrated by centrifugation (15 kDa cutoff Amicon concentrator) to about 1 ml volume and immediately injected onto an S200 gel filtration column equilibrated in S200 buffer [1× PBS without Ca/Mg (pH 8.0), 200 mM final NaCl, 10% glycerol, and 1 mM DTT]. Gel filtration fractions containing SENP3 or SENP5 protease domains were collected, combined, and concentrated by centrifugation as before. SENP3 protein was concentrated to lower amounts (about 40 μM) due to stability/precipitation issues at higher concentrations. SENP5 protein was concentrated to about 80 μM and all protein was aliquoted, flash-frozen, and stored at −80°C before use in biochemical assays.

### In vitro SENP3-PELP1 761 to 794aa peptide binding assays

All protein binding assays were performed with purified protein components and assessed for binding by gel filtration (size exclusion) chromatography using S200 10/300 analytical columns operated via an AKTA FPLC system (Cytiva). Individual protein components were mixed at equimolar amounts (ranged from 20 to 80 μM) to a final volume of 120 μl. Mixed samples were incubated at 4°C for at least 30 min and then 100 μl was injected onto the gel filtration column. Abs_280_ allowed for monitoring of protein shifts in column volume retention, corresponding to protein complex formation. Protein fractions were collected from gel filtration runs and analyzed by SDS-PAGE and total protein staining as describe above. This method was used for larger scale reconstitution and purification of the SENP3-PELP1 761 to 794aa complex used for proSUMO endopeptidase assays describe below. This complex was fractionated, concentrated, flash-frozen, and stored at −80°C.

### Expression and purification of recombinant human proSUMO1, 2, and 3 isoforms from *E. coli*

For expression and purification of proSUMO isoforms used for endopeptidase assays, established protocols were used (*61*). In brief, *E. coli* cells expressing full-length proSUMO isoforms with C-terminal 6×-HIS tag (Addgene plasmid nos. 25101, 25102, and 25103, pET28a vector backbone) were grown at 37°C to an OD_600_ of about 1.2, induced with 0.4 mM IPTG at this same temperature, and allowed to express for 3 hours before harvest. HIS tag affinity purification was performed with same steps and buffers as the earlier subsection for SENP3/5 purification, except gel filtration chromatography was performed with an S75 10/300 column (Cytiva). Purified proSUMO isoforms were concentrated, aliquoted, flash-frozen, and stored at −80°C before use in assays.

### Endopeptidase protease cleavage assays of proSUMO substrates

Purified protein components used for assays were stored at −80°C and thawed on ice before mixed in the reaction tubes. SENP3-PELP1 peptide complex was reconstituted as a stoichiometric complex from individual components as described below in the in vitro binding assays subsection and used in protease assays. Each protease cleavage reaction was performed in a final volume to 20 μl. Final reaction buffer was 1× PBS without Ca/Mg (pH 8.0), 200 mM final NaCl, 10% glycerol, and 5 mM DTT. Final reaction protein concentrations were 5 μM for proSUMO substrate and a titrated range of SENP3/5 proteases 2.5 μM to 0.12 nM. Ten microliters of protease substrate (proSUMOs) at 10 μM were first aliquoted in all tubes on the bench at room temperature in reaction buffer containing 10 mM DTT. Ten microliters of prior titrated concentration stocks of SENP3/5 proteases on ice in buffer without DTT were added to the tubes containing proSUMO substrate and gently mixed by pipetting. For enzyme titration assays, mixed reactions were incubated in a standard chamber at 37°C for 1 hour. For time course assays ranging from 0 to 450 s, a microtube thermomixer was used for 37°C incubations for specific times. All reactions were stopped by addition of 2× SDS dye containing 2-ME. Samples were analyzed by SDS-PAGE and total protein staining using 16% Tricine-SDS gels or 4 to 20% bis-tris gels (Thermo Fisher Scientific). Gels were run at 100 V constant for 2 hours before total protein staining overnight. Gels were allowed destain in water completely before acquiring images of gels with a BioRad ChemiDoc-IT imager.

### Expression and purification of recombinant NPM1-SUMO2 conjugates from *E. coli*

Established protocols were used to generate NPM1-SUMO2 conjugates. In brief, *E. coli* cells coexpressing MBP-FLAG-NPM1 (240 to 294), full-length SUMO2 with a C-terminal 6×-HIS tag and the human SUMO conjugation machinery (pSUMO2 vector, Addgene plasmid no. 52259) were grown in LB media with ampicillin (50 μg/ml) and streptomycin (25 μg/ml) at 30°C to an OD_600_ of about 0.7. Cultures were induced with 1.0 mM IPTG at this same temperature and allowed to express overnight at 16°C before harvest. Cells were lysed by sonication in PO_4_ lysis buffer, clarified and bound to His60 resin. The resin was washed with lysis buffer (100× bed volume) and protein was eluted with lysis buffer containing 300 mM imidazole (pH 8.0; 10× bed volume). The His60 elution was then diluted to 15 ml with a low-salt wash buffer [1× PBS without Ca/Mg (pH 8.0), 200 mM NaCl, and 10% glycerol] and incubated with amylose resin for 2 hours at 4°C on a nutator. The resin was washed with low-salt buffer (100× bed volume) and kept at 4°C on resin before using as the substrate for isopeptidase assays.

### Isopeptidase protease cleavage assays of NPM1-SUMO2 substrates

Isopeptidase assays were largely performed as described above for the endopeptidase assays except for the notable difference that MBP-NPM1-SUMO2 substrates remained bound to amylose resin for protease assays. Fifteen microliters of substrate-bound amylose resin slurry was used for each reaction with a final reaction volume of 30 μl. SENP3 titration range was 1000 to 3.9 nM. Time course assays were performed with an enzyme concentration of 500 nM. Samples were analyzed by SDS-PAGE and total protein staining using tris-glycine 4 to 15% gels (BioRad).

### Detection of HA-SUMO2–conjugated proteins in suspension cells

HEK293T cells were grown in suspension and transfected with indicated plasmids as described above. Cells were harvested, washed with cold 1× PBS containing 100 mM iodoacetamide (IAA), and processed immediately. For cell lysate preparation in which HA-SUMO2–conjugated proteins were detected, the same mammalian cell lysis buffer as described above was used with addition of 75 mM IAA. Harvested cells were resuspended in this lysis buffer and incubated on ice for 20 min with occasional gently pipetting. Crude cell lysate was gently centrifuged at 500*g* for 2 min at 4°C to collect large insoluble cell debris and the lightly clarified supernatant was collected, immediately mixed with 2× or 4× SDS dye containing 2-ME, and boiled at 90°C for 10 min. The pH of this lightly clarified cell lysate was adjusted with 0.5 M tris (pH 7.5) until proper color indication of the SDS dye was maintained by eye. Prepared lysate was then analyzed by SDS-PAGE and Western blot to detect HA-SUMO2–conjugated proteins.

### Expression, purification, and reconstitution of recombinant human histone octamers

H2A/H2B dimers and H3/H4 tetramers were prepared using canonical core histones (H2A1, H2B1C, H3.2, and H4) as previously described (*54*, *79*, *80*). In brief, individual histones were expressed in BL21 (DE3) pLysS *E. coli* for 3 hours at 37°C using previously reported histone expression plasmids. Histones were extracted from washed inclusion bodies as previously described. H2A/H2B dimers and H3/H4 tetramers were reconstituted by combining extracted histones in equimolar concentrations in unfolding buffer [20 mM tris-HCl (pH 8.0), 7 M guanidine-HCl, and 10 mM DTT] and dialyzing into refolding buffer [10 mM Hepes (pH 7.5), 100 mM NaCl, and 10 mM 2-mercaptoethanol (βME)] overnight at 4°C. Reconstituted histone complexes were purified by cation exchange chromatography using a Source S resin (GE Healthcare). Pooled fractions were concentrated to 10 to 15 mg/ml and stored at −80°C following addition of 20% glycerol.

Using the H2A-H2B histone dimer and H3-H4 histone tetramer complexes, histone octamers were reconstituted using established methodology (*79*). In brief, histone dimer and tetramer complexes were mixed in fresh unfolding buffer [6 M guanidinium chloride, 20 mM tris-HCl (pH 7.5), and 5 mM DTT] and then dialyzed against refolding buffer overnight [2 M NaCl, 10 mM tris-HCl (pH 7.5), 1 mM Na-EDTA, and 5 mM 2-βME]. The next day, refolded histone octamer complexes were purified by S200 gel filtration in high-salt refolding buffer to separate them from free histone dimers and tetramers that remained. Pure octamers were collected, concentrated, and used immediately for histone chaperoning assays with PELP1 C-terminal GAR peptides (described below).

### PELP1-histone octamer chaperoning assay

Visualization of this experimental method is illustrated in Fig. 5B. Purified MBP-PELP1 800 to 1130aa or 887 to 966aa and histone octamer complex stored in high-salt buffers (>1.2 M NaCl) were mixed at 1:1 or 2:1 molar ratio in a final volume of 120 μl. This protein mixture was subject to salt dialysis in 3 kDa cutoff microdialysis buttons (Thermo Fisher Scientific) through four buffer incubation steps in decreasing NaCl concentrations (1.2 M, 0.8 M, 0.6 M, and 0.3 M NaCl). Additional buffer component was 10 mM Hepes (pH 7.5) only. All dialysis steps were conducted at 4°C. The first three dialysis conditions were performed for 1 hour each in 1-liter total buffer with gentle stirring. The last dialysis condition (300 mM NaCl) was allowed to incubate overnight in 2-liter total buffer volume with gentle stirring. The next morning, samples were removed from the microdialysis buttons and 100 μl injected onto an S200 10/300 analytical gel filtration column equilibrated with the final low-salt dialysis buffer to assess protein binding/chaperoning. Protein fractions were collected from gel filtration runs and analyzed by SDS-PAGE and total protein staining as described above.

### Cloning, expression, and purification of fixed-arm MBP-SENP3 catalytic domain fusion for crystallography

Constructs of the human SENP3 catalytic domain were designed on the basis of sequence alignments with other SENP proteases (*61*) and secondary structure prediction in PSIPRED (*81*). SENP3 and SENP5 are both predicted to contain a three-helix bundle (SENP3 residues Arg^310^-Arg^369^) N-terminal to the core catalytic domain, which has been included in the constructs used in this study. A catalytic-dead mutant of SENP3 (C532S) was generated using QuikChange mutagenesis. The SENP3 sequence encoding Glu^311^-Val^574^ of the catalytic-dead mutant was cloned between the NotI and BamHI restriction sites of the pMALX(wt) expression vector, generating a fixed-arm fusion of SENP3 C-terminal to MBP, to be expressed and purified for crystallization. pMALX(wt)-SENP3 (Glu^311^-Val^574^/C532S) was transformed into Rosetta2 (DE3) cells for expression. The cells were grown in Terrific broth supplemented with ampicillin (100 μg/ml) and chloramphenicol (35 μg/ml), with shaking at 37°C to a OD_600nm_ of 0.8 to 1.0, at which point the temperature was decreased to 16°C for 35 min, and protein expression was induced by addition of IPTG to a final concentration of 0.4 mM. Protein expression continued overnight at 16°C. The cells were pelleted by centrifugation and lysed by sonication in 2× PBS (without calcium or magnesium), 750 mM NaCl (final concentration), and 10% (v/v) glycerol. The lysate was clarified by centrifugation and bound in-batch to amylose resin (New England Biolabs). Bound MBP-SENP3 was eluted from the amylose resin in-batch using 2× PBS, 750 mM NaCl (final concentration), 10% (v/v) glycerol, 40 mM maltose, and further purified by SEC on a Superdex200 26/600 column in elution buffer. SEC fractions containing the purified protein were pooled and dialyzed overnight at 4°C to 20 mM Hepes (pH 7.3), 200 mM NaCl, 5% (v/v) glycerol, and 5 mM maltose. The purified proteins were concentrated, flash-frozen in liquid nitrogen, and stored at −80°C.

### Cocrystallization of MBP-SENP3 with PELP1 peptide

The SENP3-interacting peptides from protein binding partner PELP1 (residues Ala^764^-Glu^781^, sPELP1 or residues Ala^764^-Pro^792^, lPELP1) were synthesized by GenScript. The lyophilized peptide (henceforth referred to as sPELP1) was resuspended to 10 mM in 3% (v/v) ammonia water, as specified by the manufacturer. sPELP1 was further diluted in 0.897 M Hepes (pH 7.5), 100 mM NaCl, and 5% (v/v) glycerol to 2.5 mM. MBP-SENP3 (6.68 mg/ml) was mixed with 1.5× molar excess of the sPELP1 peptide (final concentration of 5.90 mg/ml of MBP-SENP3) and allowed to equilibrate on ice for 2 hours. Crystals of the MBP-SENP3/sPELP1 complex were grown using the sitting drop vapor diffusion technique by mixing 400 nl of the complex with 200 nl of mother liquor [0.085 to 0.1 M Hepes (pH 7.5), 17 to 20% (w/v) PEG8000] at 4°C. Crystals were harvested by opening the drop and adding 2× 1 μl volumes of cryoprotectant [0.1 M Hepes (pH 7.5), 200 mM NaCl, 5 mM maltose, 20% (w/v) PEG8000, and 17% (v/v) glycerol, supplemented with 125 μM sPELP1 peptide], allowing for a short equilibration between additions. Transferred crystals from this drop were directly added to cryoprotectant solution and flash-frozen liquid nitrogen.

### Data collection and refinement

Crystals of the MBP-SENP3/sPELP1 complex were sent to the AMX beamline [17-ID-1 (*82*)] at the National Synchrotron Light Source II (NSLSII) at Brookhaven National Laboratory for data collection. The crystals were placed into a stream of nitrogen gas cooled to −180°C during collection of diffraction data. The data were integrated and scaled using the autoPROC toolbox (*83*) and an Rfree test set was randomly generated. The phase problem was solved by molecular replacement in Phaser (*84*), using a previously published MBP structure [PDB ID code 6XRX (*58*)], and the SENP3 three-helix bundle and catalytic core subdomains predicted by AlphaFold3 (*85*) as separate search models. The final structure was refined by iterative rounds of manual model building in COOT (*86*, *87*) and refinement in Phenix (*88*). Translation/libration/screw vibrational motion refinement (*89*) was used. All 18 residues of the sPELP1 peptide used for cocrystallization are ordered and included in the final model. Data collection and refinement statistics are listed in Table 1. The Ramachandran statistics were generated by MolProbity (*90*). Residues in MBP are labeled 1 to 370, while those from SENP3 are labeled as 1311 to 1574 in the models. All structural images, superpositions, and electron density maps used for figures were created using UCSF ChimeraX version 1.8 (*91*) with ISOLDE plugin.

### Differential scanning fluorimetry

Wild-type SENP3 apoprotein (4.59 mg/ml) was diluted to 0.6 mg/ml concentration in protein storage buffer [20 mM Hepes (pH 7.3), 200 mM NaCl, and 10% (v/v) glycerol]. Complex mixtures of SENP3 and 1.5× molar excess of either sPELP1 or lPELP1 were assembled as for cocrystallization above, then diluted to 0.6 mg/ml final SENP3 concentration using protein storage buffer. A 40× Sypro Orange was added to a final concentration of 5× in a 20 μl reaction volume. Melt curves were performed on a QuantStudio 7 Flex (ABI). The samples were placed at 25°C for 2 min, then the temperature was increased linearly to 95°C, with a ramp rate of 0.05°C/s, followed by a 2-min incubation at 95°C. *T*_m_ values were derived via the Boltzmann equation (not reported) and the first derivative (reported in Fig. 7F), using the Protein Thermal Shift software version 1.3 (ABI), and are shown as the melting temperature ± SD. Reactions were performed in quadruplicate.

### Multiple sequence alignments and PELP1 IDR polypeptide informatics

Protein primary sequences were acquired from UniProt and National Center for Biotechnology Information protein databases. Sequence alignments were generated using PROMALS3D web server (*92*, *93*), and known experimental structures were used to guide alignments if deposited in the PDB. Generated alignments were visualized and edited in Jalview software (*94*). Public domain computational resources were used to dissect the PELP1 C-terminal IDR (643 to 1130aa). AlphaFold3 was used to visualize any predicted secondary structure present (*41*). IUPred3 was used for overall protein disorder scores throughout the IDR (*95*). Prediction scores of polyproline secondary structure helices (PPII) within the PELP1 IDR was obtained using PPIIPRED (*96*).

### AlphaFold protein complex structural predictions

AlphaFold structural predictions of individual human rixosome protein members were acquired from the updated AlphaFold database (*85*, *97*). For all protein complex predictions, the newest AlphaFold3 online server was used (*41*). All predicted aligned error (PAE) plots for each prediction shown were visualized using the online PAE viewer (*98*). Model confidence scores (pLDDT) for protein regions of interest are colored accordingly. Conventional model confidence levels of very high (pLDDT > 90), confident (90 > pLDDT > 70), low (70 > pLDDT > 50), and very low (pLDDT < 50) were used. Sequence information for all predicted models from this study are indicated in the figures, and all predicted model coordinates will be deposited at an online data repository before publication.

### Statistical analysis

#### Quantification of endopeptidase and isopeptidase protease cleavage assays

Raw gel images were used for densiometric analysis of protein bands using Image Lab software (BioRad). Gel images used for SUMO protease time course assays are included in fig. S13. For endopeptidase assays, the percent substrate cleavage was calculated by analyzing the ratio of cleaved SUMO product versus uncleaved proSUMO substrate in each sample. For isopeptidase assays, the percent substrate cleavage was calculated by analyzing the disappearance of MBP-NPM1-SUMO2 substrate over time against uncleaved substrate not in the presence of enzyme. SDs were calculated from three independent experiments at the same protein concentrations. Microsoft Excel was used for plot generation and calculations. Statistical significance was not calculated.

## Supplementary Materials

### This PDF file includes:

Figs. S1 to S13 Table S1 References
