# Supplementary material for: PELP1 coordinates the modular assembly and enzymatic activity of the rixosome complex
Source: Sci Adv. 2025 Jul 25;11(30):eadw4603. doi: 10.1126/sciadv.adw4603 (PMC12292926; doi:10.1126/sciadv.adw4603)
Supplement: Supplementary file 1 — Figs. S1 to S13 Table S1 References [file sciadv.adw4603_sm.pdf]

Science Advances
AAAS

Supplementary Materials for

PELP1 coordinates the modular assembly and enzymatic activity of the rixosome complex

Jacob Gordon *et al.*

Corresponding author: Alan J. Warren, ajw1000@cam.ac.uk; Robin E. Stanley, robin.stanley@nih.gov

Sci. Adv. **11**, eadw4603 (2025)
DOI: 10.1126/sciadv.adw4603

This PDF file includes:

Figs. S1 to S13
Table S1
References

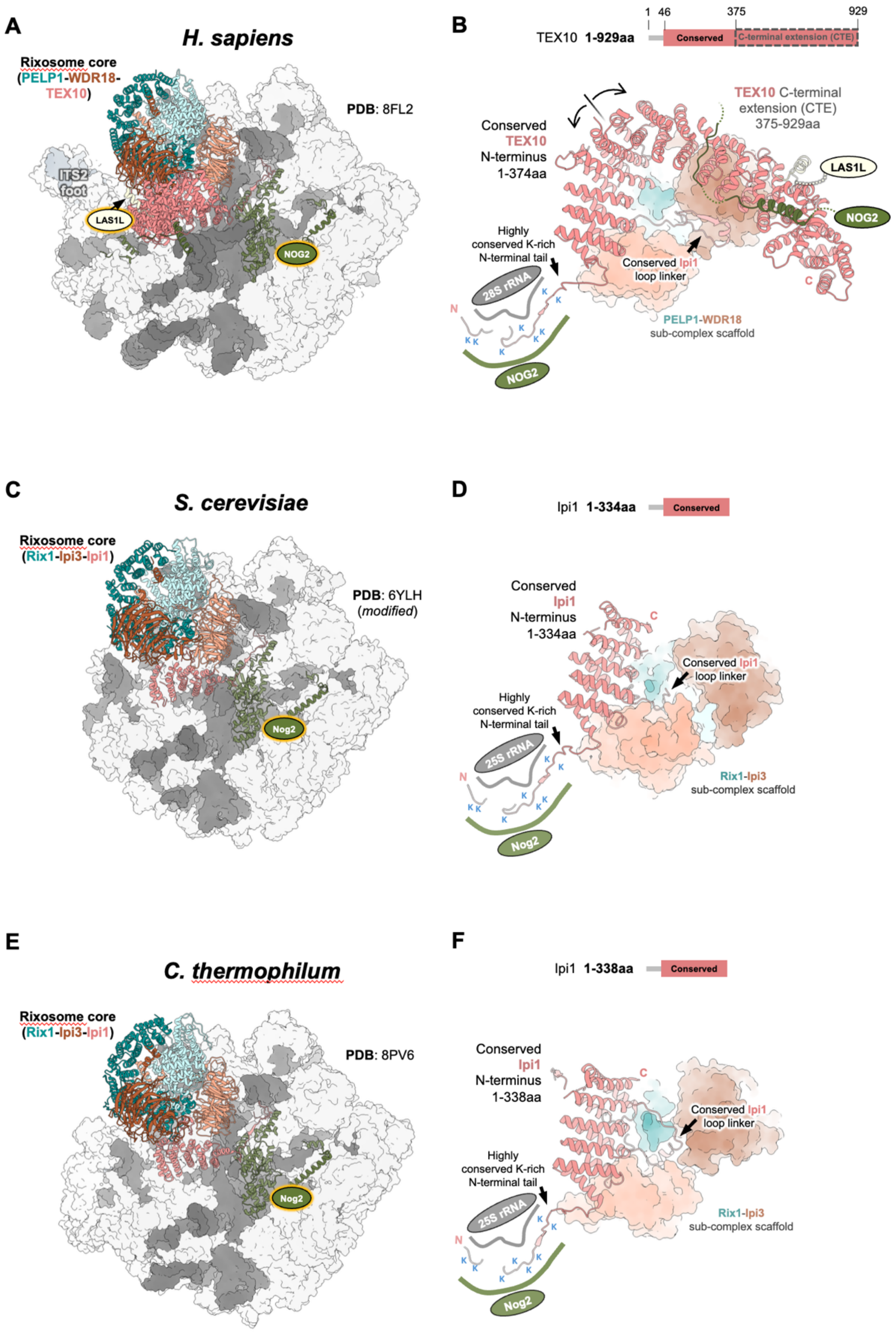

Fig. S1. Structures of pre-60S particles containing the rixosome complex, related to Fig. 1. (A) Cryo-EM structure of human pre-60S bound to the rixosome core (PDBID: 8FL2(3)). The two copies of PELP1 are colored in two shades of teal, the two copies of WDR18 are colored in two

shades of orange, while TEX10 is colored salmon. The assembly factor NOG2 is colored in green, and small helix from LAS1L is colored light yellow. Ribosomal proteins and other assembly factors are shown as a light gray surface while the rRNA is colored in dark grey.(B) Cartoon schematic of human TEX10 and a zoomed in view of the TEX10 interactions depicted in panel panel A. (C) Cryo-EM structure of *Saccharomyces cerevisiae* pre-60S bound to the rixosome core (PDBID 6YLH(*30*)). Please note for clarity Rea1 was omitted from this view. Colors are the same as indicated for panel A. (D) Cartoon schematic of *S. cerevisiae* Ipi1 (Tex10) and a zoomed in view of the Ipi1 interactions shown in panel C. (E) Cryo-EM structure of *Chaetomium thermophilum* pre-60S bound to the rixosome core (PDBID 8PV6(16)). Colors are the same as indicated for panel A. (F) Cartoon schematic of *C. thermophilum* Ipi1 (Tex10) and a zoomed in view of the Ipi1 interactions shown in panel C.

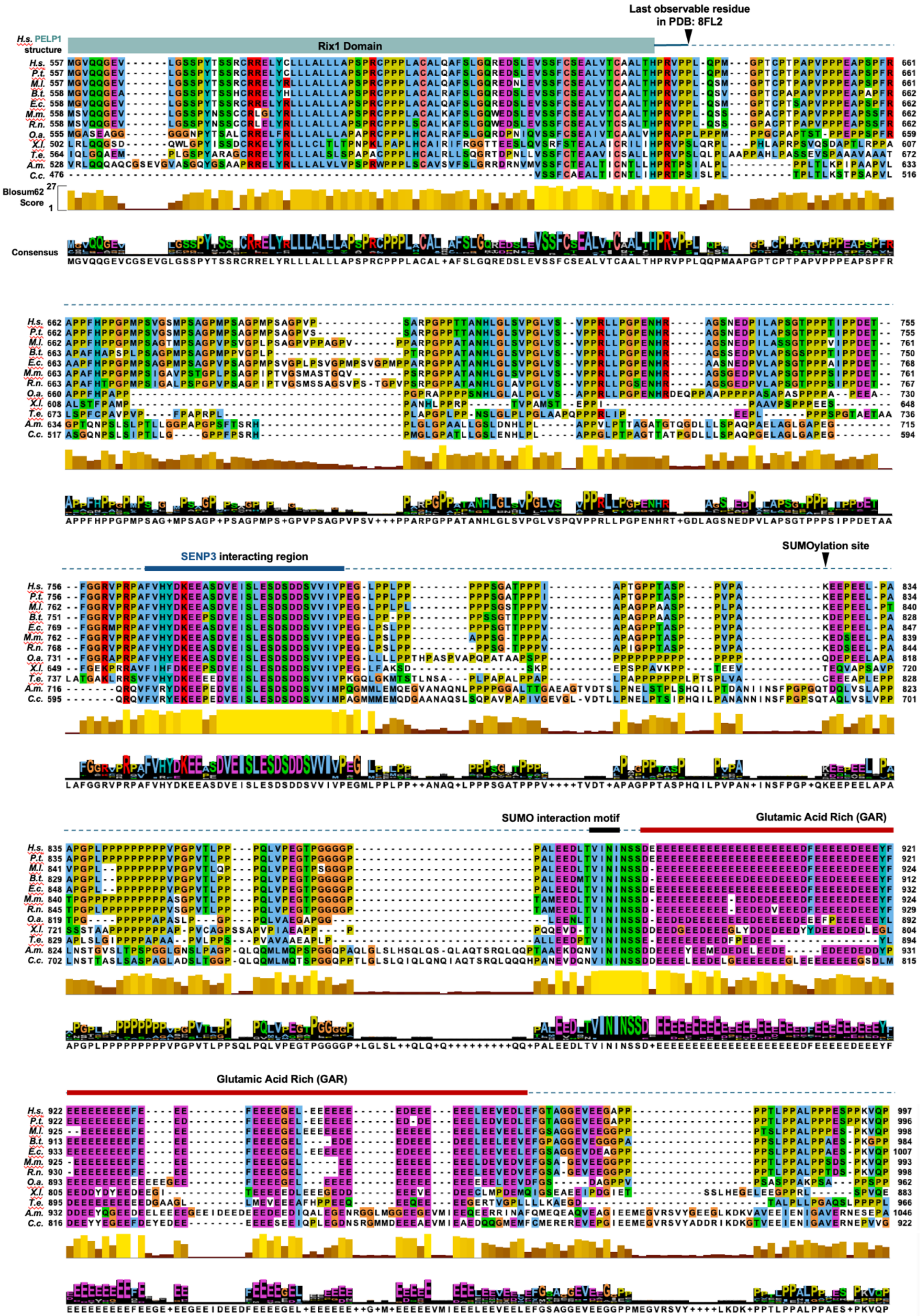
Last observable residue in PDB: 8FL2
H.s. PELP1 structure
Rix1 Domain
H.s.
P.t.
M.l.
B.t.
E.c.
M.m.
R.n.
O.a.
X.l.
T.e.
A.m.
C.c.
Blosum62 Score
Consensus
SENP3 interacting region
SUMOylation site
SUMO interaction motif
Glutamic Acid Rich (GAR)
Glutamic Acid Rich (GAR)

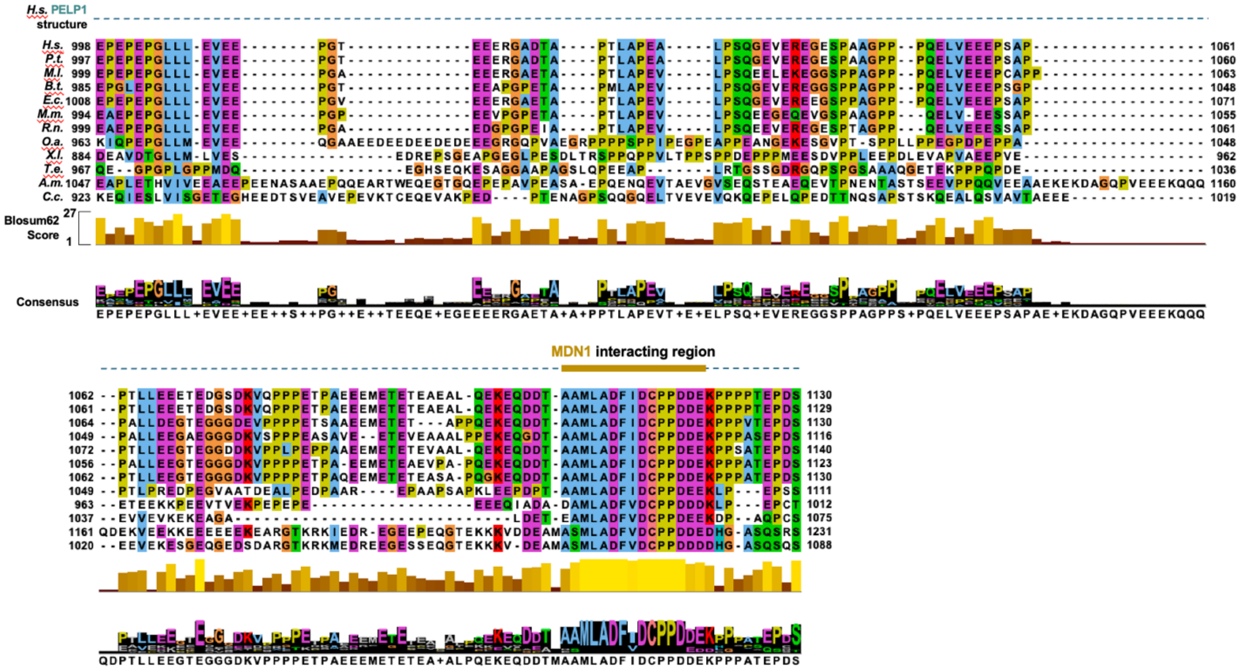

Fig. S2. Comprehensive sequence alignment of the IDR of mammalian PELP1 homologues. The multiple sequence alignment of PELP1 IDR was visualized using Jalview (*94*). Species abbreviations: *Homo sapiens* (H.s.), *Pan troglodytes* (P.t.), *Myotis lucifugus* (M.l.), *Bos taurus* (B.t.), *Equus caballus* (E.c.), *Mus musculus* (M.m.), *Rattus norvegicus* (R.n.), *Ornithorhynchus anatinus* (O.a.), *Xenopus laevis* (X.l.), *Thamnophis elegans* (T.e.), *Astyanax mexicanus* (A.m.), *Cyprinus carpio* (C.c.). Residue-specific Blosum62 scores and consensus annotations are shown below the alignment.

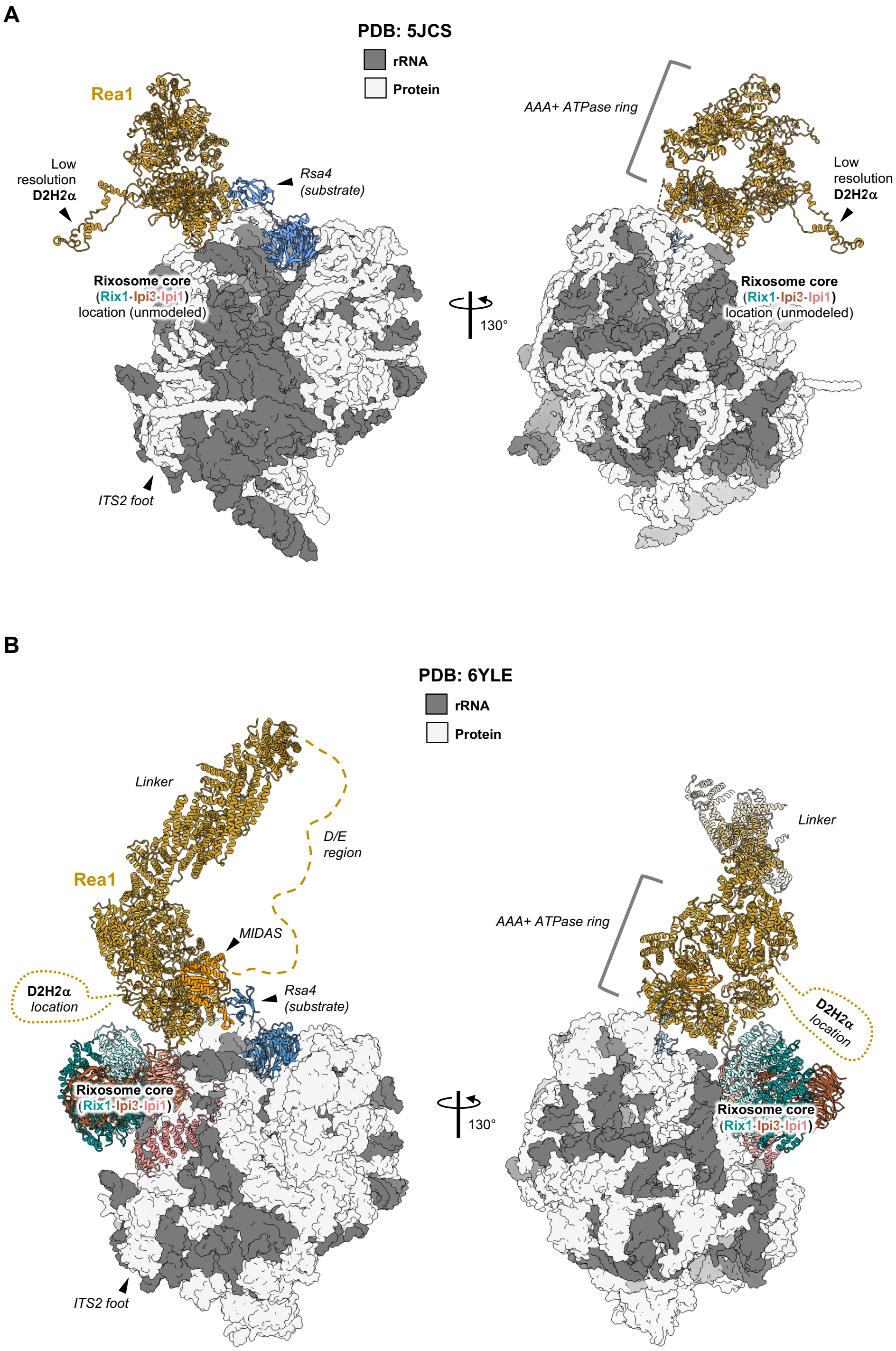

Fig. S3. Structures of *S. cerevisiae* pre-60S particles bound to Rea1 and the rixosome core, related to Fig. 4. (A) Cryo-EM reconstruction (resolved between 7 and 16 Å resolution) of Rea1(yeast homologue of human MDN1, gold) and the rixosome core (unmodeled) bound to the pre-60S (PDBID 5JCS (*42*)). The assembly factor Rsa4, which is a substrate of Rea1, is shown in blue. All other ribosomal proteins and assembly factors are colored in light grey while the rRNA is colored in dark grey. Weak density was visible for the D2H2α insert next to

approximate location of the rixosome core. (B) Multibody refined cryo-EM reconstruction of Rea1 (6.6 Å resolution) and the rixosome core (3.3 Å resolution) bound to the pre-60S (PDBID 6YLE (*30*)). The resolution was sufficient to build a model of the rixosome core components, but no high-resolution density was visible for the D2H2α insert.

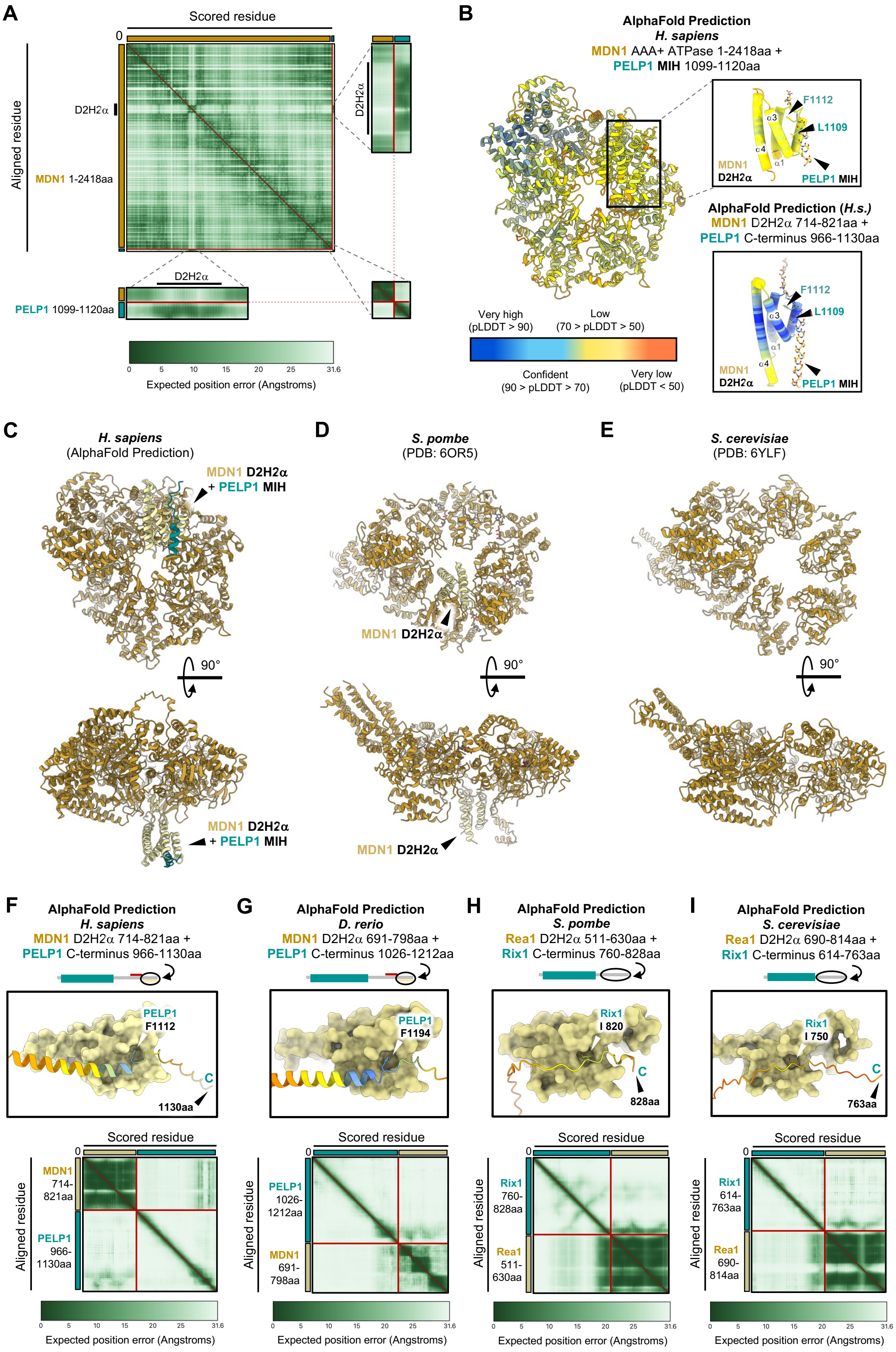

A
Scored residue
Aligned residue
D2H2α
MDN1 1-2418aa
PELP1 1099-1120aa
Expected position error (Angstroms)
B
AlphaFold Prediction
H. sapiens
MDN1 AAA+ ATPase 1-2418aa +
PELP1 MIH 1099-1120aa
F1112
L1109
MDN1 D2H2α
PELP1 MIH
AlphaFold Prediction (H.s.)
MDN1 D2H2α 714-821aa +
PELP1 C-terminus 966-1130aa
Very high (pLDDT > 90)
Low (70 > pLDDT > 50)
Confident (90 > pLDDT > 70)
Very low (pLDDT < 50)
C
H. sapiens
(AlphaFold Prediction)
MDN1 D2H2α + PELP1 MIH
90°
D
S. pombe
(PDB: 6OR5)
MDN1 D2H2α
E
S. cerevisiae
(PDB: 6YLF)
F
AlphaFold Prediction
H. sapiens
MDN1 D2H2α 714-821aa +
PELP1 C-terminus 966-1130aa
PELP1 F1112
C
1130aa
G
AlphaFold Prediction
D. rerio
MDN1 D2H2α 691-798aa +
PELP1 C-terminus 1026-1212aa
PELP1 F1194
H
AlphaFold Prediction
S. pombe
Rea1 D2H2α 511-630aa +
Rix1 C-terminus 760-828aa
Rix1 I 820
828aa
I
AlphaFold Prediction
S. cerevisiae
Rea1 D2H2α 690-814aa +
Rix1 C-terminus 614-763aa
Rix1 I 750
763aa

Fig. S4. Structure predictions of the PELP1-MDN1 interface, related to Fig. 4 (A) Predicted alignment error (PAE) plots of the illustrated AlphaFold3(*41*) model of human PELP1-MDN1. Plots were generated with the PAE viewer webserver (*98*). (B) AlphaFold3 confidence-depicted model of the human PELP1-MDN1 interface between the PELP1 MIH (MDN1 interacting helix, zoomed images depicted with ball-and-stick atom chain) and the MDN1 D2H2α insert (zoomed images depicted with tubed helices). Models were generated using the indicated residues and are colored by confidence level. Both zoomed images are of the same PELP1-MDN1 interaction region, but from two different predictions including or excluding the entire MDN1 AAA-ATPase ring. Both predictions result in the same structural model but with differing confidence values. (C) Comparison of the AlphaFold3 model of human MDN1 AAA-ATPase ring bound to PELP with (D) the cryo-EM structure of isolated *S. pombe* Mdn1 (PDBID 6OR5 (*44*)) and (E) ribosome bound *S. cerevisiae Rea1* (PDBID 6YLF (*30*)). (F-I) AlphaFold3 structural predictions and PAE plots of the PELP1-MDN1 interface across several eukaryotic species.

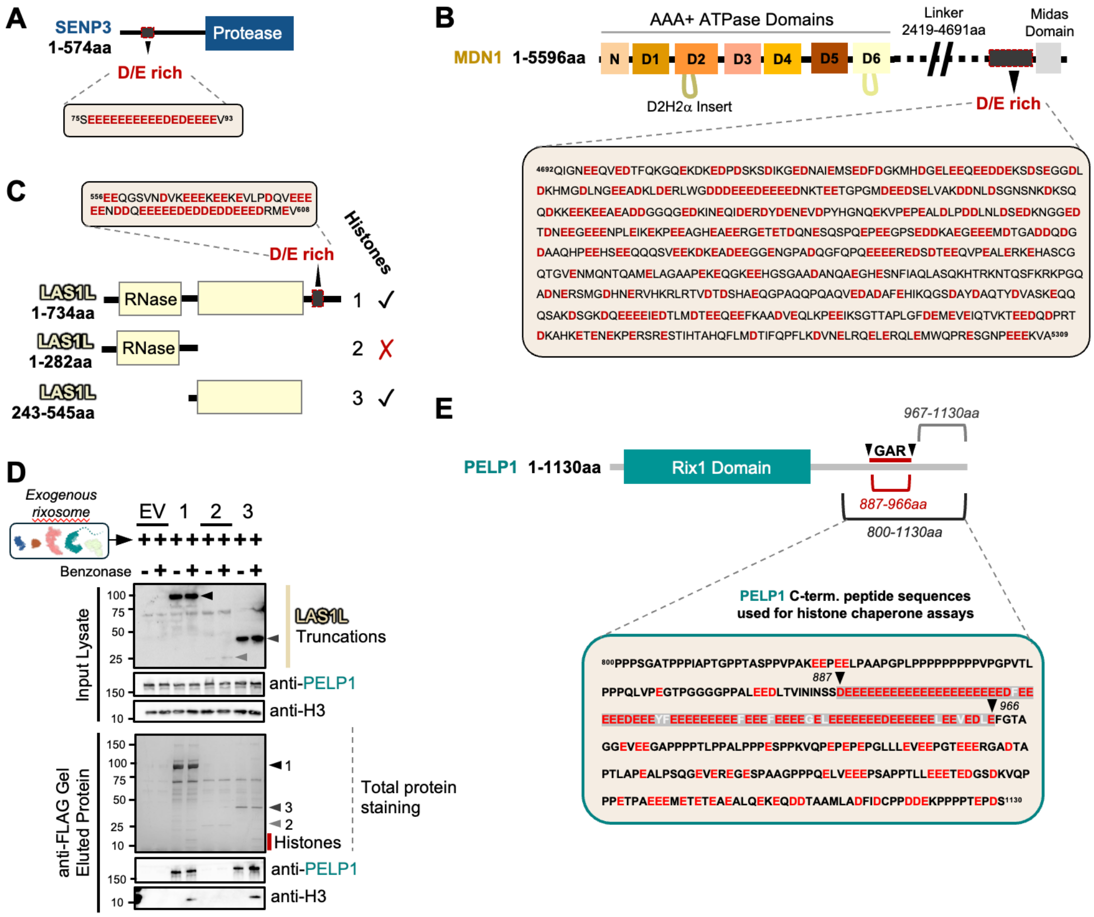

Fig. S5. Rixosome complex members with D/E rich regions (A-C) Cartoon schematic of human SENP3, MDN1, and LAS1L indicating D/E rich regions in these proteins. (D) When using LAS1L truncations as bait for rixosome reconstitutions, histones are only isolated when PELP1 is present and with Benzonase nuclease in the lysis buffer. (E) Cartoon schematic of human PELP1 with a focus on the D/E rich C-terminal IDR which contains the GAR. PELP1 IDR sequence regions used for histone chaperone assays in Fig. 5 are notated on schematic.

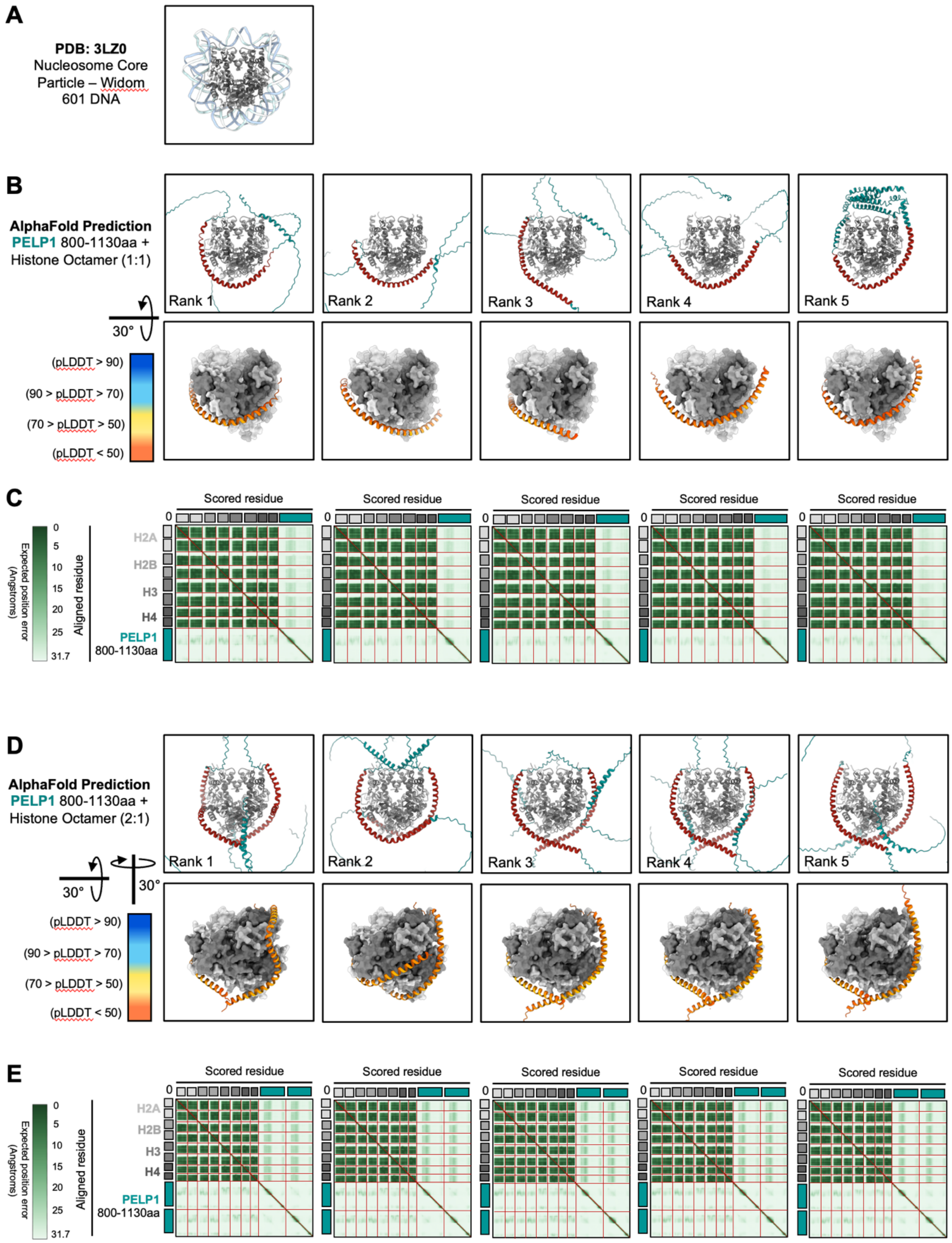

Fig. S6. Structure predictions of PELP1-histone interactions, related to Fig. 5 (A) Crystal structure of the nucleosome core particle with the Widom 601 DNA sequence (PDBID 3LZ0(*99*)). (B) All five ranked AlphaFold3 models of the PELP1 IDR (residues 800-1130) and

the histone octamer (grey) with a ratio of 1:1. The GAR region of the IDR is colored in red and the rest of the IDR is shown in teal. The bottom row depicts the confidence of the GAR-octamer interface. The histone octamer is depicted with surface representation and the GAR is depicted as colored ribbon structure. (C) PAE plots of all five ranked AlphaFold3 models shown in B. (D) All five ranked AlphaFold3 models of the PELP1 IDR (residues 800-1130) and the histone octamer (grey) with a ratio of 2:1, colors are the same as panel B. The bottom row depicts the confidence of the GAR-octamer interface same as in panel B. (E) PAE plots of all five ranked AlphaFold3 models shown in D.

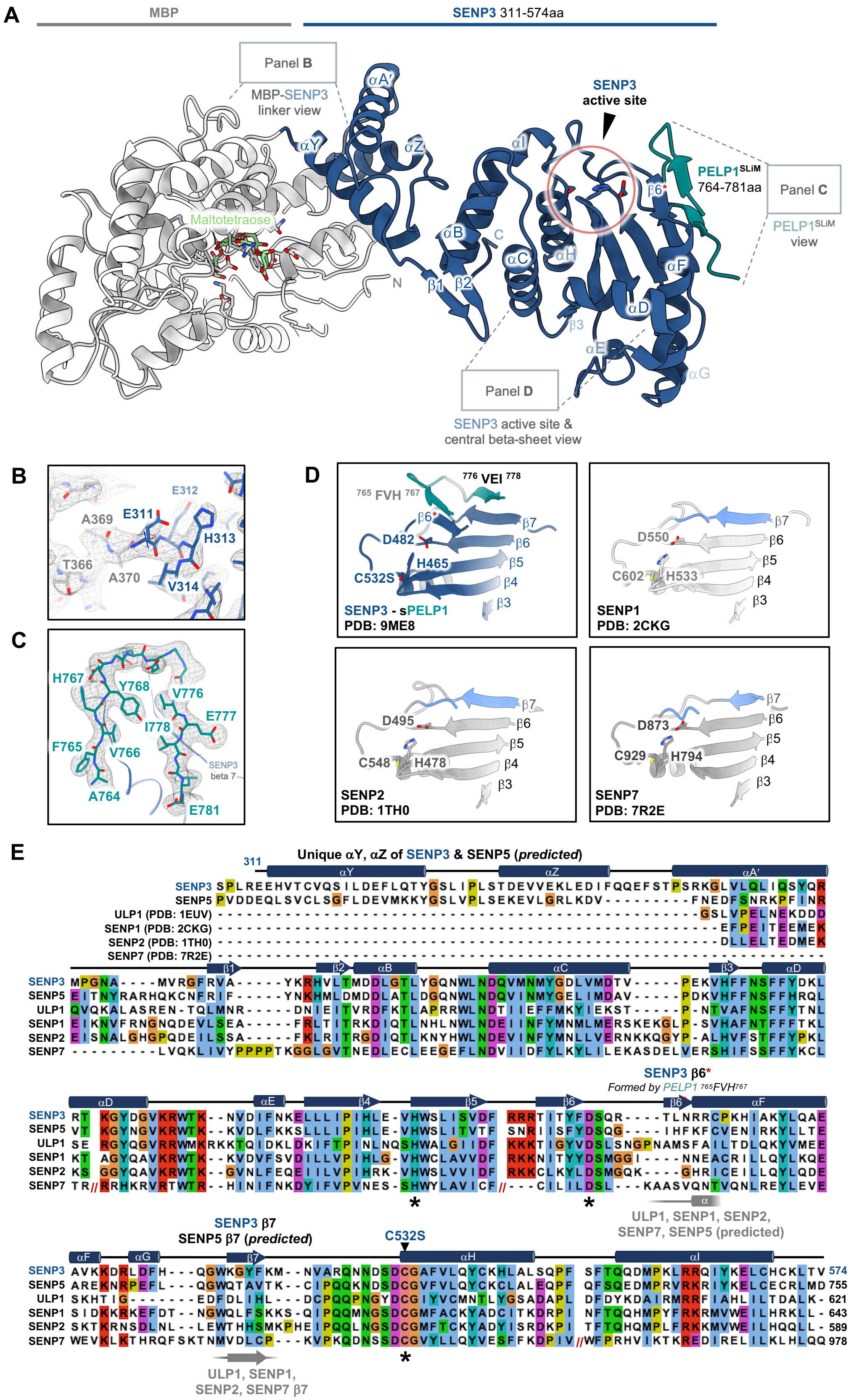

A
MBP
SENP3 311-574aa
Panel B
MBP-SENP3 linker view
αA'
αY
αZ
SENP3 active site
αI
PELP1SLiM 764-781aa
β6*
Panel C
PELP1SLiM view
Maltotetraose
αB
C
αC
αH
αF
N
β1
β2
αD
β3
αE
αG
Panel D
SENP3 active site & central beta-sheet view
B
E312
A369
E311
H313
T366
A370
V314
C
H767
Y768
V776
E777
I778
F765
V766
SENP3 beta 7
A764
E781
D
765 FVH 767
776 VEI 778
β6*
β7
D482
β6
H465
β5
C532S
β4
β3
SENP3 - sPELP1
PDB: 9ME8
D550
C602
H533
SENP1
PDB: 2CKG
D495
C548
H478
SENP2
PDB: 1TH0
D873
C929
H794
SENP7
PDB: 7R2E
E
Unique αY, αZ of SENP3 & SENP5 (predicted)
311
SENP3 SPLREEHVTCVQSILDEFLQTYGSLIPLSTDEVVEKLEDIFQQEFSTPSRKGLVLQLIQSYQR
SENP5 PVDDEQLSVCLSGFLDEVMKKYGSLVPLSEKEVLGRLKDV---------FNEDFSNRKPFINR
ULP1 (PDB: 1EUV)
SENP1 (PDB: 2CKG)
SENP2 (PDB: 1TH0)
SENP7 (PDB: 7R2E)
SENP3 β6*
Formed by PELP1 765FVH767
C532S
SENP3 β7
SENP5 β7 (predicted)
ULP1, SENP1, SENP2, SENP7, SENP5 (predicted)
ULP1, SENP1, SENP2, SENP7 β7
574
755
621
643
589
978

[illegible]

Fig. S7. X-ray structure and density of human MBP fused SENP3 protease domain bound to PELP1^{SLiM}, related to Fig. 6. (A) Global crystal structure of the MBP-SENP3/PELP1 complex. The fixed-arm MBP fusion is colored in grey and is bound to maltotetraose (green sticks). The catalytic domain of SENP3 is colored in dark blue and the PELP^{SLiM} is colored in teal. (B) Zoomed in view of the fixed-arm fusion between MBP and SENP3 with a composite omit map (σ contour level = 0.152 e / Å^3) overlaid. (C) Zoom in view of the PELP^{SLiM} with the composite omit map (σ contour level = 0.152 e / Å^3) overlaid in grey mesh. (D) Comparison of the central mixed β-sheet from SENP3 with structures of SENP1 (PDBID 2CKG (*100*)), SENP2 (PDBID 1TH0 (*101*)), and SENP7 (PDBID: 7R2E (*102*)). β7 in SENP1, SENP2, and SENP7 is colored in blue to indicate the structurally analogous region to SENP3 that coordinates PELP^{SLiM} binding. (E) Multiple sequence alignment of catalytic SUMO protease domain (with secondary structure indicated above) from human SENP3, human SENP5 (based on AlphaFold3 prediction), *S. cerevisiae* ULP1 (PDBID: 1EUV (*103*)), human SENP2 (PDBID 1TH0 (*101*)), and human SENP7 (PDBID: 7R2E (*102*)).αA′ of SENP3 is sequence aligned with αA of previously determined SENP structures but is not structurally equivalent.

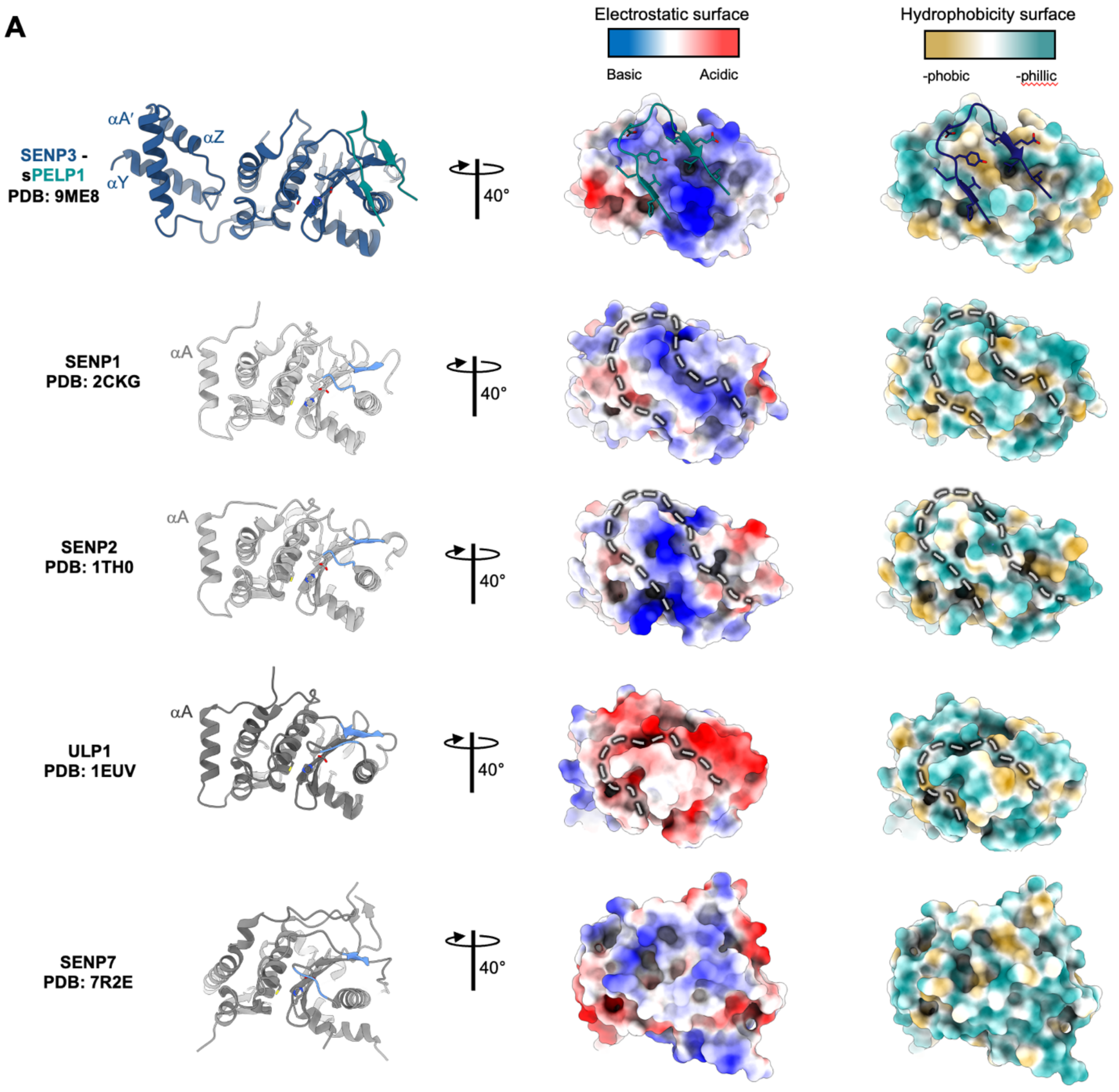

Fig. S8. Structural Comparison of SENP family members, related to Fig. 6 and 7. Comparison of published SENP structures. Each structure is shown in cartoon format (left) and surface format colored by electrostatics (middle) and hydrophobicity (right) (calculated and rendered by ChimeraX-1.8 (*104*)). β7 in SENP1, SENP2, ULP1, and SENP7 is indicated in blue. The dashed line on the surface of SENP1, SENP2, and ULP1 indicate a potential pocket which could accommodate a PELP1-like SLiM motif.

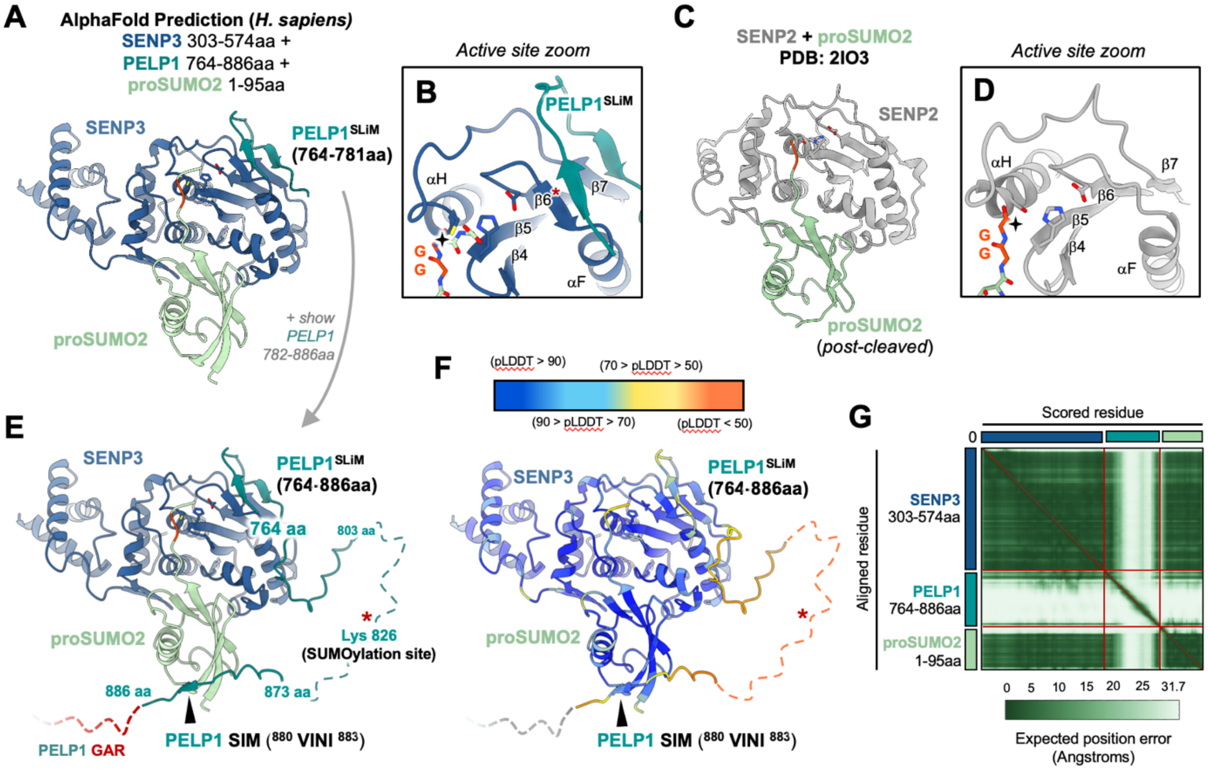

Fig. S9. Structural prediction of the PELP1-SENP3-SUMO2 complex. (A) AlphaFold3 structure prediction of SENP3 (blue) with PELP1 (teal) and proSUMO2 (green). (B) Zoomed in view of the active site from B. (C) Crystal structure of human SENP2 (grey) bound to a cleaved proSUMO2 (green) (PDBID 2IO3 (*105*)). (D) Active site Zoomed from D. (E) AlphaFold3 structure prediction of SENP3 (blue) with PELP1 (teal) and proSUMO2 (green) containing the SIM motif from PELP1. (F) Confidence of model in F. (G) PAE plot of the model from E.

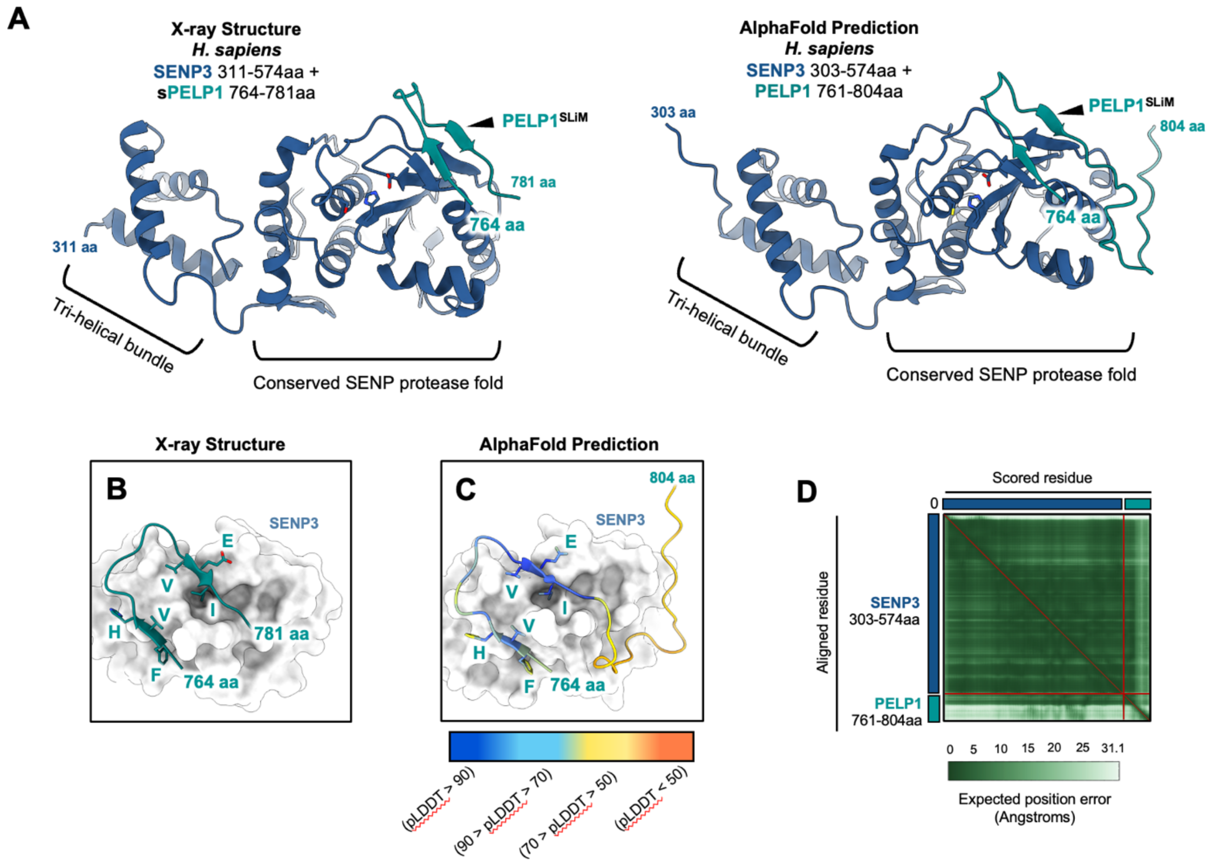

Fig. S10. Comparison of the experimental and AlphaFold3 structures of SENP3-PELP^{SLiM}. (A) Side-by-side comparison of the X-ray crystal structure of SENP3-PELP^{SLiM} with the AlphaFold3 model. (B) Zoomed in view of the SENP3-PELP^{SLiM} interface from the crystal structure. (C) Zoomed in view of the SENP3-PELP^{SLiM} interface from the AlphaFold3 model, with the SLiM colored by confidence. (D) PAE plot of the SENP3-PELP^{SLiM} AlphaFold3 model.

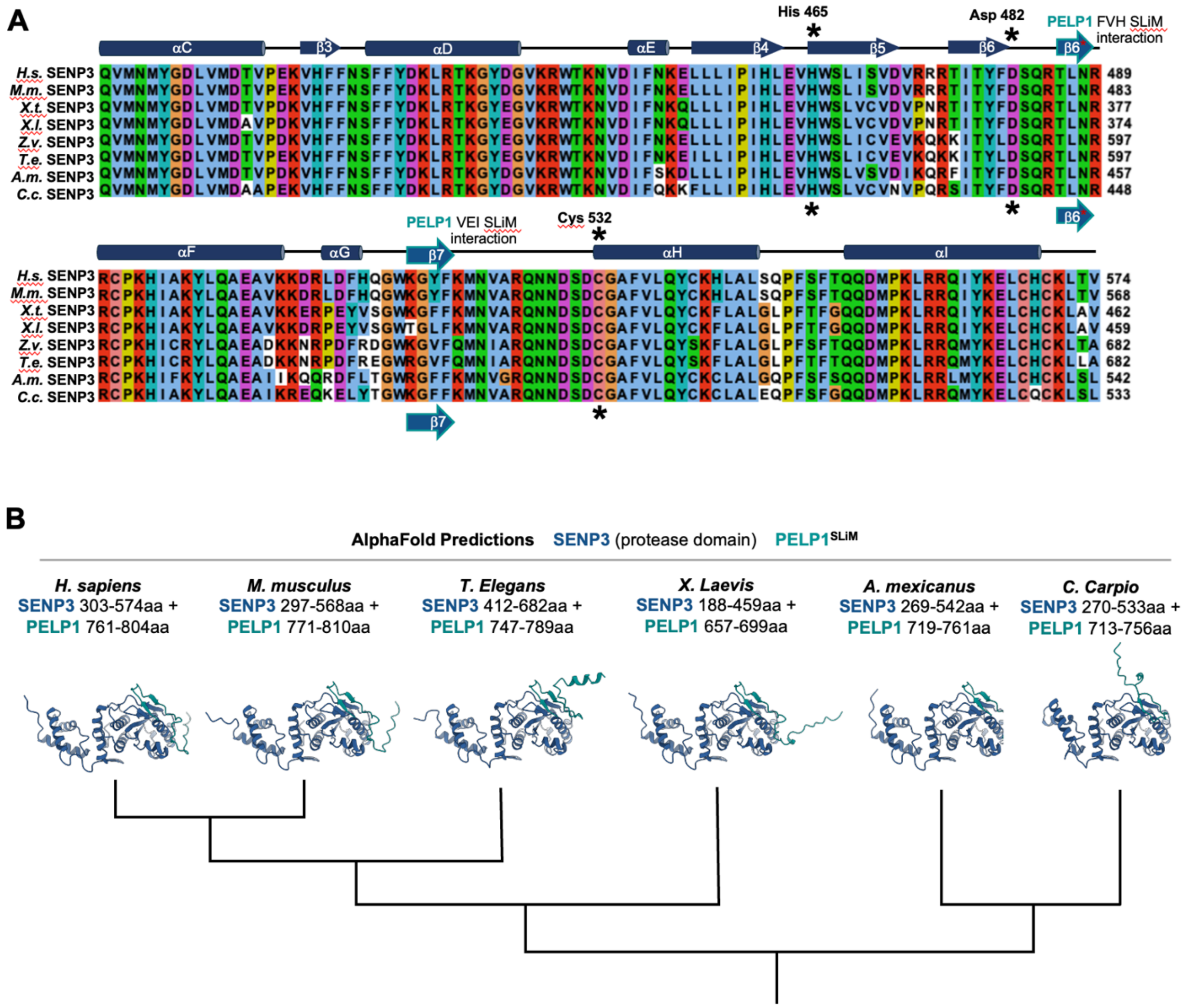

Fig. S11. The PELP1-SENP3 interface is well conserved across vertebrates, related to Fig. 6 and 7. (A) Multiple sequence alignment of SENP3 homologues across vertebrates, highlighting the interaction regions of PELP1 FVH and VEI from the SLiM. Abbreviations include: *Homo sapiens (Hs), Mus musculus (Mm), Xenopus tropicalis (Xt), Xenopus laevis (Xl), Zootoca vivipara (Zv), Thamnophis elegans (Te), Astyanax mexicanus (Am), Cyprinus carpio (Cc).* (B) Phylogenetic tree of AlphaFold3 predictions, showing the SENP3 and PELP1 association across high-to-low order vertebrate species.

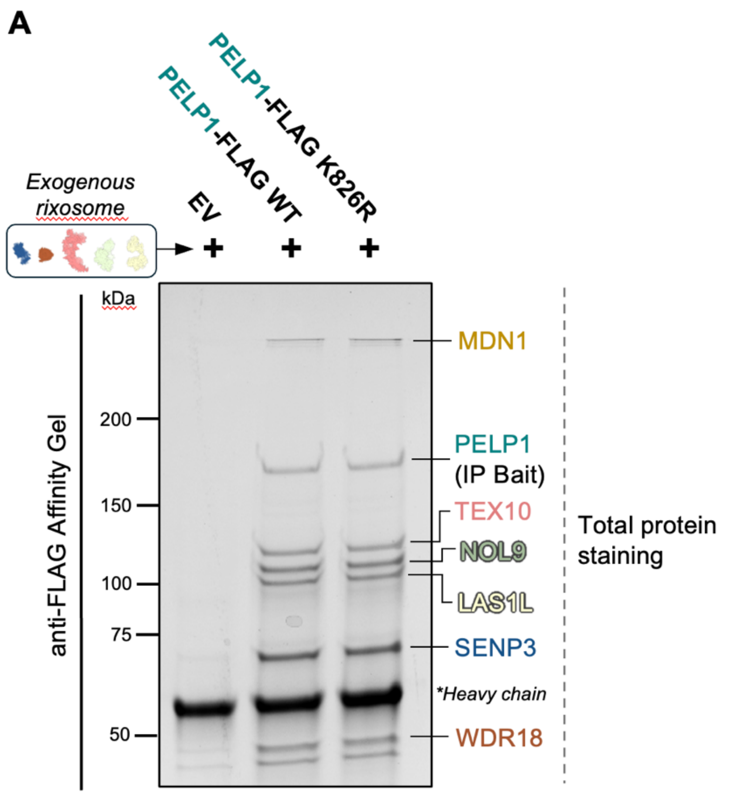

Fig. S12. PELP1 K826 SUMOylation status does not disrupt exogenous rixosome assembly. (A) SDS-PAGE and total protein stain of co-purified exogenous rixosome complexes with PELP1-FLAG WT or K826R mutant as bait protein. Rixosome members are labeled.

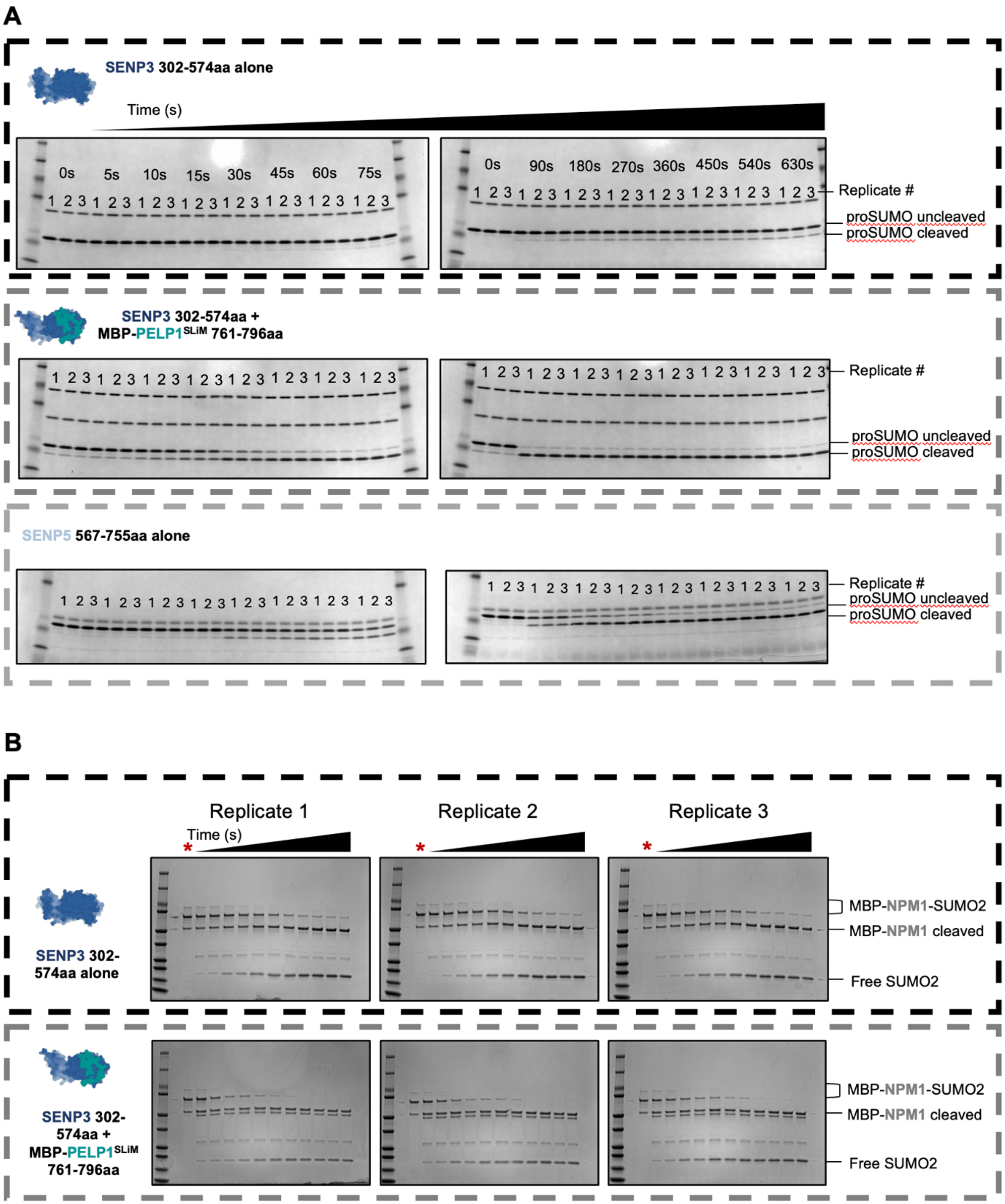

Fig. S13. SENP3 endopeptiase and isopeptidase assay gels, related to Fig. 7E and Fig. 8C. (A) Raw gels from endopeptidase time course assays in Fig. 7E. (B) Raw gels from isopeptidase time course assays in Fig. 8C.

Table S1: Plasmids used in this study

**Plasmid**	Source/Reference
Hs PELP1 WT 1-1130aa, pcDNA3.1-cFLAG	(*4*)
Hs PELP1 WT 1-1130aa, pcDNA3.1-cHA	This study
Hs PELP1 1-642aa, pcDNA3.1-cFLAG	(*4*)
Hs PELP1 1-801aa, pcDNA3.1-cFLAG	(*4*)
Hs PELP1 1-966aa, pcDNA3.1-cFLAG	(*4*)
Hs PELP1 del. 887-964aa, pcDNA3.1-cFLAG	This study
Hs PELP1 800-1130aa, pcDNA3.1-nFLAG	This study
Hs PELP1 643-1130aa, pcDNA3.1-nFLAG	(*4*)
Hs PELP1 del. 765-791aa, pcDNA3.1-cFLAG	This study
Hs PELP1 776PGP778, pcDNA3.1-cFLAG	This study
Hs PELP1 765GPG767, pcDNA3.1-cFLAG	This study
Hs PELP1 765GPG767 & 776PGP778, pcDNA3.1-cFLAG	This study
Hs PELP1 del. 1099-1120aa, pcDNA3.1-cFLAG	This study
Hs PELP1 F1112K, pcDNA3.1-cFLAG	This study
Hs PELP1 967-1130aa, pcDNA3.1-nFLAG	This study
Hs PELP1 967-1130aa F1112K, pcDNA3.1-nFLAG	This study
Hs SENP3 WT 1-574aa, pcDNA3.1-nMYC	(*4*)
Hs SENP3 WT 1-574aa, pcDNA3.1-nFLAG	This study
Hs SENP3 1-302aa, pcDNA3.1-nFLAG	This study
Hs SENP3 303-574aa, pcDNA3.1-nFLAG	This study
Hs WDR18 WT 1-432aa, pcDNA3.1-nHA	(*4*)
Hs TEX10 WT 1-929aa, pLexM-cGFP	(*4*)
Hs TEX10 WT 1-929aa, pLexM-nGFP	This study
Hs TEX10 46-929, pLexM-nGFP	This study
Hs TEX10 46-627, pLexM-nGFP	This study
Hs TEX10 46-375, pLexM-nGFP	This study
Hs TEX10 627-929aa, pLexM-nGFP	This study
Hs LAS1L WT 1-734aa, pCAG-OSF (nFLAG)	This study
Hs LAS1L WT 1-734aa, pcDNA3.1-nMYC	This study

Hs NOL9 WT 1-701aa, pLexM-nGFP	This study
Hs NOL9 WT 1-701aa, pcDNA3.1-nMYC	This study
Hs SUMO2 WT 1-93aa, pcDNA3.1-nHA	Addgene, Cat# 48967
Hs SUMO1 WT 1-97aa, pcDNA3.1-nHA	Addgene, Cat# 48966
Human PELP1 800-1130aa, pET11a His-MBP-PELP1 800-1130aa	Genscript (This study)
Human PELP1 887-966aa, pET11a His-MBP-PELP1 887-966aa	Genscript (This study)
Human PELP1 967-1130aa, pET11a His-MBP-PELP1 967-1130aa	Genscript (This study)
Human PELP1 761-796aa, pET11a His-MBP-PELP1 761-796aa	Genscript (This study)
Human SENP3 303-574aa, pET11a His-SENP3 303-574aa	Genscript (This study)
Human SENP3 303-574aa C532A, pET11a His-SENP3 303-574aa C532A	Genscript (This study)
Human SENP5 367-755aa, pET28a nHis-Hs SENP5 567-755aa	Addgene, Cat# 16358
Human proSUMO1, pET28a proSUMO1 1-101aa cHis	Addgene, Cat# 25101
Human proSUMO2, pET28a proSUMO2 1-95aa cHis	Addgene, Cat# 25102
Human proSUMO3, pET28a proSUMO3 1-105aa cHis	Addgene, Cat# 25103
Human pSUMO2 (6xHis-SUMO2, UBC9, SAE1, SAE2), pCDFDuet-1	Addgene, Cat# 52259
Human NPM1 240-294aa, pET11a MBP-FLAG-NPM1 240-294aa	Genscript (This study)
Human SENP3 311-574aa C532S + MBP fixed-arm fusion, pMALX nHis-MBP-SENP3 311-574aa	This study
human H2A1, pST50Trc3-hH2A.D	(*80*)
human H2B1C, pST50Trc4-hH2B.C	(*80*)
human H3.2, pST50Tr-hH3.2	(*80*)
human H4 (same protein sequence as Xenopus H4), pET3a-xH4	(*79*)
Human ncPRC1.4, pST44-SUMOHSTNhRING1B-hBMI1-hRYBP	This study

REFERENCES AND NOTES

1. H. Zhou, C. B. Stein, T. A. Shafiq, G. Shipkovenska, M. Kalocsay, J. A. Paulo, J. Zhang, Z. Luo, S. P. Gygi, K. Adelman, D. Moazed, Rixosomal RNA degradation contributes to silencing of Polycomb target genes. *Nature* **604**, 167–174 (2022).

2. C. D. Castle, E. K. Cassimere, C. Denicourt, LAS1L interacts with the mammalian Rix1 complex to regulate ribosome biogenesis. *Mol. Biol. Cell* **23**, 716–728 (2012).

3. A. Vanden Broeck, S. Klinge, Principles of human pre-60*S* biogenesis. *Science* **381**, eadh3892 (2023).

4. J. Gordon, F. L. Chapus, E. G. Viverette, J. G. Williams, L. J. Deterding, J. M. Krahn, M. J. Borgnia, J. Rodriguez, A. J. Warren, R. E. Stanley, Cryo-EM reveals the architecture of the PELP1-WDR18 molecular scaffold. *Nat. Commun.* **13**, 6783 (2022).

5. G. R. Sareddy, R. K. Vadlamudi, PELP1: Structure, biological function and clinical significance. *Gene* **585**, 128–134 (2016).

6. V. K. Gonugunta, L. Miao, G. R. Sareddy, P. Ravindranathan, R. Vadlamudi, G. V. Raj, The social network of PELP1 and its implications in breast and prostate cancers. *Endocr. Relat. Cancer* **21**, T79–T86 (2014).

7. A. Vanden Broeck, S. Klinge, Eukaryotic ribosome assembly. *Annu. Rev. Biochem.* **93**, 189–210 (2024).

8. M. N. Frazier, M. C. Pillon, S. Kocaman, J. Gordon, R. E. Stanley, Structural overview of macromolecular machines involved in ribosome biogenesis. *Curr. Opin. Struct. Biol.* **67**, 51–60 (2021).

9. S. Klinge, J. L. Woolford Jr., Ribosome assembly coming into focus. *Nat. Rev. Mol. Cell Biol.* **20**, 116–131 (2019).

10. E. Finkbeiner, M. Haindl, S. Muller, The SUMO system controls nucleolar partitioning of a novel mammalian ribosome biogenesis complex. *EMBO J.* **30**, 1067–1078 (2011).

11. L. Gasse, D. Flemming, E. Hurt, Coordinated ribosomal ITS2 RNA processing by the Las1 complex integrating endonuclease, polynucleotide kinase, and exonuclease activities. *Mol. Cell* **60**, 808–815 (2015).

12. M. C. Pillon, A. L. Hsu, J. M. Krahn, J. G. Williams, K. H. Goslen, M. Sobhany, M. J. Borgnia, R. E. Stanley, Cryo-EM reveals active site coordination within a multienzyme pre-rRNA processing complex. *Nat. Struct. Mol. Biol.* **26**, 830–839 (2019).

13. J. Gordon, M. C. Pillon, R. E. Stanley, Nol9 is a spatial regulator for the human ITS2 pre-rRNA endonuclease-kinase complex. *J. Mol. Biol.* **431**, 3771–3786 (2019).

14. M. C. Pillon, M. Sobhany, R. E. Stanley, Characterization of the molecular crosstalk within the essential Grc3/Las1 pre-rRNA processing complex. *RNA* **24**, 721–738 (2018).

15. M. C. Pillon, M. Sobhany, M. J. Borgnia, J. G. Williams, R. E. Stanley, Grc3 programs the essential endoribonuclease Las1 for specific RNA cleavage. *Proc. Natl. Acad. Sci. U.S.A.* **114**, E5530–E5538 (2017).

16. M. Thoms, B. Lau, J. Cheng, L. Fromm, T. Denk, N. Kellner, D. Flemming, P. Fischer, L. Falquet, O. Berninghausen, R. Beckmann, E. Hurt, Structural insights into coordinating 5S RNP rotation with ITS2 pre-RNA processing during ribosome formation. *EMBO Rep.* **24**, e57984 (2023).

17. M. Uckelmann, C. Davidovich, An added layer of repression for human genes. *Nature* **604**, 41–42 (2022).

18. N. P. Blackledge, R. J. Klose, The molecular principles of gene regulation by Polycomb repressive complexes. *Nat. Rev. Mol. Cell Biol.* **22**, 815–833 (2021).

19. S. Tamburri, S. Rustichelli, S. Amato, D. Pasini, Navigating the complexity of Polycomb repression: Enzymatic cores and regulatory modules. *Mol. Cell* **84**, 3381–3405 (2024).

20. G. Shipkovenska, A. Durango, M. Kalocsay, S. P. Gygi, D. Moazed, A conserved RNA degradation complex required for spreading and epigenetic inheritance of heterochromatin. *eLife* **9**, e54341 (2020).

21. S. Holla, J. Dhakshnamoorthy, H. D. Folco, V. Balachandran, H. Xiao, L.-L. Sun, D. Wheeler, M. Zofall, S. I. S. Grewal, Positioning heterochromatin at the nuclear periphery suppresses histone turnover to promote epigenetic inheritance. *Cell* **180**, 150–164.e15 (2020).

22. L. A. Claessens, A. C. O. Vertegaal, SUMO proteases: From cellular functions to disease. *Trends Cell Biol.* **34**, 901–912 (2024).

23. J. Dönig, H. Mende, J. Davila Gallesio, K. Wagner, P. Hotz, K. Schunck, T. Piller, S. Hölper, S. Uhan, M. Kaulich, M. Wirth, U. Keller, G. Tascher, K. E. Bohnsack, S. Müller, Characterization of nucleolar SUMO isopeptidases unveils a general p53-independent checkpoint of impaired ribosome biogenesis. *Nat. Commun.* **14**, 8121 (2023).

24. N. Raman, E. Weir, S. Müller, The AAA ATPase MDN1 acts as a SUMO-targeted regulator in mammalian pre-ribosome remodeling. *Mol. Cell* **64**, 607–615 (2016).

25. Y. Li, Y. Yang, R. C. Sears, M.-S. Dai, X.-X. Sun, USP36 SUMOylates Las1L and promotes its function in pre-ribosomal RNA ITS2 processing. *Cancer Res. Commun.* **4**, 2835–2845 (2024).

26. M. Haindl, T. Harasim, D. Eick, S. Müller, The nucleolar SUMO-specific protease SENP3 reverses SUMO modification of nucleophosmin and is required for rRNA processing. *EMBO Rep.* **9**, 273–279 (2008).

27. H. Zhou, W. Feng, J. Yu, T. A. Shafiq, J. A. Paulo, J. Zhang, Z. Luo, S. P. Gygi, D. Moazed, SENP3 and USP7 regulate Polycomb-rixosome interactions and silencing functions. *Cell Rep.* **42**, 112339 (2023).

28. X. Long, B. Zhao, W. Lu, X. Chen, X. Yang, J. Huang, Y. Zhang, S. An, Y. Qin, Z. Xing, Y. Shen, H. Wu, Y. Qi, The critical roles of the SUMO-specific protease SENP3 in human diseases and clinical implications. *Front. Physiol.* **11**, 558220 (2020).

29. J. Huang, L. Tong, Molecular insights into the overall architecture of human rixosome. *Nat. Commun.* **16**, 3288 (2025).

30. L. Kater, V. Mitterer, M. Thoms, J. Cheng, O. Berninghausen, R. Beckmann, E. Hurt, Construction of the central protuberance and L1 stalk during 60S subunit biogenesis. *Mol. Cell* **79**, 615–628.e5 (2020).

31. Z. Liu, K. A. Altwegg, J. Liu, S. T. Weintraub, Y. Chen, Z. Lai, G. R. Sareddy, S. Viswanadhapalli, R. K. Vadlamudi, Global genomic and proteomic analysis identified critical pathways modulated by proto-oncogene PELP1 in TNBC. *Cancers (Basel)* **14**, 930 (2022).

32. P. Fanis, N. Gillemans, A. Aghajanirefah, F. Pourfarzad, J. Demmers, F. Esteghamat, R. K. Vadlamudi, F. Grosveld, S. Philipsen, T. B. van Dijk, Five friends of methylated chromatin target of protein-arginine-methyltransferase[prmt]-1 (chtop), a complex linking arginine methylation to desumoylation. *Mol. Cell. Proteomics* **11**, 1263–1273 (2012).

33. K. A. Altwegg, U. P. Pratap, Z. Liu, J. Liu, J. R. Sanchez, X. Yang, B. Ebrahimi, D. M. Panneerdoss, X. Li, G. R. Sareddy, S. Viswanadhapalli, M. K. Rao, R. K. Vadlamudi, Targeting PELP1 oncogenic signaling in TNBC with the small molecule inhibitor SMIP34. *Breast Cancer Res. Treat.* **200**, 151–162 (2023).

34. A. J. LaPeruta, J. Micic, J. L. Woolford Jr., Additional principles that govern the release of pre-ribosomes from the nucleolus into the nucleoplasm in yeast. *Nucleic Acids Res.* **51**, 10867–10883 (2023).

35. K. Cermakova, H. C. Hodges, Interaction modules that impart specificity to disordered protein. *Trends Biochem. Sci.* **48**, 477–490 (2023).

36. A. S. Holehouse, B. B. Kragelund, The molecular basis for cellular function of intrinsically disordered protein regions. *Nat. Rev. Mol. Cell Biol.* **25**, 187–211 (2024).

37. B. S. McConnell, M. W. Parker, Protein intrinsically disordered regions have a non-random, modular architecture. *Bioinformatics* **39**, btad732 (2023).

38. B. Mateos, C. Conrad-Billroth, M. Schiavina, A. Beier, G. Kontaxis, R. Konrat, I. C. Felli, R. Pierattelli, The ambivalent role of proline residues in an intrinsically disordered protein: From disorder promoters to compaction facilitators. *J. Mol. Biol.* **432**, 3093–3111 (2020).

39. Y. B. Choi, J. K. Ko, J. Shin, The transcriptional corepressor, PELP1, recruits HDAC2 and masks histones using two separate domains. *J. Biol. Chem.* **279**, 50930–50941 (2004).

40. M. Prattes, Y.-H. Lo, H. Bergler, R. E. Stanley, Shaping the nascent ribosome: AAA-ATPases in eukaryotic ribosome biogenesis. *Biomolecules* **9**, 715 (2019).

41. J. Abramson, J. Adler, J. Dunger, R. Evans, T. Green, A. Pritzel, O. Ronneberger, L. Willmore, A. J. Ballard, J. Bambrick, S. W. Bodenstein, D. A. Evans, C.-C. Hung, M. O'Neill, D. Reiman, K. Tunyasuvunakool, Z. Wu, A. Žemgulytė, E. Arvaniti, C. Beattie, O. Bertolli, A. Bridgland, A. Cherepanov, M. Congreve, A. I. Cowen-Rivers, A. Cowie, M. Figurnov, F. B. Fuchs, H. Gladman, R. Jain, Y. A. Khan, C. M. R. Low, K. Perlin, A. Potapenko, P. Savy, S. Singh, A. Stecula, A. Thillaisundaram, C. Tong, S. Yakneen, E. D. Zhong, M. Zielinski, A. Žídek, V. Bapst, P. Kohli, M. Jaderberg, D. Hassabis, J. M. Jumper, Accurate structure prediction of biomolecular interactions with AlphaFold 3. *Nature* **630**, 493–500 (2024).

42. C. Barrio-Garcia, M. Thoms, D. Flemming, L. Kater, O. Berninghausen, J. Baßler, R. Beckmann, E. Hurt, Architecture of the Rix1-Rea1 checkpoint machinery during pre-60S-ribosome remodeling. *Nat. Struct. Mol. Biol.* **23**, 37–44 (2016).

43. P. Sosnowski, L. Urnavicius, A. Boland, R. Fagiewicz, J. Busselez, G. Papai, H. Schmidt, The CryoEM structure of the *Saccharomyces cerevisiae* ribosome maturation factor Rea1. *eLife* **7**, e39163 (2018).

44. Z. Chen, H. Suzuki, Y. Kobayashi, A. C. Wang, F. DiMaio, S. A. Kawashima, T. Walz, T. M. Kapoor, Structural insights into Mdn1, an essential AAA protein required for ribosome biogenesis. *Cell* **175**, 822–834.e18 (2018).

45. J. Busselez, G. Koenig, C. Dominique, T. Klos, D. Velayudhan, P. Sosnowski, N. Marechal, C. Crucifix, H. Gizardin-Fredon, S. Cianferani, B. Albert, Y. Henry, A. K. Henras, H. Schmidt,

Remodelling of Rea1 linker domain drives the removal of assembly factors from pre-ribosomal particles. *Nat. Commun.* **15**, 10309 (2024).

46. N. Ogasawara, K. Kasahara, R. Iwai, T. Takahashi, Unfolding of α-helical 20-residue poly-glutamic acid analyzed by multiple runs of canonical molecular dynamics simulations. *PeerJ* **6**, e4769 (2018).

47. M. R. King, K. M. Ruff, A. Z. Lin, A. Pant, M. Farag, J. M. Lalmansingh, T. Wu, M. J. Fossat, W. Ouyang, M. D. Lew, E. Lundberg, M. D. Vahey, R. V. Pappu, Macromolecular condensation organizes nucleolar sub-phases to set up a pH gradient. *Cell* **187**, 1889–1906.e24 (2024).

48. C. Warren, D. Shechter, Fly fishing for histones: Catch and release by histone chaperone intrinsically disordered regions and acidic stretches. *J. Mol. Biol.* **429**, 2401–2426 (2017).

49. C. M. Hammond, C. B. Strømme, H. Huang, D. J. Patel, A. Groth, Histone chaperone networks shaping chromatin function. *Nat. Rev. Mol. Cell Biol.* **18**, 141–158 (2017).

50. S. S. Nair, B. C. Nair, V. Cortez, D. Chakravarty, E. Metzger, R. Schüle, D. W. Brann, R. R. Tekmal, R. K. Vadlamudi, PELP1 is a reader of histone H3 methylation that facilitates oestrogen receptor-α target gene activation by regulating lysine demethylase 1 specificity. *EMBO Rep.* **11**, 438–444 (2010).

51. M. Mann, V. Cortez, R. Vadlamudi, PELP1 oncogenic functions involve CARM1 regulation. *Carcinogenesis* **34**, 1468–1475 (2013).

52. K. Kashiwaya, H. Nakagawa, M. Hosokawa, Y. Mochizuki, K. Ueda, L. Piao, S. Chung, R. Hamamoto, H. Eguchi, H. Ohigashi, O. Ishikawa, C. Janke, Y. Shinomura, Y. Nakamura, Involvement of the tubulin tyrosine ligase-like family member 4 polyglutamylase in PELP1 polyglutamylation and chromatin remodeling in pancreatic cancer cells. *Cancer Res.* **70**, 4024–4033 (2010).

53. E. L. Huttlin, R. J. Bruckner, J. Navarrete-Perea, J. R. Cannon, K. Baltier, F. Gebreab, M. P. Gygi, A. Thornock, G. Zarraga, S. Tam, J. Szpyt, B. M. Gassaway, A. Panov, H. Parzen, S. Fu, A. Golbazi, E. Maenpaa, K. Stricker, S. Guha Thakurta, T. Zhang, R. Rad, J. Pan, D. P.

Nusinow, J. A. Paulo, D. K. Schweppe, L. P. Vaites, J. W. Harper, S. P. Gygi, Dual proteome-scale networks reveal cell-specific remodeling of the human interactome. *Cell* **184**, 3022–3040.e28 (2021).

54. A. Skrajna, D. Goldfarb, K. M. Kedziora, E. M. Cousins, G. D. Grant, C. J. Spangler, E. H. Barbour, X. Yan, N. A. Hathaway, N. G. Brown, J. G. Cook, M. B. Major, R. K. McGinty, Comprehensive nucleosome interactome screen establishes fundamental principles of nucleosome binding. *Nucleic Acids Res.* **48**, 9415–9432 (2020).

55. A. Stein, J. P. Whitlock Jr., M. Bina, Acidic polypeptides can assemble both histones and chromatin in vitro at physiological ionic strength. *Proc. Natl. Acad. Sci. U.S.A.* **76**, 5000–5004 (1979).

56. I. Corbeski, X. Guo, B. V. Eckhardt, D. Fasci, W. Wiegant, M. A. Graewert, K. Vreeken, H. Wienk, D. I. Svergun, A. J. R. Heck, H. van Attikum, R. Boelens, T. K. Sixma, F. Mattiroli, H. van Ingen, Chaperoning of the histone octamer by the acidic domain of DNA repair factor APLF. *Sci. Adv.* **8**, eabo0517 (2022).

57. A. Nayak, S. Müller, SUMO-specific proteases/isopeptidases: SENPs and beyond. *Genome Biol.* **15**, 422 (2014).

58. A. C. Y. Foo, P. M. Thompson, S.-H. Chen, R. Jadi, B. Lupo, E. F. DeRose, S. Arora, V. C. Placentra, L. Premkumar, L. Perera, L. C. Pedersen, N. Martin, G. A. Mueller, The mosquito protein AEG12 displays both cytolytic and antiviral properties via a common lipid transfer mechanism. *Proc. Natl. Acad. Sci. U.S.A.* **118**, e2019251118 (2021).

59. L. Holm, A. Laiho, P. Törönen, M. Salgado, DALI shines a light on remote homologs: One hundred discoveries. *Protein Sci.* **32**, e4519 (2023).

60. M. Kumar, S. Michael, J. Alvarado-Valverde, A. Zeke, T. Lazar, J. Glavina, E. Nagy-Kanta, J. M. Donagh, Z. E. Kalman, S. Pascarelli, N. Palopoli, L. Dobson, C. F. Suarez, K. Van Roey, I. Krystkowiak, J. E. Griffin, A. Nagpal, R. Bhardwaj, F. Diella, B. Mészáros, K. Dean, N. E.

Davey, R. Pancsa, L. B. Chemes, T. J. Gibson, ELM – The eukaryotic linear motif resource – 2024 update. *Nucleic Acids Res.* **52**, D442–D455 (2024).

61. J. Mikolajczyk, M. Drag, M. Békés, J. T. Cao, Z. Ronai, G. S. Salvesen, Small ubiquitin-related modifier (SUMO)-specific proteases: Profiling the specificities and activities of human SENPs. *J. Biol. Chem.* **282**, 26217–26224 (2007).

62. A. V. Mendes, C. P. Grou, J. E. Azevedo, M. P. Pinto, Evaluation of the activity and substrate specificity of the human SENP family of SUMO proteases. *Biochim. Biophys. Acta* **1863**, 139–147 (2016).

63. J. R. Gareau, C. D. Lima, The SUMO pathway: Emerging mechanisms that shape specificity, conjugation and recognition. *Nat. Rev. Mol. Cell Biol.* **11**, 861–871 (2010).

64. N. Raman, A. Nayak, S. Muller, mTOR signaling regulates nucleolar targeting of the SUMO-specific isopeptidase SENP3. *Mol. Cell. Biol.* **34**, 4474–4484 (2014).

65. A. R. Weber, D. Schuermann, P. Schär, Versatile recombinant SUMOylation system for the production of SUMO-modified protein. *PLOS ONE* **9**, e102157 (2014).

66. X. Liu, Z. Liu, S.-W. Jang, Z. Ma, K. Shinmura, S. Kang, S. Dong, J. Chen, K. Fukasawa, K. Ye, Sumoylation of nucleophosmin/B23 regulates its subcellular localization, mediating cell proliferation and survival. *Proc. Natl. Acad. Sci. U.S.A.* **104**, 9679–9684 (2007).

67. C. G. Grummitt, F. M. Townsley, C. M. Johnson, A. J. Warren, M. Bycroft, Structural consequences of nucleophosmin mutations in acute myeloid leukemia. *J. Biol. Chem.* **283**, 23326–23332 (2008).

68. B. M. Lorton, C. Warren, H. Ilyas, P. Nandigrami, S. Hegde, S. Cahill, S. M. Lehman, J. Shabanowitz, D. F. Hunt, A. Fiser, D. Cowburn, D. Shechter, Glutamylation of Npm2 and Nap1 acidic disordered regions increases DNA mimicry and histone chaperone efficiency. *iScience* **27**, 109458 (2024).

69. C. Jeronimo, F. Robert, The histone chaperone FACT: A guardian of chromatin structure integrity. *Transcription* **13**, 16–38 (2022).

70. S. He, E. Valkov, S. Cheloufi, J. Murn, The nexus between RNA-binding proteins and their effectors. *Nat. Rev. Genet.* **24**, 276–294 (2023).

71. J. Lascorz, J. Codina-Fabra, D. Reverter, J. Torres-Rosell, SUMO-SIM interactions: From structure to biological functions. *Semin. Cell Dev. Biol.* **132**, 193–202 (2022).

72. F. Chen, H. Yan, C. Guo, H. Zhu, J. Yi, X. Sun, J. Yang, Assessment of SENP3-interacting proteins in hepatocytes treated with diethylnitrosamine by BioID assay. *Acta Biochim. Biophys. Sin. (Shanghai)* **53**, 1237–1246 (2021).

73. J. D. Graves, Y.-J. Lee, K. Liu, G. Li, F.-T. Lin, W.-C. Lin, E2F1 sumoylation as a protective cellular mechanism in oxidative stress response. *Proc. Natl. Acad. Sci. U.S.A.* **117**, 14958–14969 (2020).

74. Z. Hu, X.-L. Teng, T. Zhang, X. Yu, R. Ding, J. Yi, L. Deng, Z. Wang, Q. Zou, SENP3 senses oxidative stress to facilitate STING-dependent dendritic cell antitumor function. *Mol. Cell* **81**, 940–952.e5 (2021).

75. A. Zhao, L. Maple, J. Jiang, K. N. Myers, C. G. Jones, H. Gagg, C. McGarrity-Cottrell, O. Rominiyi, S. J. Collis, G. Wells, M. Rahman, S. J. Danson, D. Robinson, C. Smythe, C. Guo, SENP3-FIS1 axis promotes mitophagy and cell survival under hypoxia. *Cell Death Dis.* **15**, 881 (2024).

76. M. J. Schellenberg, R. M. Petrovich, C. C. Malone, R. S. Williams, Selectable high-yield recombinant protein production in human cells using a GFP/YFP nanobody affinity support. *Protein Sci.* **27**, 1083–1092 (2018).

77. N. A. Wesley, A. Skrajna, H. C. Simmons, G. R. Budziszewski, D. N. Azzam, A. P. Cesmat, R. K. McGinty, Time resolved-fluorescence resonance energy transfer platform for quantitative nucleosome binding and footprinting. *Protein Sci.* **31**, e4339 (2022).

78. S. Tan, R. C. Kern, W. Selleck, The pST44 polycistronic expression system for producing protein complexes in *Escherichia coli*. *Protein Expr. Purif.* **40**, 385–395 (2005).

79. K. Luger, T. J. Rechsteiner, T. J. Richmond, Preparation of nucleosome core particle from recombinant histones. *Methods Enzymol.* **304**, 3–19 (1999).

80. C. J. Anderson, M. R. Baird, A. Hsu, E. H. Barbour, Y. Koyama, M. J. Borgnia, R. K. McGinty, Structural basis for recognition of ubiquitylated nucleosome by Dot1L methyltransferase. *Cell Rep.* **26**, 1681–1690.e5 (2019).

81. L. J. McGuffin, K. Bryson, D. T. Jones, The PSIPRED protein structure prediction server. *Bioinformatics* **16**, 404–405 (2000).

82. C. Schneider, K. E. Bohnsack, Caught in the act – Visualizing ribonucleases during eukaryotic ribosome assembly. *Wiley Interdiscip. Rev. RNA* **14**, e1766 (2023).

83. C. Vonrhein, C. Flensburg, P. Keller, A. Sharff, O. Smart, W. Paciorek, T. Womack, G. Bricogne, Data processing and analysis with the autoPROC toolbox. *Acta Crystallogr. D Biol. Crystallogr.* **67**, 293–302 (2011).

84. P. H. Zwart, P. V. Afonine, R. W. Grosse-Kunstleve, L.-W. Hung, T. R. Ioerger, A. J. McCoy, E. McKee, N. W. Moriarty, R. J. Read, J. C. Sacchettini, N. K. Sauter, L. C. Storoni, T. C. Terwilliger, P. D. Adams, Automated structure solution with the PHENIX suite. *Methods Mol. Biol.* **426**, 419–435 (2008).

85. J. Jumper, R. Evans, A. Pritzel, T. Green, M. Figurnov, O. Ronneberger, K. Tunyasuvunakool, R. Bates, A. Žídek, A. Potapenko, A. Bridgland, C. Meyer, S. A. A. Kohl, A. J. Ballard, A. Cowie, B. Romera-Paredes, S. Nikolov, R. Jain, J. Adler, T. Back, S. Petersen, D. Reiman, E. Clancy, M. Zielinski, M. Steinegger, M. Pacholska, T. Berghammer, S. Bodenstein, D. Silver, O. Vinyals, A. W. Senior, K. Kavukcuoglu, P. Kohli, D. Hassabis, Highly accurate protein structure prediction with AlphaFold. *Nature* **596**, 583–589 (2021).

86. P. Emsley, K. Cowtan, Coot: Model-building tools for molecular graphics. *Acta Crystallogr. D Biol. Crystallogr.* **60**, 2126–2132 (2004).

87. P. Emsley, B. Lohkamp, W. G. Scott, K. Cowtan, Features and development of Coot. *Acta Crystallogr. D Biol. Crystallogr.* **66**, 486–501 (2010).

88. P. D. Adams, P. V. Afonine, G. Bunkóczi, V. B. Chen, I. W. Davis, N. Echols, J. J. Headd, L.-W. Hung, G. J. Kapral, R. W. Grosse-Kunstleve, A. J. McCoy, N. W. Moriarty, R. Oeffner, R. J. Read, D. C. Richardson, J. S. Richardson, T. C. Terwilliger, P. H. Zwart, PHENIX: A comprehensive Python-based system for macromolecular structure solution. *Acta Crystallogr. D Biol. Crystallogr.* **66**, 213–221 (2010).

89. A. Urzhumtsev, P. V. Afonine, P. D. Adams, TLS from fundamentals to practice. *Crystallogr. Rev.* **19**, 230–270 (2013).

90. S. C. Lovell, I. W. Davis, W. B. Arendall III, P. I. W. de Bakker, J. M. Word, M. G. Prisant, J. S. Richardson, D. C. Richardson, Structure validation by Cα geometry: ϕ, ψ and Cβ deviation. *Proteins* **50**, 437–450 (2003).

91. P. Hou, W. Hao, B. Qin, M. Li, R. Zhao, S. Cui, Structural and biochemical characterization of Schlafen11 N-terminal domain. *Nucleic Acids Res.* **51**, 7053–7070 (2023).

92. J. Pei, B.-H. Kim, N. V. Grishin, PROMALS3D: A tool for multiple protein sequence and structure alignments. *Nucleic Acids Res.* **36**, 2295–2300 (2008).

93. J. Pei, N. V. Grishin, PROMALS3D: Multiple protein sequence alignment enhanced with evolutionary and three-dimensional structural information. *Methods Mol. Biol.* **1079**, 263–271 (2014).

94. A. M. Waterhouse, J. B. Procter, D. M. A. Martin, M. Clamp, G. J. Barton, Jalview Version 2—A multiple sequence alignment editor and analysis workbench. *Bioinformatics* **25**, 1189–1191 (2009).

95. G. Erdős, M. Pajkos, Z. Dosztányi, IUPred3: Prediction of protein disorder enhanced with unambiguous experimental annotation and visualization of evolutionary conservation. *Nucleic Acids Res.* **49**, W297–W303 (2021).

96. K. T. O'Brien, C. Mooney, C. Lopez, G. Pollastri, D. C. Shields, Prediction of polyproline II secondary structure propensity in proteins. *R. Soc. Open Sci.* **7**, 191239 (2020).

97. M. Varadi, D. Bertoni, P. Magana, U. Paramval, I. Pidruchna, M. Radhakrishnan, M. Tsenkov, S. Nair, M. Mirdita, J. Yeo, O. Kovalevskiy, K. Tunyasuvunakool, A. Laydon, A. Žídek, H. Tomlinson, D. Hariharan, J. Abrahamson, T. Green, J. Jumper, E. Birney, M. Steinegger, D. Hassabis, S. Velankar, AlphaFold Protein Structure Database in 2024: Providing structure coverage for over 214 million protein sequences. *Nucleic Acids Res.* **52**, D368–D375 (2024).

98. C. Elfmann, J. Stülke, PAE viewer: A webserver for the interactive visualization of the predicted aligned error for multimer structure predictions and crosslinks. *Nucleic Acids Res.* **51**, W404–W410 (2023).

99. D. Vasudevan, E. Y. D. Chua, C. A. Davey, Crystal structures of nucleosome core particles containing the ‘601’ strong positioning sequence. *J. Mol. Biol.* **403**, 1–10 (2010).

100. L. N. Shen, C. Dong, H. Liu, J. H. Naismith, R. T. Hay, The structure of SENP1-SUMO-2 complex suggests a structural basis for discrimination between SUMO paralogues during processing. *Biochem. J.* **397**, 279–288 (2006).

101. D. Reverter, C. D. Lima, A basis for SUMO protease specificity provided by analysis of human Senp2 and a Senp2-SUMO complex. *Structure* **12**, 1519–1531 (2004).

102. Y. Li, A. De Bolòs, V. Amador, D. Reverter, Structural basis for the SUMO2 isoform specificity of SENP7. *J. Mol. Biol.* **434**, 167875 (2022).

103. E. Mossessova, C. D. Lima, Ulp1-SUMO crystal structure and genetic analysis reveal conserved interactions and a regulatory element essential for cell growth in yeast. *Mol. Cell* **5**, 865–876 (2000).

104. T. D. Goddard, C. C. Huang, E. C. Meng, E. F. Pettersen, G. S. Couch, J. H. Morris, T. E. Ferrin, UCSF ChimeraX: Meeting modern challenges in visualization and analysis. *Protein Sci.* **27**, 14–25 (2018).

105. D. Reverter, C. D. Lima, Structural basis for SENP2 protease interactions with SUMO precursors and conjugated substrates. *Nat. Struct. Mol. Biol.* **13**, 1060–1068 (2006).
